# The effect of the “Follow in my Green Food Steps” programme on cooking behaviours for improved iron intake: a quasi-experimental randomized community study

**DOI:** 10.1186/s12966-018-0710-4

**Published:** 2018-08-16

**Authors:** René Lion, Oyedunni Arulogun, Musibaau Titiloye, Dorothy Shaver, Avinish Jain, Bamsa Godwin, Myriam Sidibe, Mumuni Adejumo, Yves Rosseel, Peter Schmidt

**Affiliations:** 1 Unilever R&D Vlaardingen, Vlaardingen, The Netherlands; 2 Department of Health Promotion & Education, College of Medicine, University of Ibadan, Ibadan, Nigeria; 3 Unilever Nigeria, Lagos, Nigeria; 4 Social Mission Unilever Africa, Nairobi, Kenya; 5 Department of Environmental Health Sciences, College of Medicine, University of Ibadan, Ibadan, Nigeria; 6 Department of Data Analysis, University of Ghent, Gent, Belgium; 7 University of Giessen & Humboldt Research Fellow at Cardinal Wyscinski University Warsaw, Warszawa, Poland

**Keywords:** Behaviour change, Theory of planned behaviour, Dietary change, Iron fortification

## Abstract

**Background:**

Nutritional iron deficiency is one of the leading factors for disease, disability and death. A quasi-experimental randomized community study in South-West Nigeria explored whether a branded behaviour change programme increased the use of green leafy vegetables (greens) and iron-fortified bouillon cubes in stews for improved iron intake.

**Methods:**

A coinflip assigned the intervention to Ile-Ife (Intervention town). Osogbo (Control town) received no information. At baseline 602 mother-daughter pairs (daughters aged 12–18) were enrolled (Intervention: 300; Control: 302). A Food Frequency Questionnaire assessed the addition of cubes and greens to stews, the primary outcome. Secondary outcomes were the addition of cubes and greens to soups and changes in behavioural determinants measured using the Theory of Planned Behaviour. Structural Equation Modelling (SEM) evaluated the impact of the intervention on behavioural determinants and behaviour.

**Results:**

The data of 527 pairs was used (Intervention: 240; Control: 287). The increase in greens added to stews was larger in the Intervention town compared to the Control town (M_Intervention_ = 0.3 [SE = 0.03]; M_Control_ = 0.0 [SE = 0.04], *p* < 0.001, *r* = 0.36). Change in iron-fortified cubes added to stews did not differ between towns (*p* = 0.07). The increase in cubes added to soups was larger in the Intervention town compared to the Control Town (M_Intervention_ = 0.9 [SE = 0.2] vs M_Control_ = 0.4 [SE = 0.1], *p* < .0001, *r* = 0.20). Unexpectedly, change in greens added to soups was larger in the Control town compared to the Intervention town (M_Intervention_ = − 0.1 [SE = 0.1]; M_Control_ = 0.5 [SE = 0.1], *p* = 0.003, *r* = 0.15). The intervention positively influenced awareness of anaemia and the determinants of behaviour in the Intervention town, with hardly any change in the Control town. Baseline SEMs could not be established, so no mediation analyses were done. Post-intervention SEMs highlighted the role of habit in cooking stews.

**Conclusions:**

The behaviour change programme increased the amount of green leafy vegetables added to stews and iron-fortified cubes added to soups. Future research should assess the long-term impact and the efficacy of the programme as it is scaled up and rolled out.

**Electronic supplementary material:**

The online version of this article (10.1186/s12966-018-0710-4) contains supplementary material, which is available to authorized users.

## Background

Anaemia is considered the most prevalent of nutritional deficiencies, estimated to affect 1.62 billion people globally and especially non-pregnant women (496 million) [1]. It has been associated with impaired cognitive and motor development, fatigue and low productivity [1]. In Central and West Africa, where approximately half of the women suffer from it, prevalence appears to be highest [1] with roughly half of the cases due to iron deficiency [2, 3]. Nutritional iron deficiency has been identified as one of the 10 leading factors for disease, disability and death [4].

Three approaches have been proposed to combat nutritional iron deficiency. The first and preferred approach is to modify the diet to improve the nutritional value and iron bio-availability, for example by providing dietary advice to include iron-rich foods and improve cooking skills. This can be challenging as changing behaviours is not easy and there may be practical limitations such as the availability of iron-rich foods. The second is to provide iron supplements (i.e., through pills), but this is relatively expensive and suffers from lack of compliance [5, 6]. In practice, the third approach – micronutrient fortification of regularly consumed processed foods – is a practical and cost-effective long-term solution [5].

Bouillon cubes, powders and seasonings are used across the world and are a good micronutrient fortification vehicle, as they are regularly used in cooking dishes that are consumed by the whole family. As one of the largest bouillon brands in the world, Knorr/Royco can impact the lives of millions of people across the world by fortifying its bouillon cubes [7]. The launch of Knorr iron-fortified bouillon cubes in 2015 was combined with a branded behaviour change programme called “Follow in my Green Food Steps” that aims to increase people’s iron intake. Thus, the two preferred methods of improving dietary intake of iron, i.e., modification of the diet and fortification of processed foods have been combined. The programme focused on mothers and daughters as they are the most vulnerable to iron deficiency and cooking behaviours are passed on from one generation to the next. The ambition is to roll out the programme globally in countries where anaemia is prevalent. The programme was piloted in Nigeria, one of the biggest bouillon markets in the world, with an average consumption of 21 g bouillon cubes per week [8] and a country where a significant proportion of the population does not meet the recommended daily intake of 20 mg of iron per day [3, 9–12].

“Follow in my Green Food Steps” is a six-week home and school-based community programme for mothers and daughters, which aims to motivate them to add green leafy vegetables and Knorr iron-fortified bouillon cubes to a commonly consumed dish, Nigerian beef stew. The programme employs the Lifebuoy hygiene programme framework which has been shown to be effective [13] and has been rolled out across the world. The Lifebuoy framework uses both home and school settings to communicate the main messages of the programme by means of trained promotors. The programme integrates Behaviour Change Techniques that have been proven to be effective in several meta-analytical reviews of life-style change and a change in food intake behaviours [14–21].

An under-investigated aspect in research on behaviour change is the assumption that changes in the determinants of behaviour are associated with changes in intention and subsequently behaviour [22–26]. Behavioural determinants were measured at baseline and post-intervention to assess this. The Theory of Planned Behaviour (TPB) has proven to be a parsimonious and useful model to explain the determinants of behaviour and when applied in a theory-consistent way can deliver insightful results [18, 27–35]. Numerous studies in (sub-Saharan) Africa - including Nigeria - support the use of TPB and behaviour change techniques across a variety of behaviours [36–41].

This paper describes a quasi-experimental randomised community study that explored the effectiveness of an intervention to change how people cook their dishes. The primary measure assessed the addition of iron-fortified bouillon cubes and green leafy vegetables to stews. The secondary measures assessed whether the behaviour also spread to soups and whether changes in behavioural determinants mediated the change in behaviour. Finally, the study explored whether the programme increased the interaction between mothers and daughters - which the programme used as a means to communicate information about iron and anaemia - and if it led to an improvement in self-reported health status. This study is unique, in that behavioural determinants and food preparation behaviour in a non-Western population were assessed at both baseline and post-intervention and were modelled using Structural Equation Modelling (SEM) [22, 26, 42–44].

## Methods

### Study design

The study employed a quasi-experimental pre- and post-intervention design with a randomly assigned intervention and control town. As there is no reliable census data in Nigeria, local knowledge was used to identify Ile-Ife and Osogbo in Osun State in the South-West of Nigeria as the study sites. Both are medium-sized university towns with comparable demographics, approximately 3 to 4 hours north of Lagos. By road, they are approximately 50 km apart or just over a 1 hour’s drive and cross-contamination between the intervention and control town was unlikely. Allocation to the intervention or control arm was decided by the flip of a coin: participants in Ile-Ife received the intervention. Participants in Osogbo did not receive any intervention. The baseline measurement was conducted in February 2016, with the post-intervention measurement conducted in early July 2016, approximately 1 week after the intervention was finished.

### Participants

**Fig. 1:**
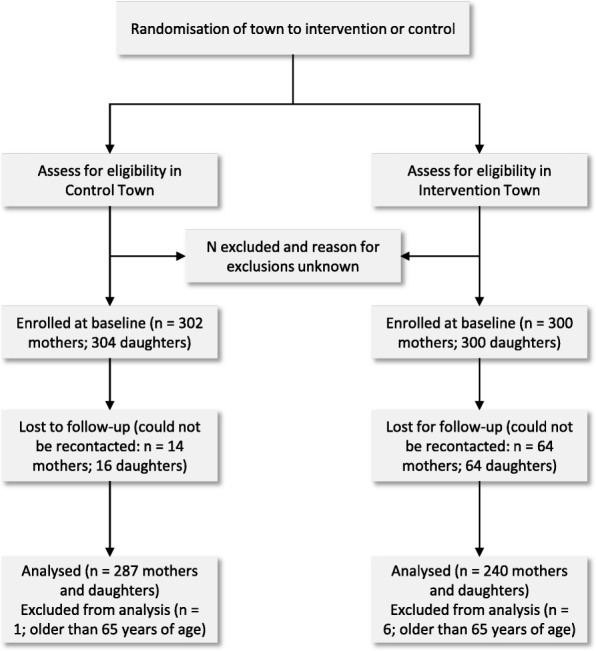
CONSORT flow chart for the “Follow in my Green Food Steps” randomized community study

Participants were eligible if the mother was literate, had a biological daughter between 12 and 18 years of age that lived in the same house and attended secondary school, was co-responsible for cooking and daily grocery shopping, had an activated phone, was available for the course of the study (approximately 12 weeks from recruitment) and was not on any diet. At baseline, 1204 mothers and daughters were recruited (302 mothers and daughters in the control town and 300 mothers and daughters in the intervention town, respectively). Post-intervention, 1068 could be re-contacted (Control: 288 pairs; Intervention: 246 pairs), resulting in an attrition of 11.4%. Based on the demographics, mothers older than 68 years old were excluded, as that would imply that they would have been at least 50 years old at the birth of their 18-year-old daughter. This led to the elimination of 7 mothers from the dataset (Intervention: 6; Control: 1). This relatively relaxed cut-off point was chosen, as particularly with the older generations, age and birth dates in Nigeria are not as exact as in most Western countries. Figure 1 provides an overview in line with CONSORT guidelines (see also Additional file 1 for the CONSORT Checklist) [45].

### Procedure

#### Recruitment

In the Control town (Osogbo), the research assistants went door-to-door to ask whether people were willing to participate in the study. They were checked for eligibility according to the recruitment criteria. In the Intervention town (Ile-Ife), potential participants were pre-recruited by the team delivering the intervention at the schools. The mothers received a sign-up form from their daughters to agree to their participation in the behaviour change programme. This form also included a separate line asking them whether they agreed to be contacted by the research teams from Ibadan university to participate in research on food intake behaviours. The university’s research teams recruited participants based on the list of names and addresses of mothers that had agreed to be contacted for the research. No data was collected from participants that did not agree to participate. The Informed Consent forms were read out aloud to the participants by the interviewers. The name of the study was not read out.

### Sample size

As neither the intervention nor the food intake measure (see below) had been used prior to this study, sample size was estimated under the assumption that adding green leaves to a dish was a binomial distribution (Additional file 2: Table S1). Sample size was estimated to detect a difference of one bunch of green leafy vegetables added to stew per week, with an alpha of 0.05 and a power of 0.8, leading to final sample size of between 166 and 300 respondents per group. Allowing for attrition, we aimed for 400 mother and daughter pairs per group at baseline.

### Research teams

The research team from Ibadan University consisted of the principal investigator (OA) and the co-investigator (MT). Each town had a subteam consisting of eight interviewers, one supervisor and one logistics officer. Experienced interviewers were recruited with at least a National Diploma certificate. The supervisors were Master of Public Health graduates from Ibadan University. All team members received a two-day training at Ibadan University about the purpose of the study, the required interview methodology and how to acquire an informed consent. The research teams operated in pairs, so that both mother and daughter could be interviewed simultaneously.

### Materials

#### The intervention

**Fig. 2:**
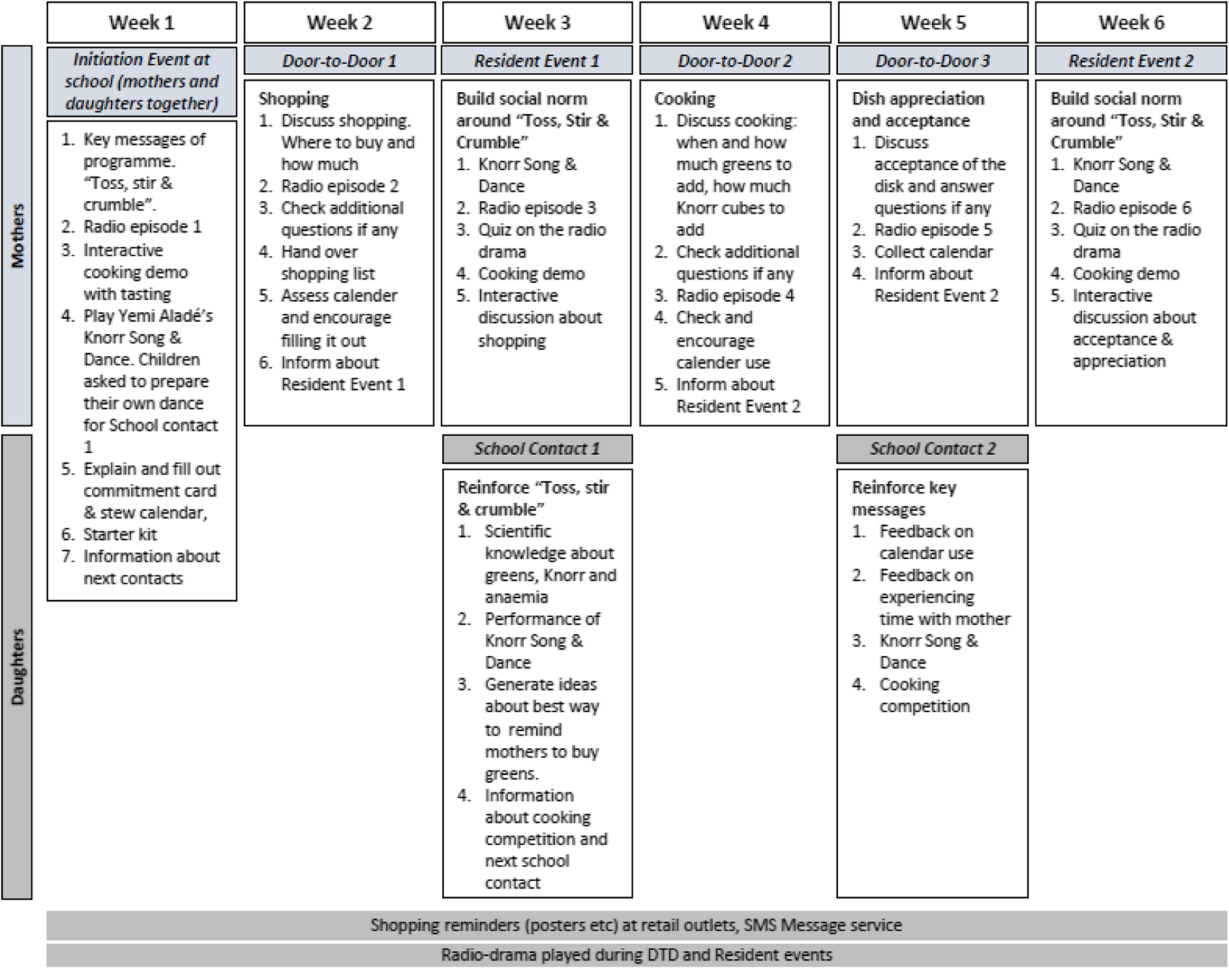
Schematic overview of the behaviour change programme

A detailed description of the flow of the programme and the elements involved is provided in Fig. 2

Table 1 shows the Behaviour Change Techniques (BCTs) [46, 47] that were used in the programme and their link to the theoretical constructs as defined by Cane et al. [48].

**Table 1:** Behaviour Change Techniques used in the Follow in my Green Food Steps programme, based on BCT Taxonomy (v1) [47]

Theoretical Framework Domain^a^	BCT Grouping	BCT	Programme Element
Goals [9] Intentions [8]	Goals and Planning	1.1. Goal setting (behaviour)	Initiation event Promise letter
		1.8 Behavioural contract	Promise letter
		1.9 Commitment	Promise letter
Behavioural Regulation [14]	Feedback & Monitoring	2.1 Monitoring of behaviour by others without feedback	Stew calender
		2.3 Self-monitoring of behavior	Stickers for tracking
Beliefs about capabilities [4] Skills [2]	Shaping Knowledge	4.1 Instructions on how to perform the behaviour	Cooking demonstration
Beliefs about consequences [6] Knowledge [1]	Natural Consequences	5.1 Information about health consequences	Initiation event Radio drama Song & Dance
Beliefs about capabilities [4] Skills [2]	Comparison of behaviour	6.1 Demonstation of behaviour	Video with Omotola and daughter
Memory, Attention & Decision Processes [10]	Associations	7.1 Prompts/Cues	Shopping list Shopping reminders
Reinforcement [7]	Reward & Threat	10.3 Non-specific reward	Ankara/school bag prize
Resources/Material resources [11]	Antecedents	12.5 Adding objects to the environment	Goody Bag with iron-fortified bouillon cubes and a bunch of pumpkin leaves

*BCT* Behaviour Change Technique

^a^Theoretical Framework Domain, based on a synthesis of theoretical constructs that have been identified in theories related to behaviour change [48]

The programme was delivered by trained programme ambassadors, who received an intensive two-day training that included an explanation of the purpose of the programme, the materials included as well as role play to practice the sessions they were to lead.

The well-known Nigerian actress Omotola and her daughter Meriah role-modelled the new behaviours in a video. The behaviour was summarised in three simple steps: toss in a handful of green leafy vegetables, stir these in and crumble in bouillon cubes, shortened to “Toss, stir and crumble”). A catchy tune written by local pop star Yemi Aladé that incorporated the key messages was combined with a dance acting out the call to action to help engrain the behaviours in a fun and engaging way. A story line about iron deficiency was played through a radio programme, which helped root the problem in their reality. Furthermore, participants could engage in a mobile text-message-based subscription service that would send messages and reminders to those who had subscribed to it, addressing barriers in shopping, cooking and family appreciation of the new behaviour, but this did not work as intended due to technical issues. At the initiation event, mothers received a “goody bag” with 6 packs of Knorr iron-fortified bouillon cubes (2 cubes per pack) and a bunch of green leafy vegetables (enough for one dish).

### Measures

Table 2 provides an overview of the questionnaires. No other measures were taken. The questionnaires are available as supplementary material (Additional files 3 and 4). During the training of the research team, the questionnaires were fine-tuned for clarity, ease of use and appropriate translation to the local language (Yoruba). They were also pilot-tested on these aspects in a community close to Ibadan University. Following these pilot interviews, the questionnaires were further refined. Due to time and cost constraints, no separate reliability and validity assessments were conducted for these measures, which were specifically constructed for this study.

**Table 2:** Overview of the structure of the questionnaire and which questions were presented to mothers and daughters

Sections	Topics	Number of items	response scale	Table
1	Demographics^a^^b^	11	n.a.	Table 2
2	General shopping, living and cooking behaviours^a^^b^	16	miscellaneous	not reported
3	Food Intake Questionnaire		n.a.	
4	Knowledge - symptoms	5	1 = less than once a month or never; 2 = 1–3 times a month; 3 = once a week; 4 = 3–5 times a week; 5 = once a day; 6 = More than once a day	Additional file 2, Table S6
	Knowledge - awareness	1	yes/no	
	Knowledge - condition/situations that increase risk	25	yes/no	not reported
	Knowledge - role of iron	13	1 = disagree strongly; 2 = disagree slightly; 3 = neither agree/nor disagree; 4 = agree slightly; 5 = agree strongly	not reported
	Knowledge - effective solutions	16	yes/no	not reported
	Knowledge - good sources of iron	10	yes/no	not reported
5	Determinants of Behaviour - adding cubes to stews	24	5-point scales. Mosty: 1 = disagree strongly; 2 = disagree slightly; 3 = neither agree/nor disagree; 4 = agree slightly; 5 = agree strongly	Additional file 2, Table S3
	Determinants of Behaviour - adding greens to stews	24	5-point scales. Mostly: 1 = disagree strongly; 2 = disagree slightly; 3 = neither agree/nor disagree; 4 = agree slightly; 5 = agree strongly	Additional file 2, Table S4
6	Mother-daughter interaction	7	1 = less than once a month or never; 2 = 1–3 times a month; 3 = once a week; 4 = 3–5 times a week; 5 = once a day; 6 = more than once a day	Additional file 2, Table S5
7	Post-evaluation questionnaire^a^^c^	62	miscellaneous	not reported

^a^only mothers were asked these questions

^b^only included in the baseline questionnaire

^c^only included in the post-intervention questionnaire

#### Food intake questionnaire

The objective was to assess average intake of two specific categories of dishes (stews and soups) over a period of time. A short, focused Food Frequency Questionnaire (FFQ) was created, which was tailored to the purpose of this study and relevant to Nigerian context [49–55]. It focused on stews and soups, the two main dish categories in Nigeria. Participants were first asked to indicate how often they had prepared different types of stews in the past 2 weeks. Then, for each of the stews they indicated which ingredients they had added and how much, based on a pre-specified list of stews and ingredients. Quantity assessments were based on locally used units. Respondents filled out similar questions for soups as they had done for stews. The intake questionnaire ended with a question about how often they had prepared several (side) dishes (e.g., different variants of rice) in the past 2 weeks (not reported here).

#### Determinants of behaviour

The determinants of behaviour were measured for the two key behaviour of interest, i.e., “adding Knorr iron-fortified cubes to beef stew” and “adding green leafy vegetables to beef stew” [56]. The questionnaire included items measuring Attitude, Injunctive Social Norm (separately for their husband/father and for important others), Descriptive Social norms [57, 58], Perceived Behavioural Control, Habit (taken from the Self-Reported Habit Index [20, 59, 60]) and Intention.

### Ethical clearance

The study was approved by the University of Ibadan/University College Hospital Ethics Committee under registration number NHREC/05/01/2008a (Additional file 5).

### Data entry and analyses

All data was collected using paper and pencil questionnaires. Upon completion of the fieldwork, the data was entered into an SPSS file and checked for errors by running frequencies and descriptives. All analyses, except Structural Equation Modelling, were done with JMP 11.0.0 [61]. Structural Equation Modelling was performed using lavaan 0.5–23 [62] and semTools 0.4–14 [63] under RStudio 1.1.383 [64] with R 3.4.2 [65].

For each respondent, the mean number of iron-fortified bouillon cubes and bunches of green leafy vegetables per 2 weeks was calculated by multiplying the frequency with which each of the stews was prepared by the number of units added to each of the stews across all stews.^1^ The same was done for soups. The intake data was highly skewed and some of the values were rather high. However, there was no clear cut-off point for selecting outliers, so all data was retained. The intake data was log-transformed to reduce skewness. Analyses were conducted on both the original data and the log-transformed data and yielded comparable results. All results reported are on the non-log-transformed data for ease of interpretation.

The two towns had baseline differences, which an analysis of covariance (ANCOVA) does not sufficiently attenuate for [66–69]. Analyses were therefore conducted using both change scores (non-parametric Wilcoxon signed-rank tests) and ANCOVAs. Significance levels were set at 0.05. Effect-sizes for non-parametric tests were calculated based on the log-transformed data, using *r* = z/sqrt(N) [70]. The outcomes of the analyses were comparable. Results of the Wilcoxon signed-rank test have been presented in the text. Where the analyses yielded different outcomes, this has been indicated.

For the SEM analyses we first used Confirmatory Factor Analyses to create measurement models for “adding iron-fortified cubes to stews” and “adding green leafy vegetables to stews” separately for the baseline and for the post-intervention data.^2^ Based on factor loadings of the items, the models at baseline and post-intervention were harmonised, i.e., if an indicator performed well at baseline and not at post-intervention or vice-versa, this indicator was deleted. In a second step, measurement equivalence was assessed across both towns, both for the baseline and post-intervention model. In the third step, measurement equivalence was assessed between baseline and post-intervention, again separately for both behaviours. In a final step, the structural model was created to determine whether the determinants impacted the behaviours, and whether change in the determinants mediated the change in the behaviour. For all analyses, generally accepted cut-off values were chosen: CFI > = 0.95, RMSEA <= 0.09 and SRMR <= 0.08 [43, 71, 72]. All analyses were done using Robustified Maximum Likelihood (MLR) estimation, using Full Information Maximum Likelihood (FIML) estimation for missing data. All other variables (e.g., mother-daughter interaction, self-reported health) were analysed using Wilcoxon signed rank test on the change scores of the dependent measures, with Bonferroni correction for multiple comparisons.

## Results

In total, 527 mothers were included, 240 in the Intervention town and 287 in the Control town.

Mean age (in years) of the mothers was similar across both towns (M_Control_ = 41.3, SE = 0.43; M_Intervention_ = 41.9, SE = 0.53, ChiSq (1) = 0.01, *p* = 0.91). Daughters in the Intervention town were half a year younger compared to the daughters in the Control town, M_Control_ = 14.7, SE = 0.10; M_Intervention_ = 15.2, SE =0.11, ChiSq (1) = 15.52, *p* < .0001). Table 3 shows the demographics of the samples in both towns. There were some differences between the towns in that participants in the Control town had a somewhat higher income and level of education. However, including these variables in the analyses as covariates led to similar results (results not shown).

**Table 3:** Demographics for both towns (post-intervention dataset)

	Control (Osogbo)		Intervention (Ile-Ife)	
Employment	n	%	n	%
Unskilled workers	18	6.3%	8	3.3%
Housewife	4	1.4%	8	3.3%
Farming	179	62.4%	168	70%
Skilled workers (e.g., mechanics, tailoring, carpenters)	36	12.5%	22	9.2%
Civil servant, Clerical workers, teachers etc.	32	11.2%	21	8.8%
Health professionals (e.g., Doctor, Nurse, Community health workers)	7	2.4%	7	2.9%
Professionals (e.g., Lawyer, Engineers, Surveyors)	1	0.4%	1	0.4%
Unemployed	3	1.1%	0	0%
Others (Retired)	7	2.4%	5	2.1%
Marital Status				
Married/living together with partner	255	88.9%	202	84.2%
Widowed	14	4.9%	24	10%
Divorced	9	3.1%	11	4.6%
Married but not living together due to work	9	3.1%	2	0.8%
Missing (No Response)	0	0%	1	0.4%
Education				
Primary Incomplete	17	5.9%	19	7.9%
Primary complete	51	17.8%	48	20%
Secondary Incomplete	39	13.6%	65	27.1%
Secondary complete	115	40.1%	84	35%
Polytechnic or College of Education: OND or NCE	47	16.4%	20	8.3%
University/polytechnics: HND	12	4.2%	2	0.8%
Post University Complete	6	2.1%	0	0%
Can’t read or write/None	0	0%	2	0.8%
Income				
< N10,000	50	17.4%	55	22.9%
Between N10,000 - N20,000	99	34.5%	105	43.8%
Between N20,000 - N50,000	93	32.4%	59	24.6%
Between N50,000 - N100,000	31	10.8%	18	7.5%
More than N100,000	11	3.8%	2	0.8%
Missing (No Response)	3	1.1%	1	0.4%

*OND* Ordinary National Diploma, *NCE* Nigerian Certificate of Education, *HND* Higher National Diploma, *N* Naira (Nigerian currency)

### Primary outcomes

As can be seen in Table 4, at baseline the number of participants adding bouillon cubes to stews was high in both towns, with participants in the Control town added fewer cubes to stews compared to the Intervention town. Following the intervention there was no difference between the towns in the number of participants that started adding bouillon cubes to stews (an additional 6% in the Control town and an additional 7% in the Intervention town), nor in the change of the number of cubes added to stews,^3^ indicating that the intervention did not impact the behaviour to add iron-fortified bouillon cubes to stews (M_diff_Control_ = 0.8, SE = 0.20 vs M_diff_Intervention_ = 0.7, SE = 0.21, ChiSq (1) = 0.07, *ns*).

**Table 4:** Percentage of respondents adding iron-fortified cubes and green leafy vegetables to stews and soups and mean number of iron-fortified cubes and bunches of green leafy vegetables added to stews and soups (per 2 weeks)

		Percentage of participants^a^		Mean^b^ (se)						
		Control	Intervention	Control		Intervention		ChiSq (df)	*p*-value	*r*
Adding Cubes to stews	Baseline	76%	93%	2.5	(0.2)	2.9	(0.2)	15.3	< 0.0001	
	*Change*	*6%*	*7%*	*0.8*	*(0.2)*	*0.7*	*(0.2)*	*0.1*	*ns*	
Adding Greens to stews	Baseline	5%	3%	0.1	(0.03)	0.0	(0.0)	1.7	ns	
	*Change*	*5%*	*41%*	*0.02*	*(0.04)*	*0.30*	*(0.03)*	*68.9*	<.0001	0.36
Adding Cubes to soups	Baseline	76%	71%	1.4	(0.1)	2.0	(0.2)	1.21	*ns*	
	*Change*	*2%*	*28%*	*0.4*	*(0.1)*	*0.9*	*(0.2)*	*17.9*	<.0001	0.20
Adding Greens to soups	Baseline	85%	83%	1.4	(0.1)	1.8	(0.1)	6.0	0.01	
	*Change*	*10%*	*13%*	*0.5*	*(0.1)*	*−0.1*	*(0.1)*	*13.4*	0.0003	*0.15*

*se* standard error

^a^Percentage of participants adding cubes or greens to their dishes

^b^Mean number of cubes or bunches of greens added per 2 weeks

At baseline, very few participants added a bunch of green leafy vegetables to beef stew (Table 4). After the intervention, an additional 5% started adding green leafy vegetables to stews in the Control town whereas an additional 41% in the Intervention town performed the behaviour. The observed change in mean bunches of green leafy vegetables added to stews was also significantly larger in the Intervention town compared to the Control town: M_diff_Control_ = 0.0, SE = 0.04 vs M_diff_Intervention_ = 0.3, SE = 0.03, ChiSq (1) = 69.92, *p* < .0001, *r* = 0.36. The participants adding green leafy vegetables in the Intervention town indicated they added on average 0.8 bunch post-intervention. These results indicate that the intervention was effective in motivating participants to add green leafy vegetables to stews.

### Secondary outcomes

At baseline, the majority of the participants added cubes to soups, with no difference between the groups (Table 4). After the intervention, an additional 28% of the participants started adding bouillon cubes to soups in the Intervention town vs an additional 2% in the Control town. Moreover, the increase in average number of bouillon cubes added to soups was significantly higher in the Intervention town compared to the Control town (M_diff_Control_ = 0.4, SE = 0.12 vs M_diff_Intervention_ = 0.9, SE = 0.18; ChiSq (1) = 23.70, *p* < .0001, *r* = 0.20). These results suggest that the programme influenced participants’ behaviour to add iron-fortified bouillon cubes to their soups.

Most participants indicated they added green leafy vegetables to soups at baseline (Table 4), but fewer participants in the Control town did this compared to the Intervention town. After the intervention, the number of participants that started adding green leafy vegetables to soups increased in both towns. The change from baseline differed significantly between both towns, with an unexpected increase in intake of green leafy vegetables in the Control town vs. no change in the Intervention town (M_diff_Control_ = 0.5, SE = 0.05 and M_diff_Intervention_ = −.01, SE = 0.12, ChiSq (1) = 8.63, *p =* 0.003, *r* = 0.15). These findings suggest that the programme did not impact participants’ use of green leafy vegetables in soups, but we will return to this in the discussion.

### Behavioural determinants

There was a significant difference in awareness at baseline between both towns, with awareness at 70% for mothers in the Control town and 34% in the Intervention town. After the intervention, 63% of the participants indicated they were aware of anaemia in the Control town, whereas 79% of participants in the Intervention town was aware of anaemia, indicating that the programme was effective in increasing awareness.

The final measurement models are provided as additional files (Additional file 6, Figures A and B). Table 5 shows that the baseline models for both “Adding cubes to stews” and “Adding green leafy vegetables” did not converge to well-fitting models, but the fit-indices for the post-intervention models were reasonably good.

**Table 5:** Fit indices for the post-intervention measurement model

	Baseline		Post-Intervention	
	Cubes	Green Leafy Vegetables	Cubes	Green Leafy Vegetables
CFI (Robust)	0.87	0.88	0.94	0.96
RMSEA (Robust)	0.08	0.12	0.08	0.09
SRMR	0.06	0.05	0.05	0.04

*CFI* Comparitive Fit Index, *RMSEA* Root Mean Square Error of Approximation, *SRMR* Standardized Root Mean Square Residual

Cut-off values: CFI > 0.95; RMSEA <= 0.09; SRMR <= 0.08

As a consequence, the baseline models were not used and only the post-intervention structural models were explored to assess which factors were driving behaviour after the intervention (see Additional file 2: Table S2a and Table S2b for the correlations between the latent variables).

**Fig. 3:**
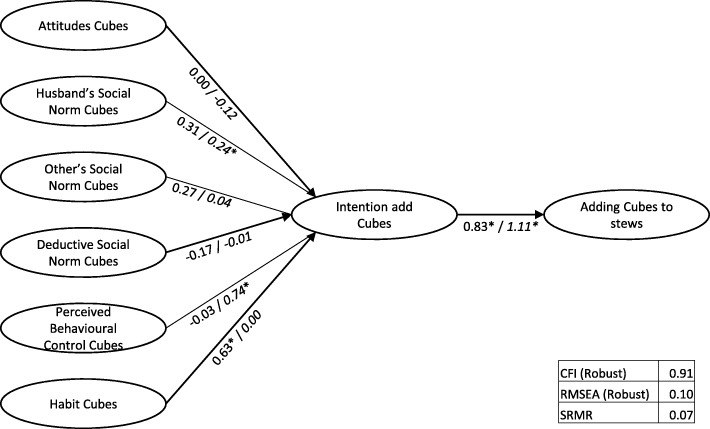
Base Structural Model Cubes (Intervention town/*Control town)*

**Fig. 4:**
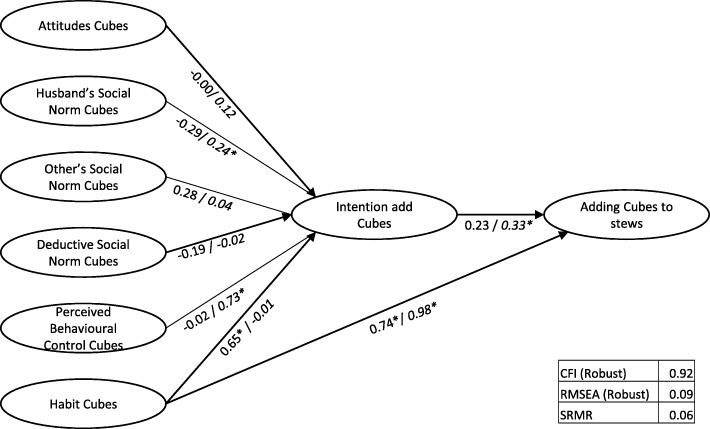
Structural Model Cubes with direct link habit to behaviour (Intervention town/*Control town)*

**Fig. 5:**
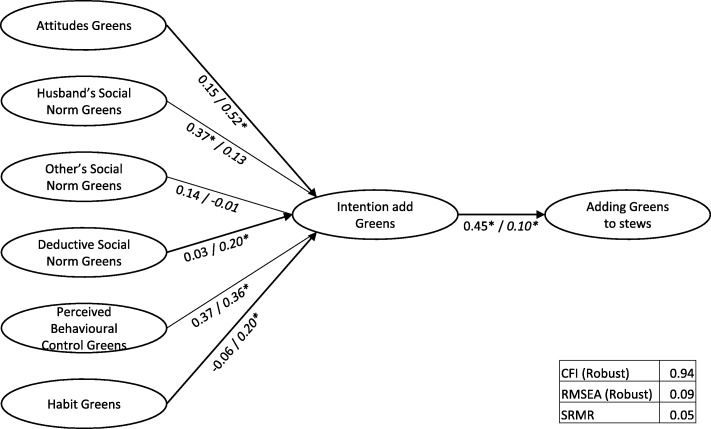
Structural Model Green Leafy Vegetables (Intervention town/ *Control town)*

The structural models are depicted in Figs. 3, 4 and 5, including the regression weights. A base model for Cubes, with intention mediating the relation between the determinants and behaviour had insufficient model fit (CFI = 0.91, RMSEA = 0.10, srmr = 0.07). Adding a direct path from habit to behaviour improved model fit (CFI = 0.92, RMSEA = 0.09, srmr = 0.06). Further adding direct paths between “Perceived Behavioural Control” (PBC) and behaviour did not improve fit. In the Intervention town, when “Habit” was added as a predictor of “Cubes added to stews”, this was the only significant determinant and the relation between “Intention” and the behaviour of adding Cubes did not reach significance. In contrast, in the Control town, “Subjective Norm – Husbands” and “Perceived Behavioural Control” influenced “Intention”, and “Intention” and “Habit” both had a direct relationship with behaviour.

For Green Leafy Vegetables, model fit was reasonable (CFI = 0.94, RMSEA = 0.09, srmr = 0.05). In the Intervention town, “Subjective Norm - Husbands” was the only significant predictor of “Intention to add Green Leafy Vegetables”, with “Intention” significantly impacting “adding Green Leafy Vegetables”. In contrast, in the Control town, “Attitudes”, “Descriptive Norm”, “Perceived Behavioural Control” and “Habit” were significant predictors of “Intention” to “add Green Leafy Vegetables”, with “Intention” a small but significant predictor of the actual behaviour of adding Green Leafy Vegetables to stew. Further adding direct relations between “Intention” and “PBC” and behaviour did not improve model fit.

The differences between pre- and post-intervention scores on the behavioural determinants (Additional file 2: Table S3 and Table S4) showed that for Cubes, the determinants “Subjective Norm - Husband” and “Habit” increased significantly in the Control town, with no change in the Intervention town. For Green Leafy Vegetables, the analyses showed that there was no change in the determinants in the Control town. In the Intervention town, there were clear, significant and favourable changes for the “Subjective Norm - Husband”, “Subjective Norms - Others”, “Descriptive Norm”, “Perceived Behavioural Control”, “Habits” and “Intention” to add Green Leafy Vegetables. These findings suggest that the intervention influenced participants’ determinants of behaviour.

The intervention influenced the interaction between mothers and daughters, in that they discussed topics related to the programme more often. For the mothers, key aspects discussed during the intervention (i.e., “Feeling Dizzy/Tired”, “Cooking Healthily”, “How well she is doing at school” and “How much energy”) showed a significantly larger increase in the Intervention town compared to the Control town. In contrast, the daughters indicated they only discussed “Feeling Dizzy/Tired”, “How well she concentrates” more frequently (Additional file 2: Table S5). Mothers in the Intervention town also reported a significantly larger decline in frequency of experiencing a pale complexion. Daughters in the Intervention town reported a significantly larger decline in frequencies of experiencing “Poor concentration”, “Tiredness” and “Increased irritability” compared to daughters in the Control town (Additional file 2: Table S6).

## Discussion

The objective of the study was to assess the impact of a branded behaviour change programme that aimed to make participants add iron-fortified bouillon cubes and a bunch of green leafy vegetables to stews for improved iron intake.

The results show that the intervention changed behaviour. Forty-one percent of the participants started adding green leafy vegetables to stews in the Intervention town compared to hardly any change in the Control town. The number of participants that started adding iron-fortified cubes to soups and the average amount added increased, suggesting that overall intake of iron should have improved. Although there was no change in the number of iron-fortified cubes that were added to stews, this was likely due to a ceiling effect as a high percentage of participants was already using bouillon cubes. This supports the idea that fortification of bouillon cubes is a good way to increase iron intake in Nigeria. The change in adding green leafy vegetables to soups in the Intervention town was significantly different from that of the Control town, with a surprising increase in the Control town vs no change in the Intervention Town. We interpret this as no change, as intake at baseline in Ile-Ife was much higher than intake in the Control town, whereas at post-intervention they were more similar.

This is the first behaviour change programme targeting the use of iron-fortified bouillon cubes and green leafy vegetables. As it differs from other studies on dietary behaviours, which have tended to focus on fruit & vegetables or weight loss [73–75], comparing effect sizes is difficult. The effect sizes reported in this study are similar or somewhat lower than those reported in meta-analyses on healthy eating [75, 76]. This may be because cube use was already high and adding green leafy vegetables to stews was a completely new behaviour.

The programme is also effective in increasing awareness of anaemia and its symptoms and results in mothers and daughters discussing anaemia and its symptoms. Furthermore, the intervention had a positive impact on several behavioural determinants. It is unfortunate that baseline measurement models could not be identified, but it is not unusual to find that data from longitudinal studies show better fitting models at later time points, which is also called the “Socratic Effect” or “panel conditioning” [77, 78]. The towns in which the study was run were in relatively rural and low-income areas of Nigeria. Thus, their beliefs about using bouillon cubes and adding green leafy vegetables to stews may have been less “crystallised” at baseline and the questionnaire may have led people to contemplate these behaviours. This would especially be the case for “Adding Green Leafy Vegetables”, a behaviour that at baseline was virtually non-existent in either town. The post-intervention SEM models support the finding from the intake data that adding cubes to stews was a common behaviour, with a direct impact of “Habit” on the behaviour of adding cubes to stews, bypassing “Intention”. In fact, in the Intervention town, where use of cubes was even more common than in the Control town, “Habit” appeared to be the only determinant of self-reported behaviour. In contrast, in the Control town, the husband’s beliefs as well as the idea that it was completely up to the cook whether or not to use these bouillon cubes (“Perceived Behavioural Control”) were also determinants of behaviour. For “Adding Green Leafy Vegetables”, the relation between intention and actual behaviour is higher in the Intervention town, with only the husband’s subjective norm as an additional influence. The relation between intention and behaviour was much weaker in the Control town, which may be due to the very low incidence of this behaviour. In addition, intention seemed to be more driven by the mother’s personal attitudes towards adding green leafy vegetables as well as whether she felt it was up to her to add green leafy vegetables to stew (Perceived Behavioural Control). This suggests that determinants of behaviour differ depending on how deeply engrained a behaviour is in people’s lives.

A limitation of this study is that we did not use a validated intake questionnaire to measure the addition of iron-fortified cubes and green leafy vegetables. Instead we opted for a measure specifically adapted for the purpose of this study, using the structure of a short FFQ and ensuring it was suitable for a Nigerian context. The exact same questions were used at baseline and post-intervention, and as we were interested in *change* rather than absolute assessments of intake and only draw conclusions regarding the impact of the intervention in relative terms, the use of a non-validated, but contextually relevant questionnaire seems appropriate. Moreover, market research data available through Unilever validated the frequency of consumption of cubes (internal communication).

As two towns rather than individual participants were randomly assigned to the intervention and control condition, an alternative cause for the intervention effect cannot be ruled out. However, most demographic variables were similar, except for education level and income and controlling for these latter variables did not impact the results of these analyses. Social desirability bias cannot be completely ruled out as participants in the Intervention town may have been aware that this was linked to the Behaviour Change programme running at the schools. Care was taken to ensure that participants saw this as a separate study about food intake behaviours, but it would have been logistically challenging to recruit participants separately from recruitment of the schools, whilst ensuring that all participants also received the behaviour change programme. Moreover, this was mitigated by asking about green leafy vegetables as part of the intake questionnaire, rather than as a separate question that might be more amenable to experimenter demand effects. An alternative explanation for the findings could be a Questionnaire Behaviour Effect (QBE) [79], which seemed to be present in both towns. Given the significant differences in change between both towns, the behaviour change programme appeared to have an effect independent of a QBE.

## Conclusion

This study showed that a branded behaviour change programme can increase awareness of anaemia and change cooking behaviours to help increase intake of iron. Furthermore, the study showed that a large majority cook with bouillon cubes, supporting the idea that these can be a good vehicle for iron fortification. The next step will be to facilitate scaling up and rolling out the programme in a cost-efficient way and to establish the programme’s long-term effects. For the programme to be effective in other markets it will have to be adapted based on local food cultures and dishes. The current programme provides a strong foundation to do this.

## Additional files

Additional file 1: CONSORT 2010 Checklist. (DOC 217 kb)

Additional file 2: Supplementary tables. (DOCX 28 kb)

Additional file 3: Final Baseline Questionnaire. (PDF 1492 kb)

Additional file 4: Post-Intervention Questionnaire. (PDF 1558 kb)

Additional file 5: Revised Protocol for Follow in my Green Food Steps Behaviour Change Impact study. (PDF 1759 kb)

Additional file 6: Measurement Models used for Structural Equation Modelling. (PPTX 66 kb)

## Footnotes

1 We also ran the analyses purely on the amount added per dish for both cubes and leafy green vegetables, yielding comparable results.

2 The determinants were measured specifically about “adding cubes” or “adding green leafy vegetables” to stews, not to soups, so we only used intake on stews as dependent measure.

3 The ANCOVA on the log-transformed data indicated a significant effect of the intervention on total amount of bouillon cubes added to stews, probably due to the insufficient attenuation for the baseline difference [66]).
