# Supplementary material for: The effect of the “Follow in my Green Food Steps” programme on cooking behaviours for improved iron intake: a quasi-experimental randomized community study
Source: Int J Behav Nutr Phys Act. 2018 Aug 16;15:79. doi: 10.1186/s12966-018-0710-4 (PMC6097342; doi:10.1186/s12966-018-0710-4)
Supplement: Supplementary file 2 — Supplementary tables. (DOCX 28 kb) [file 12966_2018_710_MOESM2_ESM.docx]

| **Table 1** Sample size considerations for the quasi-experimental randomised community study | | | | |
| --- | --- | --- | --- | --- |
| Proportion Control group^a^ | Proportion Intervention group^b^ | Alpha | Nominal Power | N Per Group |
| 0.05 | 0.14 | 0.05 | 0.8 | 166 |
|  | 0.28 | 0.05 | 0.8 | 40 |
| 0.07 | 0.14 | 0.05 | 0.8 | 300 |
|  | 0.28 | 0.05 | 0.8 | 51 |
| ^a^ assuming the control group adds 0.35 per 7 days of green leafy vegetables (0.05) or 0.5 per 7 days (0.07) | | | | |
| ^b^ assuming the intervention group starts adding greens 1x every 7 days (0.14) or 2x every 7 days (0.28) | | | | |

| **Table 2a** Correlations among latent variables “Adding Cubes” at post-intervention. | | | | | | | |
| --- | --- | --- | --- | --- | --- | --- | --- |
|  | 1 | 2 | 3 | 4 | 5 | 6 | 7 |
| 1. Attitudes Cubes | 1 |  |  |  |  |  |  |
| 2. Subjective Norm Cubes - Husband | 0.70 | 1 |  |  |  |  |  |
| 3. Subjective Norm Cubes - Others | 0.67 | 0.73 | 1 |  |  |  |  |
| 4. Descriptive Norm Cubes | 0.64 | 0.63 | 0.85 | 1 |  |  |  |
| 5. Perceived Behavioural Control Cubes | 0.74 | 0.65 | 0.74 | 0.72 | 1 |  |  |
| 6. Habit Cubes | 0.50 | 0.55 | 0.64 | 0.62 | 0.69 | 1 |  |
| 7. Intention Cubes | 0.61 | 0.63 | 0.62 | 0.56 | 0.74 | 0.57 | 1 |

| **Table 2b** Correlations among latent variables “Adding Green Leafy Vegetables” at post-intervention | | | | | | | |
| --- | --- | --- | --- | --- | --- | --- | --- |
|  | 1 | 2 | 3 | 4 | 5 | 6 | 7 |
| 1. Attitude Greens | 1 |  |  |  |  |  |  |
| 2. Subjective Norm Greens - Husband | 0.81 | 1 |  |  |  |  |  |
| 3. Subjective Norms Greens - Others | 0.77 | 0.90 | 1 |  |  |  |  |
| 4. Descriptive Norm Greens | 0.75 | 0.86 | 0.90 | 1 |  |  |  |
| 5. Perceived Behavioural Control Greens | 0.75 | 0.81 | 0.84 | 0.78 | 1 |  |  |
| 6. Habit Greens | 0.75 | 0.84 | 0.88 | 0.85 | 0.88 | 1 |  |
| 7. Intention Greens | 0.81 | 0.84 | 0.83 | 0.82 | 0.86 | 0.84 | 1 |

| **Table 3** Means and changes from baseline for behavioural determinants of cubes use. | | | | | | | | |  |
| --- | --- | --- | --- | --- | --- | --- | --- | --- | --- |
|  | Control | | | | Intervention | | | |  |
|  | Baseline (se) | | Post-Intervention change (se) | | Baseline (se) | | Post-Intervention change (se) | |  |
| Attitudes Cubes | 4.2 | (0.03) | 0.3 | (0.04) | 4.5^*^ | (0.03) | 0.2 | (0.04) | |
| Subjective Norm Cubes - Husband | 3.8 | (0.06) | 0.6 | (0.07) | 4.3^*^ | (0.04) | 0.2 | (0.05) | |
| Subjective Norm Cubes - Others | 4.1 | (0.03) | 0.2 | (0.04) | 4.4^*^ | (0.03) | 0.3 | (0.04) | |
| Descriptive Norm Cubes | 3.9 | (0.03) | 0.2 | (0.04) | 4.2^*^ | (0.04) | 0.3 | (0.05) | |
| Perceived Behavioural Control Cubes | 4.2 | (0.03) | 0.2 | (0.04) | 4.3^*^ | (0.03) | 0.2 | (0.04) | |
| Habit Cubes | 3.4 | (0.06) | 0.5 | (0.07) | 4.2^*^ | (0.05) | 0.4 | (0.06) | |
| Intention Cubes | 4.1 | (0.05) | 0.4 | (0.06) | 4.4^*^ | (0.04) | 0.2^*^ | (0.05) | |
| *se*: standard error  ^*^ Significantly different between towns, *p* < .007 (corrected for multiple comparisons) | | | | | | | | | |

| **Table 4** Means and changes from baseline for determinants of adding greens. | | | | | | | | | | |
| --- | --- | --- | --- | --- | --- | --- | --- | --- | --- | --- |
|  | Control | | | | | Intervention | | | | |
|  | Baseline (se) | | Post-intervention change (se) | | | Baseline (se) | | | Post-intervention change (se) | |
| Attitude Greens | 3.6 | (0.08) | 0.2 | (0.08) | 4.4^*^ | | (0.05) | 0.3 | | (0.06) |
| Subjective Norm Greens - Husband | 3.0 | (0.07) | 0.1 | (0.09) | 3.7^*^ | | (0.09) | 0.8^*^ | | (0.10) |
| Subjective Norm Greens - Others | 2.8 | (0.08) | 0.3 | (0.09) | 3.6^*^ | | (0.08) | 0.9^*^ | | (0.09) |
| Descriptive Norm Greens | 2.4 | (0.07) | 0.2 | (0.08) | 3.5^*^ | | (0.07) | 0.8^*^ | | (0.08) |
| Perceived Behavioural Control Greens | 3.0 | (0.07) | -0.1 | (0.09) | 4.1^*^ | | (0.05) | 0.4^*^ | | (0.06) |
| Habit Greens | 2.3 | (0.07) | 0.2 | (0.09) | 3.4^*^ | | (0.08) | 1.0^*^ | | (0.09) |
| Intention Greens | 2.9 | (0.08) | 0.3 | (0.09) | 3.8^*^ | | (0.09) | 0.8^*^ | | (0.09) |
| *se*: standard error  ^*^ Significantly different between towns, *p* < .007 corrected for multiple comparisons | | | | | | | | | | |
|  | | | | | | | | | | |

| **Table 5** Mean change in number of times mothers and daughters discussed topics mentioned in the table. | | | | | | | | |
| --- | --- | --- | --- | --- | --- | --- | --- | --- |
|  | Mothers | | | | Daughters | | | |
| *Topics* | *Control (se)* | | *Intervention (se)* | | *Control (se)* | | *Intervention (se)* | |
| Cooking Skills | 1.3 | (0.08) | 1.2 | (0.09) | -0.4 | (0.14) | 0.1 | (0.13) |
| Cook Healthily | 0.9 | (0.08) | 1.2^*^ | (0.09) | 0.3 | (0.12) | 0.3 | (0.14) |
| Feeling dizzy-tired | 0.0 | (0.12) | 0.7^*^ | (0.12) | -0.3 | (0.12) | 1.1^*^ | (0.16) |
| How doing at school | 0.5 | (0.08) | 0.8^*^ | (0.11) | 0.4 | (0.11) | 0.3 | (0.13) |
| Discusses with her friends | 0.4 | (0.12) | 0.6 | (0.14) | -0.6 | (0.16) | 0.1 | (0.15) |
| How well daughter can concentrate | 0.0 | (0.09) | 0.8^*^ | (0.11) | -0.4 | (0.14) | 0.6^*^ | (0.15) |
| How much energy | -0.2 | (0.02) | 0.4 | (0.17) | -0.4 | (0.16) | 0.1 | (0.16) |
| *se*: standard error  ^*^ significantly different between towns, *p* < .004, significance level corrected for multiple comparisons. Response scale: 1 = “Less than once a month or never” to 6 = “more than once a day” | | | | | | | | |

| **Table 6** Change in frequency of experiencing symptoms of anaemia. | | | | | | | | | |  |
| --- | --- | --- | --- | --- | --- | --- | --- | --- | --- | --- |
|  | Mothers | | | | Daughters | | | |  |  |
|  | Control (se) | | Intervention (se) | | Control (se) | | Intervention (se) | |  |  |
| Poor concentration | -0.1 | (0.10) | -0.1 | (0.09) | -0.2 | (0.11) | -1.5^*^ | (0.19) |  |  |
| Tiredness, even though you have had enough sleep | -0.1 | (0.09) | -0.3 | (0.08) | -0.1 | (0.09) | -0.9^*^ | (0.13) |  |  |
| Increased irritability | 0.0 | (0.10) | -0.2 | (0.09) | 0.0 | (0.10) | -0.4^*^ | (0.10) |  |  |
| Dizziness | 0.0 | (0.06) | -0.2 | (0.10) | -0.1 | (0.05) | -0.1 | (0.08) |  |  |
| Paler complexion | 0.1 | (0.06) | -0.3^*^ | (0.09) | 0.0 | (0.03) | -0.1 | (0.09) |  |  |
| *se*: standard error  ^*^significantly different change compared to Osogbo (control), *p* < .0001. Response scale: 1 = “Less than once a month or never” to 6 = “More than once a day”. | | | | | | | | | | |
