# Supplementary material for: The effect of the “Follow in my Green Food Steps” programme on cooking behaviours for improved iron intake: a quasi-experimental randomized community study
Source: Int J Behav Nutr Phys Act. 2018 Aug 16;15:79. doi: 10.1186/s12966-018-0710-4 (PMC6097342; doi:10.1186/s12966-018-0710-4)
Supplement: Supplementary file 3 — Final Baseline Questionnaire. (PDF 1492 kb) [file 12966_2018_710_MOESM3_ESM.pdf]

TO BE FILLED OUT BY PRINCIPAL INVESTIGATORS ONLY:
UNIQUE ID:

UNIVERSITY OF IBADAN

BASELINE QUESTIONNAIRE

FOLLOW IN MY FOOD STEPS BEHAVIOUR CHANGE IMPACT STUDY

YORUBA VERSION

Reference Number: SPB01_2015

INTERVIEW NO____________

1. Initials of Interviewer:______________ 2. Date (DD/MM/YY): ____/____/2015
3. State:__________________________ 4. LGA: ___________________ PAIRED WITH INT NO: _____
5. Name of Community_______________ 6. Time: Started: ____/____/ (hh/mm)

INTERVIEWER: BEFORE INTERVIEWING THE RESPONDENT, ENSURE YOU HAVE TAKEN HER THROUGH THE INFORMED CONSENT FORM AND HAVE RECEIVED HER APPROVAL AND SIGNATURE.

TO BE FILLED OUT BEFORE STARTING THE INTERVIEW. DO NOT READ THIS OUT.

I, ________________[name of interviewer] declare that I have read the informed consent form to the respondent. I have made clear that
- the respondent's participation is completely voluntary
- all answers provided will be kept confidential and cannot be traced back to the participant
- the respondent is free to stop participating at any time and this will not affect the respondent in any way

Signature of interviewer: ____ Date: ______________________

SECTION 1: DEMOGRAPHICS

[INTERVIEWER, PLEASE READ OUT]

"Ma nife lati bere pelu awon ibeere nipa ebi yin, awon ti ejo ngbe ati jeun papo ninu ikoko kanna."

("I WOULD LIKE TO START WITH A FEW QUESTIONS ABOUT YOUR FAMILY, HOUSEHOLD, EDUCATION ETC. ")

[INTERVIEWERS: CIRCLE, TICK OR FILL IN AS APPROPRIATE]

**S1.** E jowo, e so ojo ori yin? *(Can you tell me how old you are?)*	___________Odun *(years)*

[INTERVIEWER: IF AGE >18 AND **MOTHER** CONTINUE WITH S2;
IF RESPONDENT IS **DAUGHTER**, AND MOTHER IS ELIGIBLE TO PARTICIPATE (AFTER S3), CONTINUE WITH *SECTION 3 : FOOD INTAKE QUESTIONNAIRE*. THE DAUGHTER DOES NOT HAVE TO RESPOND TO S2-S11 AND TO THE QUESTIONS IN SECTION 2].

S2. Nje e le so fun wa bi e se n kopa si ninu awon ise ile ti a ko si isale yi?
(Can you please specify your level of involvement in each of the household tasks below)
[INTERVIEWER: PLEASE INDICATE APPROPRIATE RESPONSE.]

**S1A**. Nje e le so fun wa eniti o ndana ninu idile yin? *(Who is responsible for **cooking** in your household?)*		
1	Emi ni mo n se amojuto eyi ninu idile mi *(I am fully responsible for this in my household)*	
2	Emi ati elomiran ni a nmojuto eyi ninu idile mi *(I am responsible for this together with someone else)*	
3	Emi ko ni mo nse amojuto ise yi *(I am not responsible for this task)*	END

**S1B.** Nje e le so fun wa eniti o nlo soja ra awon nkan obe ninu idile yin? *(Who is responsible for grocery shopping in your household?)*		
1	Emi ni mo n se amojuto eyi ni nu idile mi *(I am fully responsible for this in my household)*	
2	Emi ati elomiran ni a n mojuto eyi ninu idile mi *(I am responsible for this together with someone else)*	
3	Emi ko ni mo wa fun ise yi *(I am not responsible for this task)*	END

[SCREEN-OUT IF NOT (CO-) RESPONSIBLE FOR COOKING AND/OR GROCERY SHOPPING (IF S2_A=3 AND S2_B=3)]

**S3.** Nje e ni omo kankan ti ngbe ni idile yin? *(Do you have any children living in your household?)* [INTERVIEWER: ASK FOR ALL CHILDREN LIVING IN THAT HOUSEHOLD, NOT JUST BIOLOGICAL CHILDREN]			
1	Beeni *(Yes)*		CONTINUE
2	Beeko *(No)*		END

[INTERVIEWER: IF BASED ON QUESTION S1-S3, THE MOTHER IS ELIGIBLE TO PARTICIPATE, THE DAUGHTER CAN NOW CONTINUE WITH SECTION 3.

**S4.** Kini ise yin? *(What is your occupation?)*		
1	Onise owo ti a ko ko *(Unskilled workers)*	
2	Iyawo ile *(Housewife)*	
3	Ise agbe *(Farming)* / Okowo *(Trading/shopkeeper)* / Ise owo *(Artisan)*	
4	Awon onise owo (atu oko se, aranso, gbena gbena, molemole) *(Skilled workers (mechanics, tailoring, carpenters, bricklayers))*	
5	Akowe ofiisi / Ise ijoba / Tisa ati beebe lo *(Civil Servant, Clerical workers, Teacher etc)*	
7	Osise eleto ilera (Dokita, Noosi ati beebe lo) *(Health Professional, e.g., Doctor, Nurse, Nutrionist, community health worker etc)*	
8	Awon akosemose bi agbejoro, onimo ero, wonle wonle ati beebe lo *(Professional e.g. Lawyer, Engineers, Surveyors etc.)*	
9	Alakoso *(Manager)*	
10	Alakoso agba *(Senior Management/Admin.)*	
11	Akeko *(Student)*	
12	Ai nise lowo *(Unemployed)*	
13	Omiran *(Others)*	

**S5.** Eniyan melo lo ngbe idile yin pelu eyin naa?<br>*(How many people live in your household including yourself?*	

**S6.** Awon omo melo ninu gbedeke ojo ori wonyi ni o n gbe ninu idile yin? *(How many children, in each of the following age groups, live in your household?)*			
1	Odun merin din logun ati ju bee lo *(16+ years)*		Iye omo *(children)*
2	Odun mejila si meedogun *(12-15 years old)*		Iye omo *(children)*
3	Odun mesan si mokonla *(9-11 years old)*		Iye omo *(children)*
4	Odun mefa si mejo *(6-8 years old)*		Iye omo *(children)*
5	Odun meta si marun *(3-5 years old)*		Iye omo *(children)*
6	Odun odo si meji *(0-2 years old)*		Iye omo *(children)*

**S7.** Kini ipo igbeyawo yin? *(What is your marital status?)*		
1	Abileko/ ajumo gbe po pelu enikeji mi *(Married / living together with partner)*	
2	Opo *(Widowed)*	
3	Ati pinya *(Divorced)*	
4	Ipo miran, jowo so *(Other, please specify):* ___________________________________	

**S8a.** I we kika: Nje e ni? *(Education: Do you have?)*		
1	Ko pari ile iwe alakobere *(Primary Incomplete)*	
2	O pari ile iwe alakobere *(Primary complete)*	
3	Ko pari ile iwe girama *(Secondary Incomplete)*	
4	O pari ile iwe girama *(Secondary Complete)*	
5	Ile iwe giga Diploma tabiPoli tabi Collegi: IPELE KINNI *(Polytechnic or College of Education: OND or NCE)*	
6	Ile iwe giga University/Poli, ipele keji *(University/Polytechnic: HND)*	
7	Ko pari ipele ile iwe ti o tele Unifasiti *(Post-University Incomplete)*	
8	O pari ile iwe ti o tele Unifasiti *(Post University Complete)*	
9	Ko le ko tabi ka/rara *(Can't read or write/None)*	

**S8b.** Kilaasi wo ni omo yin obinrin wa (omo obinrin yin ti o nkopa ninu ise iwadi yi)?<br>*(In which class is your daughter(your daughter involved in this study)?)*		
1		

**S9.** Ewo ninu gbogbo akojo wonyi lo se apejuwe gbogbo owo to nwo le si idile yin losoosu?<br>*(Which of the following categories best describes the total net monthly income of your household?)*		
1	O kere si egberun mewa Naira *(Less than N10,000)*	
2	Laarin egberun mewa si ogun egberun Naira *(Between N 10,000 – N 20,000)*	
3	Laarin ogun egberun si aadota egberun Naira *(Between N 20,000 – N 50,000)*	
4	Laarin aadota egberun si ogorun egberun Naira *(Between N 50,000 – N 100,000)*	
5	Ju ogorun egberun Naira lo *(More than N 100,000)*	

SECTION 2: GENERAL QUESTIONS RELATED TO SHOPPING AND COOKING

[INTERVIEWER, PLEASE READ OUT:}

"Ni abala yi, a o nife lati beere lowo yin die si nipa awon ohun ti o wa larowoto yin fun ina dida."

("IN THIS SECTION, WE WOULD LIKE TO ASK YOU A BIT MORE ABOUT WHAT YOU HAVE AVAILABLE IN YOUR HOME.")

[INTERVIEWER: THE DAUGHTER DOES NOT HAVE TO ANSWER THE QUESTION IN SECTION 2]

**2_C1.** Bi eniyan melo ni e ma nsaba dana fun? *(How many people do you usually cook for?)*	

[INTERVIEWER, PLEASE READ OUT:]

Nisinyi ma beere awon ibere nipa ilegbe yin. Ngo ka awon oro kan jade, e jowo e je ki nmo eyi ti o ba je mo yin.

("I AM NOW GOING TO ASK YOU A NUMBER OF QUESTIONS ABOUT YOUR LIVING CIRCUMSTANCES. I WILL READ OUT A NUMBER OF STATEMENTS, PLEASE LET ME KNOW IF THEY APPLY TO YOU.").

[INTERVIEWER, PLEASE INDICATE WHICH OF THE FOLLOWING ITEMS IS AVAILABLE IN THE HOUSEHOLD. IF AN ITEM IS AVAILABLE, BUT IS NOT FUNCTIONAL (I.E., IS BROKEN/NEEDS TO BE FIXED), PLEASE DO NOT SCORE. MULTIPLE OPTIONS POSSIBLE]

**2_C2.** Nje e ni? *(Do you have?)*		
1	Omo odo *(Household help)*	
2	Ero amu omitutu/ero ti o nso nkan di buloku *(Fridge/deep freezer)*	
3	Ero fideo ti a fi nwo aworan *(Video player)*	
4	Oko ayokele *(Car)*	
5	Ero Amohunmaworan oni colour *(Colour TV)*	
6	Ero amorin dun gbamu *(Music system)*	
7	Ero amule tutu *(Air conditioning unit)*	
8	Ero alatagba ayelujara *(Satellite dish)*	
9	Ero ifoso ti o nlo ina eletiriki *(Washing machine)*	
10	Ero amohunmaworan dudu ati funfun *(Black & White TV)*	
11	Ero ta nfi wo awo pelebe *(DVD (Digital video disk)*	
12	Kable satelliti *(Cable satellite)*	
13	Ero ibanisoro (ori ile) *(Telephone (land))*	
14	Ero ibanisoro (alagbeka) *(Telephone (mobile))*	
15	Ero ibanisoro alagbeka ti o nse orisirisi ise *(Smart phone)*	
16	Awako adani *(Personal driver)*	
17	Opolopo Oko ayokele *(Multiple cars)*	
18	Ero ayara bi asa ori tabili *(Computer desktop)*	
19	Ero ayara bia asa alagbeka *(Computer Laptop)*	
20	Ohun imulewo ayarabiasa ti o nsise komputa *(Handheld device/ Ipad)*	

Idana *(Cooking)*

**2_C3.** Nje e ni? *(Do you have?)*

1	Ohun idana ti o nlo afefe gasi *(Gas Cooker)*	
2	Ohun idana ti o nlo ina eletiriki *(Electric Cooker)*	
3	Ohun idana ti o nlo afefe gasi ati ina eletiriki *(Combination Gas & Electrical Stove)*	
4	Ohun idana sitofu ti o nlo epo eebo *(Kerosene stove)*	
5	Ohun idana eedu/igi *(Charcoal/wood)*	

Koko orisun omi *(Main water source)*

**2_C4.** Kini awon koko orisun omi yin? *(What are your main water sources?)*		
1	Omi ero ninu ile *(Inside pipe borne tap)*	
2	Omi ero ni ita *(Outside pipe borne tap)*	
3	Kanga igbalode oni na *(Borehole that works with electicity)*	
4	Kanga igbalode ala fo wo wa *(hand pumped borehole)*	
5	Omi ri ra *(bought water)*	
6	Kanga *(Well)*	
7	Omi odo ti o nsan *(Stream)*	

Iru ile igbonse *(Toilet type)*

**2_C5.** Nje e ni? *(Do you have?)*		
1	Igbonse alawo igbalode ti o wa ninu ile *(Inside WC)*	
2	Igbonse alawo igbalode ti o wa ni ita *(Outside WC)*	
3	Salanga *(Pit latrine)*	
4	Ko si rara *(None)*	

2_C6. Bawo ni e se lagbara to lati ra awon ohun elo wonyi?
Ni osunwon 'mi o le ra rara'si 'mo le ra daadaa', bawo lose lagbara lati ra abi ko le ra awon eroja yi, pelu wiwo osunwo owo idile yin? E le lo eyikeyi anfani yi fun idahun yin.
(On a scale from 'very unaffordable' to 'very affordable', how unaffordable or affordable are the following ingredients for you, considering your household budget? You can use the following options for your answer).

[INTERVIEWER, READ OUT THE OPTIONS]:

Mi o le ra rara *(Very unaffordable)*	Mi o le ra *(Unaffordable)*	Mo le ra, mi o si le ra *(Neither affordable, nor unaffordable)*	Mo le ra *(Affordable)*	Mo le ra daadaa *(Very affordable)*

2_C6	Bawo lose lagbara fun yin lati ra abi ko le ra.. *(How affordable or unaffordable is it for you to get)* [INTERVIEWER READ OPTIONS BELOW AND READ OUT OPTIONS FOR ANSWERS ON THE LEFT]	Mi o le ra rara *(Very unaffordable)* 1	Mi o le ra *(Unaffordable)* 2	Mo le ra, mi o si le ra *(Neither affordable, nor unaffordable)* 3	Mo le ra *(Affordable)* 4	Mo le ra daadaa *(Very affordable)* 5
1	Eran *(Meat)*					
2	Eja *(Fish)*					
3	Ewebe (bii efo, ugu, soko, tete, ewuro) *(Green leafy vegetables (e.g., spinach, ugwu, afam, oha, soko, bitter leaf etc.)*					
4	Ewebe onikoro ti a sese ja (bii ila) *(Fresh green vegetables (e.g., okra)*					
5	Tomati *(Tomatoes)*					
6	Tatase *(Red bell peppers)*					
7	Eso ti a sese ka *(Fresh fruits)*					
8	Alubosa *(Onion)*					
9	Eroja olorun didun *(Spices (e.g. curry, thyme, white pepper, ginger, garlic etc)*					
10	Isebe onikoro Knorr *(Knorr Bouillon cubes)*					
11	Isebe onikoro omiran *(Other bouillon cubes)*					

SECTION 3: FOOD INTAKE QUESTIONNAIRE

[INTERVIEWER, PLEASE READ OUT:]

"Ninu awon ibeere ti o kan, a fe lati mo si nipa bawo ni e se se obe si larin ose meji seyin, awon eroja ti e saba ma nlo lati se won ounje wonyi ati odiwon awon eroja ti e lo".

("IN THE NEXT QUESTIONS, WE WANT TO KNOW MORE ABOUT HOW OFTEN YOU HAVE COOKED STEWS AND SOUPS DURING THE PAST TWO WEEKS, WHICH INGREDIENTS YOU USUALLY USE TO MAKE THESE DISHES AND HOW MUCH OF THESE INGREDIENTS YOU USE").

A. OBE ATA (STEWS)

3_A1. Laarin ose meji to koja, bii emelo ni e ti se ninu awon obe wonyi?
(During the past two weeks, how often have you cooked any of the following stews?)

	**Obe ata eleran malu** *(Beef stew)*	**Obe ata eleja** *(Fish stew)*	**Obe ata ogufe** *(Goat stew)*	**Obe ata didin** *(Fried stew)*	**Obe ata adie/tolotolo** *(Chicken/Turkey)*	**Obe ata miran** *(Other)*
INDICATE NUMBER OF TIMES DURING PAST 2 WEEKS						

3_A2A. OBE ATA EL ERAN MALU (BEEF STEW)

3_A2A. Ti e ba n se koko obe ata eran malu
- Ewo ninu awon eroja ti a ka sile yi ni e fi kun?
- Bawo ni eroja ti e fi kun ti po to?

When cooking a pot of beef stew
- *Which of the following ingredients did you add?*
- *How much of each ingredient did you add?*

ORUKO EROJA (NAME OF INGREDIENT)		MAAKI INU APOTI YI TI O BA FI SI (TICK BOX IF ADDED)	SO IYE OSUWON TI O FI SI NU KOKO OBE (INDICATE NUMBER OF UNITS PER POT)	OSUWON (UNIT) CIRCLE OR UNDERLINE APPROPRIATE ANSWER	
Eran malu	*(Beef)*			Naira	
Alubosa	*(Onions)*			(Kekere/Odinwon/ Nla)	*(Small/medium/large)*
Tatase	*(Red bell pepper)*			(Kobiowu/lanbebe kekere/lanbebe nla)	*(Selling bowl large/ medium/small)*
Ata rodo	*(Spicy red pepper)*			(Kobiowu/lanbebe kekere/lanbebe nla)	*(Selling bowl large/ medium/small)*
Tomati	*(Tomatoes)*			(Kobiowu/lanbebe kekere/lanbebe nla)	*(Selling bowl large/ medium/small)*
Isebe Knorr onikoro	*(Knorr cubes)*			Iye koro	*(Cubes)*
Isebe onikoro miran	*(Other Cubes)*			Iye koro	*(Cubes)*
Ewebe (ugwu, soko, tete, ewe ege, ewe koko, tatse)	*(Green leafy vegetables (ugwu, spinach, amaranth leaves,*			Iye idi	*(Bunches)*
Omiran: …………………..	*(Other:……………….)*				
Omiran: …………………..	*(Other: ……………..)*				

3_A2B. OBE ATA EL EJA (FISH STEW)					
**3_A2B.** Ti e ba n se koko obe ata el eja<br>- Ewo ninu awon eroja ti a ka sile yi ni e fi kun?<br>- Bawo ni eroja ti e fi kun ti po to?			*When cooking a pot of fish stew*<br>- *Which of the following ingredients did you add?*<br>- *How much of each ingredient did you add?*		
ORUKO EROJA (NAME OF INGREDIENT)		MAAKI INU APOTI YI TI O BA FI SI (TICK BOX IF ADDED)	SO IYE OSUWON TI O FI SI NU KOKO OBE (INDICATE NUMBER OF UNITS PER POT)	OSUWON (UNIT) CIRCLE OR UNDERLINE APPROPRIATE ANSWER	
Eja	*(Fish)*			Naira	
Alubosa	*(Onions)*			(Kekere/Odinwon/ Nla)	*(Small/medium/large)*
Tatase	*(Red bell pepper)*			(Kobiowu/lanbebe kekere/lanbebe nla)	*(Selling bowl large/ medium/small)*
Ata rodo	*(Spicy red pepper)*			(Kobiowu/lanbebe kekere/lanbebe nla)	*(Selling bowl large/ medium/small)*
Tomati	*(Tomatoes)*			(Kobiowu/lanbebe kekere/lanbebe nla)	*(Selling bowl large/ medium/small)*
Isebe Knorr onikoro	*(Knorr cubes)*			Iye koro	*(Cubes)*
Isebe onikoro miran	*(Other Cubes)*			Iye koro	*(Cubes)*
Ewebe (ugwu, soko, tete, ewe ege, ewe koko, tatse)	*(Green leafy vegetables (ugwu, spinach, amaranth leaves,*			Iye idi	*(Bunches)*
Omiran: …………………..	*(Other:……………….)*				
Omiran: …………………..	*(Other: ……………..)*				

3_A2C. OBE ATA OGUFE (GOAT STEW)					
**3_A2C**. Ti e ba n se koko obe ata ogufe<br>- Ewo ninu awon eroja ti a ka sile yi ni e fi kun?<br>- Bawo ni eroja ti e fi kun ti po to?			*When cooking a pot of goat stew*<br>- *Which of the following ingredients did you add?*<br>- *How much of each ingredient did you add?*		
ORUKO EROJA (NAME OF INGREDIENT)		MAAKI INU APOTI YI TI O BA FI SI (TICK BOX IF ADDED)	SO IYE OSUWON TI O FI SI NU KOKO OBE (INDICATE NUMBER OF UNITS PER POT)	OSUWON (UNIT) CIRCLE OR UNDERLINE APPROPRIATE ANSWER	
Eran ogufe	*(Goat Meat)*			Naira	
Alubosa	*(Onions)*			(Kekere/Odinwon/ Nla)	*(Small/medium/large)*
Tatase	*(Red bell pepper)*			(Kobiowu/lanbebe kekere/lanbebe nla)	*(Selling bowl large/ medium/small)*
Ata rodo	*(Spicy red pepper)*			(Kobiowu/lanbebe kekere/lanbebe nla)	*(Selling bowl large/ medium/small)*
Tomati	*(Tomatoes)*			(Kobiowu/lanbebe kekere/lanbebe nla)	*(Selling bowl large/ medium/small)*
Isebe Knorr onikoro	*(Knorr cubes)*			Iye koro	*(Cubes)*
Isebe onikoro miran	*(Other Cubes)*			Iye koro	*(Cubes)*
Ewebe (ugwu, soko, tete, ewe ege, ewe koko, tatse)	*(Green leafy vegetables (ugwu, spinach, amaranth leaves,*			Iye idi	*(Bunches)*
Omiran: …………………..	*(Other:……………….)*				
Omiran: …………………..	*(Other: ……………..)*				

3_A2D. OBE ATA DINDIN (FRIED STEW)					
**3_A2D.** Ti e ba n se koko obe ata dindin<br>- Ewo ninu awon eroja ti a ka sile yi ni e fi kun?<br>- Bawo ni eroja ti e fi kun ti po to?			*When cooking a pot of fried stew*<br>- *Which of the following ingredients did you add?*<br>- *How much of each ingredient did you add?*		
ORUKO EROJA (NAME OF INGREDIENT)		MAAKI INU APOTI YI TI O BA FI SI (TICK BOX IF ADDED)	SO IYE OSUWON TI O FI SI NU KOKO OBE (INDICATE NUMBER OF UNITS PER POT)	OSUWON (UNIT) CIRCLE OR UNDERLINE APPROPRIATE ANSWER	
Alubosa	*(Onions)*			(Kekere/Odinwon/ Nla)	*(Small/medium/large)*
Tatase	*(Red bell pepper)*			(Kobiowu/lanbebe kekere/lanbebe nla)	*(Selling bowl large/ medium/small)*
Ata rodo	*(Spicy red pepper)*			(Kobiowu/lanbebe kekere/lanbebe nla)	*(Selling bowl large/ medium/small)*
Tomati	*(Tomatoes)*			(Kobiowu/lanbebe kekere/lanbebe nla)	*(Selling bowl large/ medium/small)*
Isebe Knorr onikoro	*(Knorr cubes)*			Iye koro	*(Cubes)*
Isebe onikoro miran	*(Other Cubes)*			Iye koro	*(Cubes)*
Ewebe (ugwu, soko, tete, ewe ege, ewe koko, tatse)	*(Green leafy vegetables (ugwu, spinach, amaranth leaves,*			Iye idi	*(Bunches)*
Omiran: ………………….	*(Other:……………….)*				
Omiran: ………………….	*(Other: ……………..)*				

3_A2E. OBE ATA ADIE/TOLOTOLO (CHICKEN/TURKEY)					
**3_A2E.** Ti e ba n se koko obe ata adie/tolotolo<br>- Ewo ninu awon eroja ti a ka sile yi ni e fi kun?<br>- Bawo ni eroja ti e fi kun ti po to?			*When cooking a pot of chicken/turkey stew*<br>- *Which of the following ingredients did you add?*<br>- *How much of each ingredient did you add?*		
ORUKO EROJA (NAME OF INGREDIENT)		MAAKI INU APOTI YI TI O BA FI SI (TICK BOX IF ADDED)	SO IYE OSUWON TI O FI SI NU KOKO OBE (INDICATE NUMBER OF UNITS PER POT)	OSUWON (UNIT) CIRCLE OR UNDERLINE APPROPRIATE ANSWER	
Eran adiye	*(Chicken Meat)*			Naira	
Eran tolotolo	*(Turkey Meat)*			Naira	
Alubosa	*(Onions)*			(Kekere/Odinwon/ Nla)	*(Small/medium/large)*
Tatase	*(Red bell pepper)*			(Kobiowu/lanbebe kekere/lanbebe nla)	*(Selling bowl large/ medium/small)*
Ata rodo	*(Spicy red pepper)*			(Kobiowu/lanbebe kekere/lanbebe nla)	*(Selling bowl large/ medium/small)*
Tomati	*(Tomatoes)*			(Kobiowu/lanbebe kekere/lanbebe nla)	*(Selling bowl large/ medium/small)*
Isebe Knorr onikoro	*(Knorr cubes)*			Iye koro	*(Cubes)*
Isebe onikoro miran	*(Other Cubes)*			Iye koro	*(Cubes)*
Ewebe (ugwu, soko, tete, ewe ege, ewe koko, tatse)	*(Green leafy vegetables (ugwu, spinach, amaranth leaves,*			Iye idi	*(Bunches)*
Omiran: ………………….	*(Other:……………….)*				
Omiran: ………………….	*(Other: ……………..)*				

3_A2F. OBE ATA MIRAN: ______________________________ (OTHER STEW______________________)					
**3_A2F.** Ti e ba n se koko obe ata<br>- Ewo ninu awon eroja ti a ka sile yi ni e fi kun?<br>- Bawo ni eroja ti e fi kun ti po to?			*When cooking a pot of stew*<br>- *Which of the following ingredients did you add?*<br>- *How much of each ingredient did you add?*		
ORUKO EROJA (NAME OF INGREDIENT)		MAAKI SI INU APOTI YI TI O BA FI SI (TICK BOX IF ADDED)	SO IYE OSUWON TI O FI SI NU KOKO OBE (INDICATE NUMBER OF UNITS PER POT)	OSUWON (UNIT) CIRCLE OR UNDERLINE APPROPRIATE ANSWER	
Eran	*(Meat)*			Naira	
Alubosa	*(Onions)*			(Kekere/Odinwon/ Nla)	*(Small/medium/large)*
Tatase	*(Red bell pepper)*			(Kobiowu/lanbebe kekere/lanbebe nla)	*(Selling bowl large/ medium/small)*
Ata rodo	*(Spicy red pepper)*			(Kobiowu/lanbebe kekere/lanbebe nla)	*(Selling bowl large/ medium/small)*
Tomati	*(Tomatoes)*			(Kobiowu/lanbebe kekere/lanbebe nla)	*(Selling bowl large/ medium/small)*
Isebe Knorr onikoro	*(Knorr cubes)*			Iye koro	*(Cubes)*
Isebe onikoro miran	*(Other Cubes)*			Iye koro	*(Cubes)*
Ewebe (ugwu, soko, tete, ewe ege, ewe koko, tatse)	*(Green leafy vegetables (ugwu, spinach, amaranth leaves,*			Iye idi	*(Bunches)*
Omiran: …………………..	*(Other:…………….)*				
Omiran: …………………..	*(Other: …………….)*				

B. OBE (Soups)

[Interviewer, read out different options under 'How Often' for each of the options]

**3_B1.** Laarin ose meji ti o koja, bii emelo ni e se awon obe ti o wa nisale yi? *(During the past two weeks, how often have you cooked the following soups?)*					
	**Egusi** *(Egusi soup)*	**Efo riro** *(Spinach/Amaranth soup)*	**Ila** *(Okra soup)*	**Ewedu** *(Ewedu soup)*	**Gbegiri** *(Bean soup)*
Indicate number of times during past 2 weeks					

3_B2A. Egusi					
**3_B2A.** Ti e ba n se obe egusi<br>- Ewo ninu awon eroja ti a ka sile yi ni e fi kun?<br>- Bawo ni eroja ti e fi kun tip o to?			*When cooking egusi soup*<br>- *Which of the following ingredients did you add?*<br>- *How much of each ingredient did you add?*		
**Oruko eroja (Name of ingredient)**		**Maaki inu apoti yi ti o ba fi si (Tick box if added)**	**So iye osuwon ti o fi si nu koko obe (Indicate number of units per pot)**	**Osuwon (Unit) Circle or underline appropriate answer**	
Eran	*(Meat)*			Naira	
Alubosa	*(Onions)*			(Kekere/Odinwon/ Nla)	*(Small/medium/large)*
Tatase	*(Red bell pepper)*			(Kobiowu/lanbebe kekere/lanbebe nla)	*(Selling bowl large/ medium/small)*
Tomati	*(Tomatoes)*			(Kobiowu/lanbebe kekere/lanbebe nla)	*(Selling bowl large/ medium/small)*
Egusi	*(melon seeds)*			(Agolo miliki)	*(Milk tin)*
Isebe Knorr onikoro	*(Knorr cubes)*			(Iye koro)	*(Cubes)*
Isebe Maggi onikoro	*(Other Cubes)*			(Iye koro)	*(Cubes)*
Ewebe (ugu, soko, tete, ewe ege, ewe koko, tatse)	*(Green leafy vegetables (ugwu, spinach, amaranth leaves, cassava leaves, cocoyam leaves)*			(Iye idi)	*(Bunches)*
Omiran: …………………..	*(Other:………………….)*				
Omiran: …………………..	*(Other: ……………….)*				

3_B2B. EFO RIRO (SPINACH/AMARANTH)					
**3_B2B.** Ti e ba n se efo riro<br>- Ewo ninu awon eroja ti a ka sile yi ni e fi kun?<br>- Bawo ni eroja ti e fi kun tip o to?			*When cooking spinach/amaranth soup*<br>- *Which of the following ingredients did you add?*<br>- *How much of each ingredient did you add?*		
**ORUKO EROJA (NAME OF INGREDIENT)**		**MAAKI INU APOTI YI TI O BA FI SI (TICK BOX IF ADDED)**	**SO EYE OSUWON TI O FI SI NU KOKO OBE (INDICATE NUMBER OF UNITS PER POT)**	**OSUWON (UNIT) CIRCLE OR UNDERLINE APPROPRIATE ANSWER**	
Eran	*(Meat)*			Naira	
Alubosa	*(Onions)*			(Kekere/Odinwon/ Nla)	*(Small/medium/large)*
Tatase	*(Red bell pepper)*			(Kobiowu/lanbebe kekere/lanbebe nla)	*(Selling bowl large/ medium/small)*
Tomati	*(Tomatoes)*			(Kobiowu/lanbebe kekere/lanbebe nla)	*(Selling bowl large/ medium/small)*
Isebe Knorr onikoro	*(Knorr cubes)*			(Iye koro)	*(Cubes)*
Isebe Maggi onikoro	*(Other Cubes)*			(Iye koro)	*(Cubes)*
Ewebe (ugu, soko, tete, ewe ege, ewe koko, tatse)	*(Green leafy vegetables (ugwu, spinach, amaranth leaves, cassava leaves, cocoyam leaves)*			(Iye idi)	*(Bunches)*
Omiran: …………………..	*(Other:……………….)*				
Omiran: …………………..	*(Other: ……………..)*				

3_B2C. OBE ILA (OKRA SOUP)					
**3_B2C.** Ti e ba n se obe ila<br>- Ewo ninu awon eroja ti a ka sile yi ni e fi kun?<br>- Bawo ni eroja ti e fi kun tip o to?			*When cooking okra soup*<br>- *Which of the following ingredients did you add?*<br>- *How much of each ingredient did you add?*		
**ORUKO EROJA (NAME OF INGREDIENT)**		**MAAKI INU APOTI YI TI O BA FI SI (TICK BOX IF ADDED)**	**SO EYE OSUWON TI O FI SI NU KOKO OBE (INDICATE NUMBER OF UNITS PER POT)**	**OSUWON (UNIT) CIRCLE OR UNDERLINE APPROPRIATE ANSWER**	
Eran	*(Meat)*			Naira	
Alubosa	*(Onions)*			(Kekere/Odinwon/ Nla)	*(Small/medium/large)*
Tatase	*(Red bell pepper)*			(Kobiowu/lanbebe kekere/lanbebe nla)	*(Selling bowl large/ medium/small)*
Tomati	*(Tomatoes)*			(Kobiowu/lanbebe kekere/lanbebe nla)	*(Selling bowl large/ medium/small)*
Egusi	*(melon seeds)*			(Agolo miliki)	*(Milk tin)*
Ila	*(Okra)*			(Lanbebe kekere/lanbebe nla)	*(Selling bowl medium/small)*
Isebe Knorr onikoro	*(Knorr cubes)*			(Iye koro)	*(Cubes)*
Isebe Maggi onikoro	*(Other Cubes)*			(Iye koro)	*(Cubes)*
Ewebe (ugu, soko, tete, ewe ege, ewe koko, tatse)	*(Green leafy vegetables (ugwu, spinach, amaranth leaves, cassava leaves, cocoyam leaves)*			(Iye idi)	*(Bunches)*
Omiran: …………………..	*(Other:……………….)*				
Omiran: …………………..	*(Other: ……………..)*				

3_B2D. EWEDU				
**3_B2D.** Ti e ba n se obe ewedu<br>- Ewo ninu awon eroja ti a ka sile yi ni e fi kun?<br>- Bawo ni eroja ti e fi kun tip o to?			*When cooking ewedu soup*<br>- *Which of the following ingredients did you add?*<br>- *How much of each ingredient did you add?*	
**ORUKO EROJA (NAME OF INGREDIENT)**	**MAAKI INU APOTI YI TI O BA FI SI (TICK BOX IF ADDED)**	**SO EYE OSUWON TI O FI SI NU KOKO OBE (INDICATE NUMBER OF UNITS PER POT)**	**OSUWON (UNIT) CIRCLE OR UNDERLINE APPROPRIATE ANSWER**	
Eran *(Meat)*			Naira	
Alubosa *(Onions)*			(Kekere/Odinwon/ Nla)	*(Small/medium/large)*
Tatase *(Red bell pepper)*			(Kobiowu/lanbebe kekere/lanbebe nla)	*(Selling bowl large/ medium/small)*
Tomati *(Tomatoes)*			(Kobiowu/lanbebe kekere/lanbebe nla)	*(Selling bowl large/ medium/small)*
Egusi *(melon seeds)*			(Agolo miliki)	*(Milk tin)*
Isebe Knorr onikoro *(Knorr cubes)*			(Iye koro)	*(Cubes)*
Isebe Maggi onikoro *(Other Cubes)*			(Iye koro)	*(Cubes)*
Ewebe (ugu, soko, tete, ewe ege, ewe koko, tatse) *(Green leafy vegetables (ugwu, spinach, amaranth leaves, cassava leaves, cocoyam leaves)*			(Iye idi)	*(Bunches)*
Omiran: .... *(Other:……………….)*				
Omiran: .... *(Other: ……………..)*				

3_B2E. GBEGIRI (BEAN SOUP)				
**3_B2E.** Ti e ba n se obe gbegiri<br>- Ewo ninu awon eroja ti a ka sile yi ni e fi kun?<br>- Bawo ni eroja ti e fi kun tip o to?			*When cooking bean soup*<br>- *Which of the following ingredients did you add?*<br>- *How much of each ingredient did you add?*	
**ORUKO EROJA (NAME OF INGREDIENT)**	**MAAKI INU APOTI YI TI O BA FI SI (TICK BOX IF ADDED)**	**SO EYE OSUWON TI O FI SI NU KOKO OBE (INDICATE NUMBER OF UNITS PER POT)**	**OSUWON (UNIT) CIRCLE OR UNDERLINE APPROPRIATE ANSWER**	
Eran *(Meat)*			Naira	
Alubosa *(Onions)*			(Kekere/Odinwon/ Nla)	*(Small/medium/large)*
Tatase *(Red bell pepper)*			(Kobiowu/lanbebe kekere/lanbebe nla)	*(Selling bowl large/ medium/small)*
Tomati *(Tomatoes)*			(Kobiowu/lanbebe kekere/lanbebe nla)	*(Selling bowl large/ medium/small)*
Ewa *(beans)*			(Agolo miliki)	*(Milk tin)*
Isebe Knorr onikoro *(Knorr cubes)*			(Iye koro)	*(Cubes)*
Isebe Maggi onikoro *(Other Cubes)*			(Iye koro)	*(Cubes)*
Ewebe (ugu, soko, tete, ewe ege, ewe koko, tatse) *(Green leafy vegetables (ugwu, spinach, amaranth leaves, cassava leaves, cocoyam leaves)*			(Iye idi)	*(Bunches)*
Omiran: .... *(Other:……………….)*				
Omiran: .... *(Other: ……………..)*				

**3_C.** Bawo le se se irufe awon ounje yii si ninu ose meji to koja?<br>*(How often have you prepared any of the following dishes during the past two weeks?)*									
	Iresi fufun sise *(Boiled rice)*	Iresi eleroja ata *(Jollof rice)*	Abo efo ni egbe kan *(side dish of green leafy vegetables)*	Iresi dindin *(fried rice)*	Ewa sisi *(Cooked Beans)*	Omiran ________ *(Other)*	Omiran ________ *(Other)*	Omiran ________ *(Other)*	Omiran ________ *(Other)*
INDICATE NUMBER OF TIMES DURING PAST 2 WEEKS									

SECTION 4: KNOWLEDGE/AWARENESS QUESTIONS ON IRON DEFICIENCY

[INTERVIEWER, PLEASE READ OUT:]

"Ninu awon ibeere ti o kan, a nife lati ni oye ati owon ohun ti e mo nipa aito eroja iron (aito eje), awon ami ati ohun ti o le ti eyin re yo. Ma fe fi idi re mule wipe eleyi kii se idanwo, mo nifesi idahun yin ni otito".

("IN THE NEXT FEW QUESTIONS, WE WOULD LIKE TO UNDERSTAND HOW MUCH YOU KNOW ABOUT IRON DEFICIENCY (ANAEMIA), ITS SYMPTOMS AND ITS CONSEQUENCES. I WOULD LIKE TO EMPHASISE THAT THIS IS NOT A TEST, I AM INTERESTED IN YOUR HONEST ANSWER.")

K1. Bawo ni e se ma n ri awon apere yi si?
(How often do you experience the following symptoms?)

	O din ni ekan losu tabi rara *(Less than once a month or never)*	Ekan si emeta losu *(1 – 3 times a month)*	Ekan lose *(Once a week)*	Emeta si emarun lose *(3-5 times a week)*	Ekan lojumo *(Once a day)*	Ju ekan lo lojumo *(More than once a day)*
Aile fi okan si ibi nkan daadaa *(Poor concentration)*						
Rire ni bi o ti le je pe a sun daa daa *(Tiredness, even though you have had enough sleep)*						
Maa kanra tabi binu lai si ohun kan ti o gbodi *(Increased irritability, when nothing has really gone wrong)*						
Oyi kikoni *(Dizziness)*						
Sise funfun awo ara *(Paler complexion)*						

K2. Nje e ti gbo nipa aisan kan ti a n pe ni "aisan aito eje lara"?
*(Have you ever heard of a condition called "**anaemia**"/"iron deficiency"? (note: translated as "insufficient blood")*

1	Beeni *(Yes)*		CONTINUE WITH K3
2	Beeko *(No)*		CONTINUE WITH K5

[READ THE NEXT QUESTION AND READ OUT THE OPTIONS. **TICK IF RESPONSE IS "YES". DO NOT TICK IF RESPONDENT INDICATES "NO" OR "DON'T KNOW". MULTIPLE CODING POSSIBLE.]**

**K3.** Ewo ninu awon ipo yi ni o le je ki eniyan ni aito eje? *(According to you, which of these conditions/situations might increase the risk of anaemia?)*			
1	Idile ti o ni itan aito eje	*(Family history of anaemia)*	
2	Kikere ju bi o se ye lo	*(Being underweight)*	
3	Titobi ju bi o se ye lo	*(Being overweight/obese)*	
4	Ikun ti o ba tobi	*(Having a large waist/ a tummy)*	
5	Ounje ti o ni ora pupo	*(High fat diet)*	
6	Idaamu/ aifokanbale/inira	*(Stress/ tension)*	
7	Jije suga ati ohun ti o dun ju	*(Excessive intake of sweets/ sugar)*	
8	Ai maa se ere idaraya	*(Lack of physical activity)*	
9	Ai ma je ewebe ti a sese ja bi ugu, efo, soko, tete, ewe ege, ewe koko to	*(Low intake of green leafy vegetables such as ugwu, spinach, cassava leaves, cocoyam leaves)*	
10	Ai ma je ewebe alata (tomati, ata rodo,) to	*(Low intake of red/orange vegetables (e.g., red pepper, tomato)*	
11	Ai ma je ewebe onikoro bii ewa elewe to	*(Low intake of green vegetables such as green beans, okra)*	
12	Ai maa je ewebe alawo aluko, fufun (bii irugbin igba, ege) to	*(Low intake of purple, white vegetables (e.g., garden egg, cassava)*	
13	Ai maa je eso to	*(Low intake of fruits)*	
14	Ai maa je eran pupa (maalu, ewure) to	*(Low intake of red meat (beef, goat)*	
15	Ai maa je eja to	*(Low intake of fish)*	
16	Ai maa je eyin to	*(Low intake of eggs)*	
17	Ai maa je ewa to	*(Low intake of beans)*	
18	Ai maa sun to	*(Insufficient sleep)*	
19	Siga fifa	*(Smoking)*	
20	Liloyun	*(Being pregnant)*	
21	Mi ma se nkan osu	*(Having your period)*	
23	Ailera ara	*(Being sick)*	
24	Nini aran	*(Having worms)*	
25	Awon ohun miran (e so) ____	*(Others (Specify)* ________________________________	

[READ THE QUESTION AND READ OUT THE OPTIONS. TICK RESPONSES PROVIDED BY RESPONDENT. **MULTIPLE CODING POSSIBLE**]

**K4.** Ewo ninu awon wonyi ni ona abayo ti o muna doko fun aito eje lara? *(Which of these are effective solutions for "anaemia" or "iron deficiency"?)*					UNAIDED
			**Beeni** *Yes (1)*	**Beeko** *No (2)*	**Mi o mo** *(Don't know)*
1	Ki a ma je efo gege bi ara ounje wa	*(Eating vegetables as part of a meal)*			
2	Ki a ma gun, lo, ati mu omi ti a ri lara ewe ugu	*(Crushing, grinding and drinking the juice from pumpkin leaves)*			
3	Mimu egbogi ibile orisirisi ti a sepo	*(Drinking herbal mixes)*			
4	Jije ogede ti ko pon	*(Eating unripe plantains)*			
5	Jije edo	*(Consuming liver)*			
6	Lilo "ogun eje olomi" tabi "ogun eje onikoro'	*(Consuming 'blood tonic' or 'blood tablets)*			
7	Ki a ma je ewebe ti a sese ja bi ugu, efo, tete, soko	*(Consuming green leafy vegetables, such as ugwu, spinach, amaranth leaves)*			
8	Ki a ma je ewebe pupa bi ata rodo, tatase, timati elegede	*(Consuming red/orange vegetables such as red pepper, tomato, pumpkins)*			
9	Jije eso	*(Consuming fruits)*			
10	Jije eran ju ti ateyin wa lo (bii, maalu, ewure)	*(Consuming more meats (e.g., beef, goat)*			
11	Jije eyin ju ti ateyin wa lo	*(Consuming more eggs)*			
12	Jije eja ju ti ateyin wa lo	*(Consuming more fish)*			
13	Jije ewa ju ti ateyin wa lo	*(Consuming more beans)*			
14	Ki a ma sun ju ti tele lo	*(Getting more sleep)*			
15	Ki a ma se ere idaraya ju ti tele lo	*(Being more physically active)*			
16	Awon ohun miran (e so) ______________________________	*(Others (Specify)* ______________________________			

[READ THE STATEMENTS AND READ OUT THE ANSWER OPTIONS. TICK RESPONSES PROVIDED BY RESPONDENT. IF THE RESPONDENT REALLY DOES NOT KNOW, TICK "DON'T KNOW", BUT DO NOT PROMPT]

K5. E jowo e mu eyi ti o ba so bi e se faramo tabi lodi si awon oro ti a ko si isale wonyi.
(Please indicate for the following statements how much you agree or disagree with the following statements.)

		**Mi o fara mo rara**<br>*Disagree Strongly (1)*	**Mi o faramo die**<br>*Disagree Slightly (2)*	**Mo faramo/Mi o faramo**<br>*Neither agree/Nor disagree (3)*	**Mo fara mo die**<br>*Agree Slightly (4)*	**Mo faramo gan**<br>*Agree Strongly (5)*	**Mi o mo**<br>*I don't know (9)*
1	Eroja iron dara fun ilera<br>*(Iron is good for the health)*						
2	Jije ounje ti o ni eronja eje ninu nse iranlowo lati pese eje<br>*(Eating foods containing iron helps build blood*						
3	Ounje ti o ni eroja eje ninu maa nran awon soja ara lowo<br>*(Foods containing iron strengthens the immune system)*						
4	Eroja eje iron ko ni iwulo kankan fun ilera ara<br>*(Iron has no healthy benefit for the body)*						
5	A ko le je eroja ti o nfun ni ni eje ni apoju<br>*(You cannot consume too much iron)*						
6	Eroja iron nran eje lowo lati gbe ategun kiri ago ara wa<br>*(Iron helps your blood carry oxygen through the body)*						
7	Jije ounje ti o ni eroja eje ma nran omo lowo lati dagba<br>*(Eating iron rich foods helps children develop)*						
8	Jije ounje ti o ni eroja eje ma nse iranlowo lati le fi okan si nkan<br>*(Consuming foods containing iron helps with concentration/focus)*						
9	Jije ounje ti o ni eroja eje maa nse iranlowo lati le ni okun ni ojumo<br>*(Consuming foods containing iron helps one have more energy during the day)*						
10	Jije ounje ti o ni eroja eje se iranlowo lati din oyi kikoni ku<br>*(Consuming foods containing iron can help reduce dizziness)*						

[READ THE QUESTION AND READ OUT THE OPTIONS. TICK RESPONSES PROVIDED BY RESPONDENT. **MULTIPLE CODING POSSIBLE**]

**K6.** Ewo ninu awon ounje wonyi ni o lero pe o kun fun eronja ti o n fun ni ni eje? *(Please select which of the foods described you think are good sources of iron?)*					UNAIDED
			**Beeni** *Yes (1)*	**Beeko** *No (2)*	**Mi o mo** *Don't know*
1	Eyin	*(Eggs)*			
2	Eja	*(Fish)*			
3	Eran pupa	*(Red meat)*			
4	Buredi ati iyefun	*(bread & flour)*			
5	Ewa	*(Beans)*			
6	Koro eso igi (furutu, kaju, awusa, epa ati bee bee lo)	*(Nuts (Ground nuts, Cashews, Peanuts, etc)*			
7	Ewebe alawo eweko (soko, Ugwu, tete, etc)	*(Green vegetables (Spinach, Ugwu, amaranth leaves, etc)*			
8	Koko, chocolati	*(Cocoa, Chocolate)*			
9	Akara oyinbo eleso ti eroja re pe	*(Rich fruit cake)*			
10	Woro irugbin (agbado ti a ti tun se si ounje oyinbo (Oatmeal), iresi, oka baba, ati bee bee lo etc)	*(Grains (Rice, Oatmeal, Millet, Maize, etc)*			
11	Ogede dudu	*(unripe plaintain)*			
12	Miliki	*(milk)*			
13	Oti stout, malti	*(stout, malt)*			

SECTION 5: DETERMINANTS OF BEHAVIOUR QUESTIONNAIRE

INTERVIEWER PLEASE READ OUT:

"Ninu awon ibeere ti o kan a nife lati mo eroungba yin nipa obe sisie. A nife lati mo ero yin ni otito, ko si idahun ti o tona tabi to kuna. jowo so eyi ti idahun yin ba je nipa lilo awon ohun ti a ti ko sile."

("IN THE NEXT FEW QUESTIONS WE ARE INTERESTED IN YOUR OPINION ABOUT COOKING STEWS. WE ARE INTERESTED IN YOUR HONEST OPINION, THERE ARE NO RIGHT OR WRONG ANSWERS. PLEASE INDICATE YOUR ANSWER USING THE OPTIONS BELOW EACH STATEMENT.")

[INTERVIEWER: DO NOT READ OUT THE HEADINGS IN BOLD CAPITALS, ONLY THE QUESTIONS]

KNORR BOUILLON CUBES

5_1. ATTITUDE

**5_1**	Fun emi, lilo koro Knorr nigba ti mo ba n se obe ata maa n je ki obe ata…<br>*For me, adding Knorr Bouillon cubes makes the stew…*				
a	**Ko wu oju ri rara**<br>*Very unpleasant (1)*	**Ko wu oju ri**<br>*Unpleasant (2)*	**Kosiyato**<br>*Not unpleasant, not pleasant (3)*	**O wu oju ri**<br>*Pleasant (4)*	**O wu oju ri daradara**<br>*Very pleasant (5)*
b	**O baje gaan**<br>*Very bad (1)*	**O baje**<br>*Bad (2)*	**Ko baje, ko dara**<br>*Not bad, not good (3)*	**O dara**<br>*Good (4)*	**O dara gan**<br>*Very good (5)*
b	**O fun ni ni ailera gaan**<br>*Very unhealthy (1)*	**O fun ni ni ailera**<br>*Unhealthy (2)*	**Kosiyato**<br>*Not unhealthy, not healthy (3)*	**O fun ni ni ilera**<br>*Healthy (4)*	**O fun ni ni ilera dara dara**<br>*Very healthy (5)*
d	**O te gan**<br>*Not at all tasty (1)*	**O te**<br>*Not so tasty (2)*	**Kosiyato**<br>*Neutral/undecided (3)*	**Ko te**<br>*Tasty (4)*	**Ko te rara**<br>*Very tasty (5)*
e	**Ko ma dun rara**<br>*Not at all delicious (1)*	**Ko ma dun**<br>*Not so delicious (2)*	**Kosiyato**<br>*Neutral/undecided (3)*	**Ko dun**<br>*Delicious (4)*	**Ko dun gan**<br>*Very delicious (5)*
f	**Ko ma ri ni lara rara**<br>*Not at all disgusting (1)*	**Ko ma ri ni lara**<br>*Not so disgusting (2)*	**Kosiyato**<br>*Neutral/undecided (3)*	**Ko ri ni lara**<br>*Disgusting (4)*	**Ko ri ni lara gan**<br>*Very disgusting (5)*

5_2. SOCIAL NORM (INJUNCTIVE/HUSBAND/FATHER)

[DO NOT ASK 5_2 IF MOTHER IS A WIDOW OR DIVORCED]

5_2a	Oko (baba) mi ro pe o ye ki n fi koro Knorr sinu obe ata.<br>*My husband/father thinks I should add Knorr Bouillon cubes to stews.*				
	**Mi o fara mo rara**<br>*Disagree Strongly (1)*	**Mi o faramo**<br>*Disagree (2)*	**Mo faramo/Mi o faramo**<br>*Neither agree/Nor disagree (3)*	**Mo faramo**<br>*Agree (4)*	**Mo faramo gan**<br>*Agree Strongly (5)*
b	Mo ro pe oko (baba) mi ro pe o ye ki n lo koro Knorr nigbatimo ban se obe ata.<br>*I think my husband/father expects me to use Knorr Bouillon cubes when cooking stews.*				
	**Mi o fara mo rara**<br>*Disagree Strongly (1)*	**Mi o faramo**<br>*Disagree (2)*	**Mo faramo/Mi o faramo**<br>*Neither agree/Nor disagree (3)*	**Mo faramo**<br>*Agree (4)*	**Mo faramo gan**<br>*Agree Strongly (5)*
c	Nigba ti n ba fi koro Knorr sinu obe ata mi, oko (baba) mi…<br>*When I add Knorr Bouillon cubes to my stew, my husband/father would.*				
	**Ko ni gba rara**<br>*Disapprove strongly (1)*	**Ko ni gba**<br>*Disapprove (2)*	**O le gba o le ma gba**<br>*Neither approve nor disapprove (3)*	**Yi o gba**<br>*Approve (4)*	**Yi o gba gan**<br>*Approve strongly (5)*

5_3. SOCIAL NORM (INJUNCTIVE)

5_3a	Opolopo awon ti o se Pataki simi ro pe o ye kin fi koro Knorr si obe ata.<br>*Most people who are important to me think I should add Knorr Bouillon cubes to stews.*				
	**Mi o fara mo rara**<br>*Disagree Strongly (1)*	**Mi o faramo**<br>*Disagree (2)*	**Mo Faramo/Mi o faramo**<br>*Neither agree/Nor disagree (3)*	**Mo Faramo**<br>*Agree (4)*	**Mo Faramo gan**<br>*Agree Strongly (5)*
b	Mo ro pe awon toku ro pe o ye ki n lo koro Knorr ti mo ba n se obe ata.<br>*I think others expect me to use Knorr Bouillon cubes when cooking stews.*				
	**Mi o fara mo rara**<br>*Disagree Strongly (1)*	**Mi o faramo**<br>*Disagree (2)*	**Mo Faramo/Mi o faramo**<br>*Neither agree/Nor disagree (3)*	**Mo Faramo**<br>*Agree (4)*	**Mo Faramo gan**<br>*Agree Strongly (5)*
c	Nigbati mo ba fi koro Knorr si inu obe ata mi, awon eniyan ti eroungba won se iyebiye si mi …<br>*When I add Knorr Bouillon cubes to my stew, the people in my life whose opinions I value would…*				
	**Ko ni gba rara**<br>*Disapprove strongly (1)*	**Ko ni gba**<br>*Disapprove (2)*	**Ko ni gba/Gba**<br>*Neither approve nor disapprove (3)*	**Yi o gba**<br>*Approve (4)*	**Yi o gba gan**<br>*Approve strongly (5)*

5_4. *SOCIAL NORM (DEDUCTIVE)*

5_4a	Opolopo awon ti o se Pataki simi ni o ma n fi koro Knorr si obe ata.<br>*Most people who are important to me add Knorr Bouillon Cubes to stews.*				
	**Mi o fara mo rara**<br>*Disagree Strongly (1)*	**Mi o faramo**<br>*Disagree (2)*	**Mo Faramo/Mi o faramo**<br>*Neither agree/Nor disagree (3)*	**Mo Faramo**<br>*Agree (4)*	**Mo Faramo gan**<br>*Agree Strongly (5)*
b	Awon eniyan ti o wa ninu aye mi , ti erongba won se Pataki simi, maa n fi koro Knorr si obe ata won.<br>*The people in my life whose opinion I value, add Knorr Bouillon cubes to their stews.*				
	**Mi o fara mo rara**<br>*Disagree Strongly (1)*	**Mi o faramo**<br>*Disagree (2)*	**Mo Faramo/Mi o faramo**<br>*Neither agree/Nor disagree (3)*	**Mo Faramo**<br>*Agree (4)*	**Mo Faramo gan**<br>*Agree Strongly (5)*
c	Ninu agbegbe yin, eniyan melo ni e le so pe o n fi koro Knorr si obe ata won?<br>*In your community, how many people would you say add Knorr Bouillon cubes to their stews?*				
	**Won kere gan**<br>*Very few 1*	**Won kere**<br>*Quite a few 2*	**Won to ilaji**<br>*About half 3*	**Won po**<br>*A lot 4*	**Yi o fe je gbogbo won**<br>*Almost all 5*

5_5. *PERCEIVED BEHAVIOURAL CONTROL*

5_5a	Fun emi, fifi koro Knorr si gbogbo obe ata ti mo n se …<br>*For me, adding Knorr Bouillon to every stews I cook would be…*				
	**Yi o Soro gan**<br>*Very difficult (1)*	**Yi o Soro**<br>*Difficult (2)*	**Kosoro/kororun**<br>*Not difficult, not easy (3)*	**O Rorun**<br>*Easy (4)*	**O Rorun pupo**<br>*Very easy (5)*
b	Ti o ba wu mi, mo le fi koro Knorr si gbogbo obe ata ti mo ba se.<br>*If I wanted to, I could add Knorr Bouillon cubes to every stew I cook.*				
	**Mi o fara mo rara**<br>*Disagree Strongly (1)*	**Mi o faramo**<br>*Disagree (2)*	**Mo Faramo/Mi o faramo**<br>*Neither agree/Nor disagree (3)*	**Mo Faramo**<br>*Agree (4)*	**Mo Faramo gan**<br>*Agree Strongly (5)*
c	Bawo ni o se wa lowo yin si lati fi koro Knorr si gbogbo obe ata ti e ba se?<br>*To what extent is it up to you to add Knorr Bouillon Cubes to the stews you cook?*				
	**Gbogbo re wa lowo elomiran**<br>*Completely up to others (1)*	**O wa lowo elomiran lopo igba**<br>*Mostly up to others 2*	**O wa laarin elomiran ati emi**<br>*Both up to others and up to me (3)*	**O wa lowo mi lopo igba**<br>*Mostly up to me (4)*	**O wa lowo emi nikan soso**<br>*Completely up to me (5)*

5_6. *HABIT*

6 a	Fifi koro Knorr si obe ata mi je ohun ti mo ma nse laironusi.<br>*Adding Knorr Bouillon cubes to my stews is something I do without thinking.*				
	**Mi o fara mo rara**<br>*Disagree Strongly (1)*	**Mi o faramo**<br>*Disagree (2)*	**Mo faramo/Mi o faramo**<br>*Neither agree/Nor disagree (3)*	**Mo Faramo**<br>*Agree (4)*	**Mo Faramo gan**<br>*Agree Strongly (5)*
b	Fifi koro Knorr si obe ata mi je ohun ti mo ma nse lai se pe mo ni lati ranti ki nto se.<br>*Adding Knorr Bouillon cubes to my stews is something I do without having to consciously remember.*				
	**Mi o fara mo rara**<br>*Disagree Strongly (1)*	**Mi o faramo**<br>*Disagree (2)*	**Mo faramo/Mi o faramo**<br>*Neither agree/Nor disagree (3)*	**Mo Faramo**<br>*Agree (4)*	**Mo Faramo gan**<br>*Agree Strongly (5)*
c	Fifi koro Knorr si obe ata mi je ohun ti ma ti bere si ise ki nto rip e mo nse.<br>*Adding Knorr Bouillon cubes to my stews is something I start doing before I realize I'm doing it.*				
	**Mi o fara mo rara**<br>*Disagree Strongly (1)*	**Mi o faramo**<br>*Disagree (2)*	**Mo faramo/Mi o faramo**<br>*Neither agree/Nor disagree (3)*	**Mo Faramo**<br>*Agree (4)*	**Mo Faramo gan**<br>*Agree Strongly (5)*

5_7. *INTENTION*

5_7a	Mo pero lati fi koro Knorr si obe ata mi ni awon osu ti o nbo.<br>*I intend to add Knorr Bouillon cubes to my stews in the next months.*				
	**Ko le sele rara**<br>*Extremely unlikely (1)*	**Ko le sele**<br>*Unlikely (2)*	**Kosiyato**<br>*Neither likely nor unlikely (3)*	**O le sele**<br>*Likely (4)*	**O le sele daada**<br>*Extremely likely (5)*
b	Mo pile lati fi koro Knorr si obe ata ni awon osu to nbo.<br>*I plan to add Knorr Bouillon cubes to my stews in the next months.*				
	**Mi o fara mo rara**<br>*Disagree Strongly (1)*	**Mi o faramo**<br>*Disagree (2)*	**Mo faramo/Mi o faramo**<br>*Neither agree/Nor disagree (3)*	**Mo Faramo**<br>*Agree (4)*	**Mo Faramo gan**<br>*Agree Strongly (5)*
c	Maa fi koro Knorr si obe ata ti ma se ni awon osu ti o n bo.<br>*I will add Knorr Bouillon cubes to the stews I make in the next months.*				
	**Mi o fara mo rara**<br>*Disagree Strongly (1)*	**Mi o faramo**<br>*Disagree (2)*	**Mo faramo/Mi o faramo**<br>*Neither agree/Nor disagree (3)*	**Mo Faramo**<br>*Agree (4)*	**Mo Faramo gan**<br>*Agree Strongly (5)*

GREEN LEAFY VEGETABLES

5_8. *ATTITUDE*

5_8	Fun mi, fifi ewebe (bi soko, tete, ati ugu) sinu obe ata mi ma nje ki obe ata mi.... *For me, adding green leafy vegetables (e.g., ugwu, spinach, amaranth) to my stews, makes my stews …*				
a	**Ko ma wu oju ri rara** *Very unpleasant (1)*	**Ko ma wu oju ri** *Unpleasant (2)*	**Kosi yato** *Not unpleasant, not pleasant (3)*	**Ko wu oju ri** *Pleasant (4)*	**Ko wu oju ri daradara** *Very pleasant (5)*
b	**Ko baje gaan** *Very bad (1)*	**Ko baje** *Bad (2)*	**Ko baje, ko dara** *Not bad, not good (3)*	**Ko dara** *Good (4)*	**Ko dara gan** *Very good (5)*
c	**O fun ni ni ailera gaan** *Very unhealthy (1)*	**O fun ni ni ailera** *Unhealthy (2)*	**Kosiyato** *Not unhealthy, not healthy (3)*	**o fun ni ni ilera** *Healthy (4)*	**O fun ni ni ilera dara dara** *Very healthy (5)*
d	**O ma te rara** *Not at all tasty (1)*	**O te** *Not so tasty (2)*	**Kosiyato** *Neutral/undecided (3)*	**Ko te** *Tasty (4)*	**Ko te gan** *Very tasty (5)*
e	**Ko ma dun rara** *Not at all delicious (1)*	**Ko ma dun** *Not so delicious (2)*	**Kosiyato** *Neutral/undecided (3)*	**Ko dun** *Delicious (4)*	**Ko dun gan** *Very delicious (5)*
f	**Ko ma ri ni lara rara** *Not at all disgusting (1)*	**Ko ma ri ni lara** *Not so disgusting (2)*	**Kosiyato** *Neutral/undecided (3)*	**Ko ri ni lara** *Disgusting (4)*	**Ko ri ni lara gan** *Very disgusting (5)*

5_9. *SOCIAL NORM (INJUNCTIVE/HUSBAND/FATHER)*

[DO NOT ASK 5_9 IF MOTHER IS A WIDOW OR DIVORCED]

5_9a	Oko (baba) mi ro pe o ye ki n fi efo sinu obe ata mi. *My husband/father thinks I should add green leafy vegetables to my stews.*				
	**Mi o fara mo rara** *Disagree Strongly (1)*	**Mi o faramo** *Disagree (2)*	**Mo faramo/Mi o faramo** *Neither agree/Nor disagree (3)*	**Mo faramo** *Agree (4)*	**Mo faramo gan** *Agree Strongly (5)*
b	Oko (baba) mi ro pe o ye ki n fi efo sinu obe mi ti mo ban sebe ata. *My husband/father expects me to add green leafy vegetables when cooking stews.*				
	**Mi o fara mo rara** *Disagree Strongly (1)*	**Mi o faramo** *Disagree (2)*	**Mo faramo/Mi o faramo** *Neither agree/Nor disagree (3)*	**Mo Faramo** *Agree (4)*	**Mo Faramo gan** *Agree Strongly (5)*
c	Ti mo ba fi efo si obe ata mi, oko (baba) mi... *When I add green leafy vegetables to my stew, my husband/father would.*				
	**Ko ni gba rara** *Disapprove strongly (1)*	**Ko ni gba** *Disapprove (2)*	**O le gba/o le ma gba** *Neither approve nor disapprove (3)*	**Yi o gba** *Approve (4)*	**Yi o gba gan** *Approve strongly (5)*

5_10. *Social Norm (Injunctive)*

5_10a	Opolopo awon to se pataki simi ro pe o ye ki nfi efo sobe ata mi.<br>*Most people who are important to me think I should add green leafy vegetables to my stews.*				
	**Mi o fara mo rara**<br>*Disagree Strongly (1)*	**Mi o faramo**<br>*Disagree (2)*	**Mo faramo/Mi o faramo**<br>*Neither agree/Nor disagree (3)*	**Mo Faramo**<br>*Agree (4)*	**Mo Faramo gan**<br>*Agree Strongly (5)*
b	Mo ro pe awon eyan ro pe ki nfi efo si obe ti mo ba nse obe ata.<br>*I think others expect me to add green leafy vegetables when cooking stews.*				
	**Mi o fara mo rara**<br>*Disagree Strongly (1)*	**Mi o faramo**<br>*Disagree (2)*	**Mo faramo/Mi o faramo**<br>*Neither agree/Nor disagree (3)*	**Mo Faramo**<br>*Agree (4)*	**Mo Faramo gan**<br>*Agree Strongly (5)*
c	Ti mo ba fi efo si obe ata mi, awon to se pataki ninu aye mi…<br>*When I add green leafy vegetables to my stew, the people in my life whose opinions I value would…*				
	**Ko ni gba rara**<br>*Disapprove strongly (1)*	**Ko ni gba**<br>*Disapprove (2)*	**Ko ni gba/Gba**<br>*Neither approve nor disapprove (3)*	**Yi o gba**<br>*Approve (4)*	**Yi o gba gan**<br>*Approve strongly (5)*

5_11. *Social Norm (Deductive)*

5_11a	Opolopo awon to se pataki si mi ma n fi efo sobe ata won.<br>*Most people who are important to me add green leafy vegetables to stews.*				
	**Mi o fara mo rara**<br>*Disagree Strongly (1)*	**Mi o faramo**<br>*Disagree (2)*	**Mo faramo/Mi o faramo**<br>*Neither agree/Nor disagree (3)*	**Mo faramo**<br>*Agree (4)*	**Mo faramo gan**<br>*Agree Strongly (5)*
b	Awon eniyan ti imoran won se pataki saye mi, ma n fi efo sobe ata won.<br>*The people in my life whose opinion I value, add green leafy vegetables to their stews.*				
	**Mi o fara mo rara**<br>*Disagree Strongly (1)*	**Mi o faramo**<br>*Disagree (2)*	**Mo faramo/Mi o faramo**<br>*Neither agree/Nor disagree (3)*	**Mo faramo**<br>*Agree (4)*	**Mo Fframo gan**<br>*Agree Strongly (5)*
c	Ninu agbegbe yin, eniyan melo ni e le so pe oun fi efo sobe ata won?<br>*In your community, how many people would you say add green leafy vegetables to their stews?*				
	**Won Kere gan**<br>*Very few 1*	**Won kere**<br>*Quite a few 2*	**Won to ilaji**<br>*About half 3*	**Won po**<br>*A lot 4*	**Yi o fe je gbogbo won**<br>*Almost all 5*

5_12. *PERCEIVED BEHAVIOURAL CONTROL*

5_12a	Fun emi, fifi efo si gbogbo obe ata ti mo ba se…<br>*For me, adding green leafy vegetables to every stews I cook would be…*				
	**Yi o soro gan**<br>*Very difficult (1)*	**Yi o soro**<br>*Difficult (2)*	**Kosoro, kororun**<br>*Not difficult, not easy (3)*	**Yi o rorun**<br>*Easy (4)*	**Yi o rorun pupo**<br>*Very easy (5)*
b	Ti o ba wumi, mo le fi efo si gbogbo obe ata ti mo ba se.<br>*If I wanted to, I could add green leafy vegetables to every stew I cook.*				
	**Mi o fara mo rara**<br>*Disagree Strongly (1)*	**Mi o faramo**<br>*Disagree (2)*	**Mo Faramo/Mi o faramo**<br>*Neither agree/Nor disagree (3)*	**Mo Faramo**<br>*Agree (4)*	**Mo Faramo gan**<br>*Agree Strongly (5)*
c	Bawo ni o se wa lowo yin si lati fi efo si gbogbo obe ata ti e ban se?<br>*To what extent is it up to you to add green leafy vegetables to the stews you cook?*				
	**Gbogbo re wa lowo elomiran**<br>*Completely up to others (1)*	**O wa lowo elomiran lopo igba**<br>*Mostly up to others 2*	**Wa laarin elomiran ati emi**<br>*Both up to others and up to me (3)*	**O wa lowo mi lopo igba**<br>*Mostly up to me (4)*	**O wa lowo emi nikan soso**<br>*Completely up to me (5)*

5_13. *HABIT*

5_13a	Fifi efo sobe ata mi je ohun to ma n sele.<br>*Adding green leafy vegetables to my stews is something I do without thinking.*				
	**Mi o fara mo rara**<br>*Disagree Strongly (1)*	**Mi o faramo**<br>*Disagree (2)*	**Mo faramo/Mi o faramo**<br>*Neither agree/Nor disagree (3)*	**Mo faramo**<br>*Agree (4)*	**Mo faramo gan**<br>*Agree Strongly (5)*
b	Fifi efo sobe ata mi je ohun ti mo ma nse lai gbiyanju lati ranti.<br>*Adding green leafy vegetables to my stews is something I do without having to consciously remember.*				
	**Mi o fara mo rara**<br>*Disagree Strongly (1)*	**Mi o faramo**<br>*Disagree (2)*	**Mo faramo/Mi o faramo**<br>*Neither agree/Nor disagree (3)*	**Mo faramo**<br>*Agree (4)*	**Mo faramo gan**<br>*Agree Strongly (5)*
c	Fifi efo sobe ata mi je ohun ti mo beere ki n to fiyesi pe mo ti n se.<br>*Adding green leafy vegetablesto my stews is something I start doing before I realize I'm doing it.*				
	**Mi o fara mo rara**<br>*Disagree Strongly (1)*	**Mi o faramo**<br>*Disagree (2)*	**Mo faramo/Mi o faramo**<br>*Neither agree/Nor disagree (3)*	**Mo faramo**<br>*Agree (4)*	**Mo faramo gan**<br>*Agree Strongly (5)*

5_14. *INTENTION*

5_14a	Mo pero lati fi efo sobe ata mi ni awon osu to n bo.<br>*I intend to add green leafy vegetables to my stews in the next months.*				
	**Ko le sele rara**<br>*Extremely unlikely (1)*	**Ko le sele**<br>*Unlikely (2)*	**kosiyato**<br>*Neither likely nor unlikely (3)*	**O le sele**<br>*Likely (4)*	**O le sele daada**<br>*Extremely likely (5)*
b	Mo pinu lati fi efo sobe ata mi ni osu to n bo.<br>*I plan to add green leafy vegetables to my stews in the next months.*				
	**Mi o fara mo rara**<br>*Disagree Strongly (1)*	**Mi o faramo**<br>*Disagree (2)*	**Mo faramo/Mi o faramo**<br>*Neither agree/Nor disagree (3)*	**Mo faramo**<br>*Agree (4)*	**Mo faramo gan**<br>*Agree Strongly (5)*
c	Mo ma fi efo sobe ata mi ni awon osu to n bo.<br>*I will add green leafy vegetables to the stews I make in the next months.*				
	**Mi o fara mo rara**<br>*Disagree Strongly (1)*	**Mi o faramo**<br>*Disagree (2)*	**Mo faramo/Mi o faramo**<br>*Neither agree/Nor disagree (3)*	**Mo faramo**<br>*Agree (4)*	**Mo faramo gan**<br>*Agree Strongly (5)*

SECTION 6: MOTHER – DAUGHTER INTERACTION

INTERVIEWER, PLEASE READ OUT:

"E yi ni abala to pari ibeere naa."

["THIS IS THE LAST SET OF QUESTIONS"]

[INTERVIEWER, PLEASE READ OUT EACH OF THE "ISSUES DISCUSSED" AND THEN READ OUT THE OPTIONS FROM "HARDLY EVER OR NEVER" TO "MORE THAN ONCE A DAY". NOTE THE RESPONSE OF THE RESPONDENT.]

6. Nje e le so fun wa bii igba melo ni e ma n ba omobinrin (iya) yin se ajoro lori awon oro ti a ko sisale wonyi?

(Please can you indicate how often you discuss the following subjects with your daughter/mother?)

	Ko to eekan losu tabi rara<br>*Less than once a month or never (1)*	Eekan si emeta larin osu<br>*1 -3 times a month (2)*	Eekan lose<br>*Once a week (3)*	Eemeta si eemarun lose<br>*3-5 times a week (4)*	Eekan lojumo<br>*Once a day (5)*	Ju eekan lo lojumo<br>*More than once a day (6)*
Oye lori ina dida<br>*(Cooking skills)*						
Didana ti o n fun ni ni ilera<br>*(How to cook healthily)*						
Ooyi kiko ni, rire ara<br>*(Feeling dizzy, tired)*						
Bi omobinrin yin (eyin) ti n se ni ile iwe<br>*(How your daughter (you) is doing at school)*						
Ohun ti o ma n ba awon ore re jiroro le<br>*(What she discusses with her friends)*						
Bi omobinrin yin (eyin) se le fi okan ba nkan lo si<br>*(How well your daughter (you) can concentrate)*						
Iye agbara ti e ro pe e ni<br>*(How much energy you feel you have)*						

TIME: ENDED: ____/____/ (HH/MM)

E SEUN FUN IDAHUN ATI ASIKO YIN

THANK YOU FOR YOUR ANSWERS AND THE TIME SPENT WITH ME
