# Supplementary material for: The effect of the “Follow in my Green Food Steps” programme on cooking behaviours for improved iron intake: a quasi-experimental randomized community study
Source: Int J Behav Nutr Phys Act. 2018 Aug 16;15:79. doi: 10.1186/s12966-018-0710-4 (PMC6097342; doi:10.1186/s12966-018-0710-4)
Supplement: Supplementary file 4 — Post-Intervention Questionnaire. (PDF 1558 kb) [file 12966_2018_710_MOESM4_ESM.pdf]

TO BE FILLED OUT BY PRINCIPAL INVESTIGATORS ONLY:

UNIQUE ID:

UNIVERSITY OF IBADAN

POST-INTERVENTION QUESTIONNAIRE

FOLLOW IN MY FOOD STEPS BEHAVIOUR CHANGE IMPACT STUDY

YORUBA VERSION

INTERVIEWER: PLEASE NOTE THE PARTICIPANT NUMBER ON THE ADDRESS SHEET UNDER "INTERVIEW NUMBER". PLEASE TAKE CARE TO ALSO NOTE THE INTERVIEWER NUMBER WITH WHICH IT IS PAIRED.

Reference Number: SPB01_2015

INTERVIEW NR____________

1. Initials of Interviewer:______________ 2. Date (DD/MM/YY): ____/____/2016

3. State:_________________________ 4. LGA: ___________________ PAIRED WITH INT NR: _____

5. Name of Community_______________ 6. Time: Started: ____/____/ (hh/mm)

7. Mother's Baseline Interviewer NR:_____________________

[INTERVIEWER, PLEASE NOTE: YOU DO NOT HAVE TO READ OUT THE FULL CONSENT FORM AGAIN]
INTERVIEWER, PLEASE READ OUT:

E se fun gbigba lati kopa ninu abala keji ise iwadi yi. ki a to bere iforowanilenuwo, mo nife lati so pe kikopa je lati inu wa ni. a nife lati mo ihuwasi ati awon ero yin, nipa bayi ema se alae dahun awon ibeere yi lododo. Gbogbo awon idahun ti e ba fun wa ni yio wa ni bonkele ti a ko si ni le topa re si odo yin. E ni anfani lati dekun kikopa nigbaki ti kosi ni si alebu kankan fun yin.

[THANK YOU FOR AGREEING TO TAKE PART IN THIS SECOND PART OF THE RESEARCH. BEFORE WE START WITH THE INTERVIEW, I WOULD LIKE TO MENTION AGAIN THAT YOUR PARTICIPATION IS COMPLETELY VOLUNTARY. WE ARE INTERESTED IN YOUR PERSONAL BEHAVIOURS AND OPINIONS, SO PLEASE DON'T HESITATE TO GIVE HONEST ANSWERS. ALL ANSWERS YOU PROVIDE WILL BE KEPT CONFIDENTIAL AND CANNOT BE TRACED BACK TO YOU PERSONALLY. YOU ARE FREE TO STOP PARTICIPATING AT ANY TIME AND THIS WILL NOT AFFECT YOU IN ANY WAY].

TO BE FILLED OUT BY THE INTERVIEWER. DO NOT READ THIS OUT.

I, ________________[name of interviewer] declare that before starting the interview I have made clear that
- the respondent's participation is completely voluntary
- all answers provided will be kept confidential and cannot be traced back to the participant
- the respondent is free to stop participating at any time and this will not affect the respondent in any way

Signature of interviewer: ____ Date: ______________________

DEMOGRAPHICS (FOR ROUTING)

**Sp_1.** Kini ipo igbeyawo yin?<br>*(What is your marital status?)*		
1	Abileko/ ajumo gbe po pelu enikeji mi *(Married / living together with partner)*	
2	Opo *(Widowed)*	
3	Ati pinya *(Divorced)*	
4	Ipo miran, jowo so *(Other, please specify):* ___________________________________	

**Sp_2a.** Ile iwe wo ni omo yin obinrin nlo (omo obinrin yin ti o nkopa ninu ise iwadi yi)?<br>*(What school does your daughter attend (your daughter involved in this study)?)*		
1		

**Sp_2b.** Kilaasi wo ni omo yin obinrin wa (omo obinrin yin ti o nkopa ninu ise iwadi yi)?<br>*(In which class is your daughter (your daughter involved in this study)?)*		
1		

SECTION 7: FOOD INTAKE QUESTIONNAIRE

[INTERVIEWER, PLEASE READ OUT:]

"Ninu awon ibeere ti o kan, a fe lati mo si nipa bawo ni e se se obe ata si larin ose meji seyin, awon eroja ti e saba ma nlo lati se won ounje wonyi ati odiwon awon eroja ti e lo".

("IN THE NEXT QUESTIONS, WE WANT TO KNOW MORE ABOUT HOW OFTEN YOU HAVE COOKED STEWS AND SOUPS DURING THE PAST TWO WEEKS, WHICH INGREDIENTS YOU USUALLY USE TO MAKE THESE DISHES AND HOW MUCH OF THESE INGREDIENTS YOU USE").

A. OBE ATA (STEWS)

7_A1. Laarin ose meji to koja, bii emelo ni e ti se ninu awon obe wonyi?
(During the past two weeks, how often have you cooked any of the following stews?)

	**Obe ata eleran malu** *(Beef stew)*	**Obe ata eleja** *(Fish stew)*	**Obe ata ogufe** *(Goat stew)*	**Obe ata didin** *(Fried stew)*	**Obe ata adie/tolotolo** *(Chicken/Turkey)*	**Obe ata miran** *(Other)*
INDICATE NUMBER OF TIMES DURING PAST 2 WEEKS						

7_A2A. OBE ATA ELERAN MALU (BEEF STEW)					
**7_A2A.** Ti e ba n se koko obe ata eran malu<br>- Ewo ninu awon eroja ti a ka sile yi ni e fi kun?<br>- Bawo ni eroja ti e fi kun ti po to?			*When cooking a pot of beef stew*<br>- *Which of the following ingredients did you add?*<br>- *How much of each ingredient did you add?*		
**ORUKO EROJA (NAME OF INGREDIENT)**		**MAAKI INU APOTI YI TI O BA FI SI (TICK BOX IF ADDED)**	**SO IYE OSUWON TI O FI SI NU KOKO OBE (INDICATE NUMBER OF UNITS PER POT)**	**OSUWON (UNIT) CIRCLE OR UNDERLINE APPROPRIATE ANSWER**	
Eran malu	*(Beef)*			Naira	
Alubosa	*(Onions)*			(Kekere/Odinwon/ Nla)	*(Small/medium/large)*
Tatase	*(Red bell pepper)*			(Kobiowu/lanbebe kekere/lanbebe nla)	*(Selling bowl large/ medium/small)*
Ata rodo	*(Spicy red pepper)*			(Kobiowu/lanbebe kekere/lanbebe nla)	*(Selling bowl large/ medium/small)*
Tomati	*(Tomatoes)*			(Kobiowu/lanbebe kekere/lanbebe nla)	*(Selling bowl large/ medium/small)*
Isebe Knorr onikoro	*(Knorr cubes)*			Iye koro	*(Cubes)*
Isebe onikoro miran	*(Other Cubes)*			Iye koro	*(Cubes)*
Ewebe (ugu, soko, tete, ewe ege, ewe koko)	*(Green leafy vegetables (ugu, spinach, amaranth leaves, cocoyam leaves)*			Iye idi	*(Bunches)*
Omiran: …………………..	*(Other:……………….)*				
Omiran: …………………..	*(Other: …………….)*				

7_A2B. OBE ATA EL EJA (FISH STEW)					
**7_A2B.** Ti e ba n se koko obe ata el eja<br>- Ewo ninu awon eroja ti a ka sile yi ni e fi kun?<br>- Bawo ni eroja ti e fi kun ti po to?			*When cooking a pot of fish stew*<br>- *Which of the following ingredients did you add?*<br>- *How much of each ingredient did you add?*		
ORUKO EROJA (NAME OF INGREDIENT)		MAAKI INU APOTI YI TI O BA FI SI (TICK BOX IF ADDED)	SO IYE OSUWON TI O FI SI NU KOKO OBE (INDICATE NUMBER OF UNITS PER POT)	OSUWON (UNIT) CIRCLE OR UNDERLINE APPROPRIATE ANSWER	
Eja	*(Fish)*			Naira	
Alubosa	*(Onions)*			(Kekere/Odinwon/ Nla)	*(Small/medium/large)*
Tatase	*(Red bell pepper)*			(Kobiowu/lanbebe kekere/lanbebe nla)	*(Selling bowl large/ medium/small)*
Ata rodo	*(Spicy red pepper)*			(Kobiowu/lanbebe kekere/lanbebe nla)	*(Selling bowl large/ medium/small)*
Tomati	*(Tomatoes)*			(Kobiowu/lanbebe kekere/lanbebe nla)	*(Selling bowl large/ medium/small)*
Isebe Knorr onikoro	*(Knorr cubes)*			Iye koro	*(Cubes)*
Isebe onikoro miran	*(Other Cubes)*			Iye koro	*(Cubes)*
Ewebe (ugu, soko, tete, ewe ege, ewe koko)	*(Green leafy vegetables ugu, spinach, amaranth leaves)*			Iye idi	*(Bunches)*
Omiran: …………………..	*(Other:……………….)*				
Omiran: …………………..	*(Other: ……………..)*				

7_A2C. OBE ATA OGUFE (GOAT STEW)					
**7_A2C**. Ti e ba n se koko obe ata ogufe<br>- Ewo ninu awon eroja ti a ka sile yi ni e fi kun?<br>- Bawo ni eroja ti e fi kun ti po to?			*When cooking a pot of goat stew*<br>- *Which of the following ingredients did you add?*<br>- *How much of each ingredient did you add?*		
ORUKO EROJA (NAME OF INGREDIENT)		MAAKI INU APOTI YI TI O BA FI SI (TICK BOX IF ADDED)	SO IYE OSUWON TI O FI SI NU KOKO OBE (INDICATE NUMBER OF UNITS PER POT)	OSUWON (UNIT) CIRCLE OR UNDERLINE APPROPRIATE ANSWER	
Eran ogufe	*(Goat Meat)*			Naira	
Alubosa	*(Onions)*			(Kekere/Odinwon/ Nla)	*(Small/medium/large)*
Tatase	*(Red bell pepper)*			(Kobiowu/lanbebe kekere/lanbebe nla)	*(Selling bowl large/ medium/small)*
Ata rodo	*(Spicy red pepper)*			(Kobiowu/lanbebe kekere/lanbebe nla)	*(Selling bowl large/ medium/small)*
Tomati	*(Tomatoes)*			(Kobiowu/lanbebe kekere/lanbebe nla)	*(Selling bowl large/ medium/small)*
Isebe Knorr onikoro	*(Knorr cubes)*			Iye koro	*(Cubes)*
Isebe onikoro miran	*(Other Cubes)*			Iye koro	*(Cubes)*
Ewebe (ugu, soko, tete, ewe ege, ewe koko)	*(Green leafy vegetables ugu, spinach, amaranth leave),*			Iye idi	*(Bunches)*
Omiran: …………………..	*(Other:……………….)*				
Omiran: …………………..	*(Other: ……………..)*				

7_A2D. OBE ATA DINDIN (FRIED STEW)					
**7_A2D.** Ti e ba n se koko obe ata dindin<br>- Ewo ninu awon eroja ti a ka sile yi ni e fi kun?<br>- Bawo ni eroja ti e fi kun ti po to?			*When cooking a pot of fried stew*<br>- *Which of the following ingredients did you add?*<br>- *How much of each ingredient did you add?*		
ORUKO EROJA (NAME OF INGREDIENT)		MAAKI INU APOTI YI TI O BA FI SI (TICK BOX IF ADDED)	SO IYE OSUWON TI O FI SI NU KOKO OBE (INDICATE NUMBER OF UNITS PER POT)	OSUWON (UNIT) CIRCLE OR UNDERLINE APPROPRIATE ANSWER	
Alubosa	*(Onions)*			(Kekere/Odinwon/ Nla)	*(Small/medium/large)*
Tatase	*(Red bell pepper)*			(Kobiowu/lanbebe kekere/lanbebe nla)	*(Selling bowl large/ medium/small)*
Ata rodo	*(Spicy red pepper)*			(Kobiowu/lanbebe kekere/lanbebe nla)	*(Selling bowl large/ medium/small)*
Tomati	*(Tomatoes)*			(Kobiowu/lanbebe kekere/lanbebe nla)	*(Selling bowl large/ medium/small)*
Isebe Knorr onikoro	*(Knorr cubes)*			Iye koro	*(Cubes)*
Isebe onikoro miran	*(Other Cubes)*			Iye koro	*(Cubes)*
Ewebe (ugu, soko, tete, ewe ege, ewe koko)	*(Green leafy vegetables, ugu, spinach, amaranth leaves)*			Iye idi	*(Bunches)*
Omiran: …………………..	*(Other:……………….)*				
Omiran: …………………..	*(Other: ……………...)*				

7_A2E. OBE ATA ADIE/TOLOTOLO (CHICKEN/TURKEY)					
**7_A2E.** Ti e ba n se koko obe ata adie/tolotolo<br>- Ewo ninu awon eroja ti a ka sile yi ni e fi kun?<br>- Bawo ni eroja ti e fi kun ti po to?			*When cooking a pot of chicken/turkey stew*<br>- *Which of the following ingredients did you add?*<br>- *How much of each ingredient did you add?*		
ORUKO EROJA (NAME OF INGREDIENT)		MAAKI INU APOTI YI TI O BA FI SI (TICK BOX IF ADDED)	SO IYE OSUWON TI O FI SI NU KOKO OBE (INDICATE NUMBER OF UNITS PER POT)	OSUWON (UNIT) CIRCLE OR UNDERLINE APPROPRIATE ANSWER	
Eran adiye	*(Chicken Meat)*			Naira	
Eran tolotolo	*(Turkey Meat)*			Naira	
Alubosa	*(Onions)*			(Kekere/Odinwon/ Nla)	*(Small/medium/large)*
Tatase	*(Red bell pepper)*			(Kobiowu/lanbebe kekere/lanbebe nla)	*(Selling bowl large/ medium/small)*
Ata rodo	*(Spicy red pepper)*			(Kobiowu/lanbebe kekere/lanbebe nla)	*(Selling bowl large/ medium/small)*
Tomati	*(Tomatoes)*			(Kobiowu/lanbebe kekere/lanbebe nla)	*(Selling bowl large/ medium/small)*
Isebe Knorr onikoro	*(Knorr cubes)*			Iye koro	*(Cubes)*
Isebe onikoro miran	*(Other Cubes)*			Iye koro	*(Cubes)*
Ewebe (ugu, soko, tete, ewe ege, ewe koko)	*(Green leafy vegetables, ugu, spinach, amaranth leaves),*			Iye idi	*(Bunches)*
Omiran: …………………..	*(Other:……………….)*				
Omiran: …………………..	*(Other: ……………...)*				

7_A2F. OBE ATA MIRAN: ______________________________ (OTHER STEW______________________)					
**7_A2F.** Ti e ba n se koko obe ata<br>- Ewo ninu awon eroja ti a ka sile yi ni e fi kun?<br>- Bawo ni eroja ti e fi kun ti po to?			*When cooking a pot of stew*<br>- *Which of the following ingredients did you add?*<br>- *How much of each ingredient did you add?*		
**ORUKO EROJA (NAME OF INGREDIENT)**		**MAAKI SI INU APOTI YI TI O BA FI SI (TICK BOX IF ADDED)**	**SO IYE OSUWON TI O FI SI NU KOKO OBE (INDICATE NUMBER OF UNITS PER POT)**	**OSUWON (UNIT) CIRCLE OR UNDERLINE APPROPRIATE ANSWER**	
Eran	*(Meat)*			Naira	
Alubosa	*(Onions)*			(Kekere/Odinwon/ Nla)	*(Small/medium/large)*
Tatase	*(Red bell pepper)*			(Kobiowu/lanbebe kekere/lanbebe nla)	*(Selling bowl large/ medium/small)*
Ata rodo	*(Spicy red pepper)*			(Kobiowu/lanbebe kekere/lanbebe nla)	*(Selling bowl large/ medium/small)*
Tomati	*(Tomatoes)*			(Kobiowu/lanbebe kekere/lanbebe nla)	*(Selling bowl large/ medium/small)*
Isebe Knorr onikoro	*(Knorr cubes)*			Iye koro	*(Cubes)*
Isebe onikoro miran	*(Other Cubes)*			Iye koro	*(Cubes)*
Ewebe (ugu, soko, tete, ewe ege, ewe koko)	*(Green leafy vegetables, ugu, spinach, amaranth leaves)*			Iye idi	*(Bunches)*
Omiran:	*(Other:……………….)*				
Omiran:	*(Other: ………………)*				

B. OBE (Soups)

**7_B1.** Laarin ose meji ti o koja, bii emelo ni e se awon obe ti o wa nisale yi?<br>*(During the past two weeks, how often have you cooked the following soups?)*					
	**Egusi**<br>*(Egusi soup)*	**Efo riro**<br>*(Spinach/Amaranth soup)*	**Ila**<br>*(Okra soup)*	**Ewedu**<br>*(Ewedu soup)*	**Gbegiri**<br>*(Bean soup)*
Indicate number of times during past 2 weeks					

7_B2A. Egusi					
**7_B2a.** Ti e ba n se obe egusi<br>- Ewo ninu awon eroja ti a ka sile yi ni e fi kun?<br>- Bawo ni eroja ti e fi kun tip o to?			*When cooking egusi soup*<br>- *Which of the following ingredients did you add?*<br>- *How much of each ingredient did you add?*		
**Oruko eroja** (Name of ingredient)		Maaki inu apoti yi ti o ba fi si (Tick box if added)	So iye osuwon ti o fi si nu koko obe (Indicate Number of units per pot)	Osuwon (Unit) CIRCLE OR UNDERLINE APPROPRIATE ANSWER	
Eran	*(Meat)*			Naira	
Alubosa	*(Onions)*			(Kekere/Odinwon/ Nla)	*(Small/medium/large)*
Tatase	*(Red bell pepper)*			(Kobiowu/lanbebe kekere/lanbebe nla)	*(Selling bowl large/ medium/small)*
Tomati	*(Tomatoes)*			(Kobiowu/lanbebe kekere/lanbebe nla)	*(Selling bowl large/ medium/small)*
Egusi	*(melon seeds)*			(Agolo miliki)	*(Milk tin)*
Isebe Knorr onikoro	*(Knorr cubes)*			(Iye koro)	*(Cubes)*
Isebe Maggi onikoro	*(Other Cubes)*			(Iye koro)	*(Cubes)*
Ewebe (ugu, soko, tete, ewe ege, ewe koko)	*(Green leafy vegetables, ugu, spinach, amaranth leaves, cassava leaves, cocoyam leaves)*			(Iye idi)	*(Bunches)*
Omiran: …………………..	*(Other:……………….)*				
Omiran: …………………..	*(Other: ……………..)*				

7_B2B. EFO RIRO (SPINACH/AMARANTH)					
**7_B2B.** Ti e ba n se efo riro<br>- Ewo ninu awon eroja ti a ka sile yi ni e fi kun?<br>- Bawo ni eroja ti e fi kun tip o to?				*When cooking spinach/amaranth soup*<br>- *Which of the following ingredients did you add?*<br>- *How much of each ingredient did you add?*	
**ORUKO EROJA (NAME OF INGREDIENT)**		**MAAKI INU APOTI YI TI O BA FI SI (TICK BOX IF ADDED)**	**SO EYE OSUWON TI O FI SI NU KOKO OBE (INDICATE NUMBER OF UNITS PER POT)**	**OSUWON (UNIT) CIRCLE OR UNDERLINE APPROPRIATE ANSWER**	
Eran	*(Meat)*			Naira	
Alubosa	*(Onions)*			(Kekere/Odinwon/ Nla)	*(Small/medium/large)*
Tatase	*(Red bell pepper)*			(Kobiowu/lanbebe kekere/lanbebe nla)	*(Selling bowl large/ medium/small)*
Tomati	*(Tomatoes)*			(Kobiowu/lanbebe kekere/lanbebe nla)	*(Selling bowl large/ medium/small)*
Isebe Knorr onikoro	*(Knorr cubes)*			(Iye koro)	*(Cubes)*
Isebe Maggi onikoro	*(Other Cubes)*			(Iye koro)	*(Cubes)*
Ewebe (ugu, soko, tete, ewe ege, ewe koko)	*(Green leafy vegetables, ugu, spinach, amaranth leaves, cassava leaves, cocoyam leaves)*			(Iye idi)	*(Bunches)*
Omiran: …………………..	*(Other:……………….)*				
Omiran: …………………..	*(Other: ……………..)*				

7_B2C. OBE ILA (OKRA SOUP)					
**7_B2C.** Ti e ba n se obe ila<br>- Ewo ninu awon eroja ti a ka sile yi ni e fi kun?<br>- Bawo ni eroja ti e fi kun tip o to?				*When cooking okra soup*<br>- *Which of the following ingredients did you add?*<br>- *How much of each ingredient did you add?*	
**ORUKO EROJA (NAME OF INGREDIENT)**		**MAAKI INU APOTI YI TI O BA FI SI (TICK BOX IF ADDED)**	**SO EYE OSUWON TI O FI SI NU KOKO OBE (INDICATE NUMBER OF UNITS PER POT)**	**OSUWON (UNIT) CIRCLE OR UNDERLINE APPROPRIATE ANSWER**	
Eran	*(Meat)*			Naira	
Alubosa	*(Onions)*			(Kekere/Odinwon/ Nla)	*(Small/medium/large)*
Tatase	*(Red bell pepper)*			(Kobiowu/lanbebe kekere/lanbebe nla)	*(Selling bowl large/ medium/small)*
Tomati	*(Tomatoes)*			(Kobiowu/lanbebe kekere/lanbebe nla)	*(Selling bowl large/ medium/small)*
Egusi	*(melon seeds)*			(Agolo miliki)	*(Milk tin)*
Ila	*(Okra)*			(Lanbebe kekere/lanbebe nla)	*(Selling bowl medium/small)*
Isebe Knorr onikoro	*(Knorr cubes)*			(Iye koro)	*(Cubes)*
Isebe Maggi onikoro	*(Other Cubes)*			(Iye koro)	*(Cubes)*
Ewebe (ugu, soko, tete, ewe ege, ewe koko)	*(Green leafy vegetables, ugu, spinach, amaranth leaves, cassava leaves, cocoyam leaves)*			(Iye idi)	*(Bunches)*
Omiran: …………………..	*(Other:……………….)*				
Omiran: …………………..	*(Other: ……………..)*				

7_B2D. EWEDU					
**7_B2D.** Ti e ba n se obe ewedu<br>- Ewo ninu awon eroja ti a ka sile yi ni e fi kun?<br>- Bawo ni eroja ti e fi kun tip o to?			*When cooking ewedu soup*<br>- *Which of the following ingredients did you add?*<br>- *How much of each ingredient did you add?*		
**ORUKO EROJA (NAME OF INGREDIENT)**		**MAAKI INU APOTI YI TI O BA FI SI (TICK BOX IF ADDED)**	**SO EYE OSUWON TI O FI SI NU KOKO OBE (INDICATE NUMBER OF UNITS PER POT)**	**OSUWON (UNIT) CIRCLE OR UNDERLINE APPROPRIATE ANSWER**	
Eran	*(Meat)*			Naira	
Alubosa	*(Onions)*			(Kekere/Odinwon/ Nla)	*(Small/medium/large)*
Tatase	*(Red bell pepper)*			(Kobiowu/lanbebe kekere/lanbebe nla)	*(Selling bowl large/ medium/small)*
Tomati	*(Tomatoes)*			(Kobiowu/lanbebe kekere/lanbebe nla)	*(Selling bowl large/ medium/small)*
Egusi	*(melon seeds)*			(Agolo miliki)	*(Milk tin)*
Isebe Knorr onikoro	*(Knorr cubes)*			(Iye koro)	*(Cubes)*
Isebe Maggi onikoro	*(Other Cubes)*			(Iye koro)	*(Cubes)*
Ewebe (ugu, soko, tete, ewe ege, ewe koko)	*(Green leafy vegetables, ugu, spinach, amaranth leaves, cassava leaves, cocoyam leaves)*			(Iye idi)	*(Bunches)*
Omiran:	*(Other:……………….)*				
Omiran:	*(Other: ……………..)*				

7_B2E. GBEGIRI (BEAN SOUP)					
**7_B2E.** Ti e ba n se obe gbegiri<br>- Ewo ninu awon eroja ti a ka sile yi ni e fi kun?<br>- Bawo ni eroja ti e fi kun tip o to?			*When cooking bean soup*<br>- *Which of the following ingredients did you add?*<br>- *How much of each ingredient did you add?*		
**ORUKO EROJA (NAME OF INGREDIENT)**		**MAAKI INU APOTI YI TI O BA FI SI (TICK BOX IF ADDED)**	**SO EYE OSUWON TI O FI SI NU KOKO OBE (INDICATE NUMBER OF UNITS PER POT)**	**OSUWON (UNIT) CIRCLE OR UNDERLINE APPROPRIATE ANSWER**	
Eran	*(Meat)*			Naira	
Alubosa	*(Onions)*			(Kekere/Odinwon/ Nla)	*(Small/medium/large)*
Tatase	*(Red bell pepper)*			(Kobiowu/lanbebe kekere/lanbebe nla)	*(Selling bowl large/ medium/small)*
Tomati	*(Tomatoes)*			(Kobiowu/lanbebe kekere/lanbebe nla)	*(Selling bowl large/ medium/small)*
Ewa	*(beans)*			(Agolo miliki)	*(Milk tin)*
Isebe Knorr onikoro	*(Knorr cubes)*			(Iye koro)	*(Cubes)*
Isebe Maggi onikoro	*(Other Cubes)*			(Iye koro)	*(Cubes)*
Ewebe (ugu, soko, tete, ewe ege, ewe koko)	*(Green leafy vegetables, ugu, spinach, amaranth leaves, cassava leaves, cocoyam leaves)*			(Iye idi)	*(Bunches)*
Omiran:	*(Other:……………….)*				
Omiran:	*(Other: ……………..)*				

**7_C.** Bawo le se se irufe awon ounje yii si ninu ose meji to koja?<br>*(How often have you prepared any of the following dishes during the past two weeks?)*									
	Iresi fufun sise *(Boiled rice)*	Iresi eleroja ata *(Jollof rice)*	Abo efo ni egbe kan *(side dish of green leafy vegetables)*	Iresi dindin *(fried rice)*	Ewa sisi *(Cooked Beans)*	Omiran ________ *(Other)*	Omiran ________ *(Other)*	Omiran ________ *(Other)*	Omiran ________ *(Other)*
INDICATE NUMBER OF TIMES DURING PAST 2 WEEKS									

SECTION 8: KNOWLEDGE/AWARENESS QUESTIONS ON IRON DEFICIENCY

[INTERVIEWER, PLEASE READ OUT:]

"Ninu awon ibeere ti o kan, a nife lati ni oye ati owon ohun ti e mo nipa aito eroja iron (aito eje), awon ami ati ohun ti o le ti eyin re yo. Ma fe fi idi re mule wipe eleyi kii se idanwo, mo nifesi idahun yin ni otito".

("IN THE NEXT FEW QUESTIONS, WE WOULD LIKE TO UNDERSTAND HOW MUCH YOU KNOW ABOUT IRON DEFICIENCY (ANAEMIA), ITS SYMPTOMS AND ITS CONSEQUENCES. I WOULD LIKE TO EMPHASISE THAT THIS IS NOT A TEST, I AM INTERESTED IN YOUR HONEST ANSWER.")

8_K1. Bawo ni e se ma n ri awon apere yi si?
(How often do you experience the following symptoms?)

	O din ni ekan losu tabi rara *(Less than once a month or never)*	Ekan si emeta losu *(1 – 3 times a month)*	Ekan lose *(Once a week)*	Emeta si emarun lose *(3-5 times a week)*	Ekan lojumo *(Once a day)*	Ju ekan lo lojumo *(More than once a day)*
Aile fi okan si ibi nkan daadaa *(Poor concentration)*						
Rire ni bi o ti le je pe a sun daa daa *(Tiredness, even though you have had enough sleep)*						
Maa kanra tabi binu lai si ohun kan ti o gbodi *(Increased irritability, when nothing has really gone wrong)*						
Oyi kikoni *(Dizziness)*						
Sise funfun awo ara *(Paler complexion)*						

8_K2. Nje e ti gbo nipa aisan kan ti a n pe ni "aisan aito eje lara"?
*(Have you ever heard of a condition called "**anaemia**"/"iron deficiency"? (note: translated as "insufficient blood")*

1	Beeni *(Yes)*		CONTINUE WITH 8_K3
2	Beeko *(No)*		CONTINUE WITH 8_K5

[READ THE NEXT QUESTION AND READ OUT THE OPTIONS. **TICK IF RESPONSE IS "YES". DO NOT TICK IF RESPONDENT INDICATES "NO" OR "DON'T KNOW". MULTIPLE CODING POSSIBLE.]**

8_K3. Ewo ninu awon ipo yi ni o le je ki eniyan ni aito eje?

(According to you, which of these conditions/situations might increase the risk of anaemia?)

1	Idile ti o ni itan aito eje	*(Family history of anaemia)*	
2	Kikere ju bi o se ye lo	*(Being underweight)*	
3	Titobi ju bi o se ye lo	*(Being overweight/obese)*	
4	Ikun ti o ba tobi	*(Having a large waist/ a tummy)*	
5	Ounje ti o ni ora pupo	*(High fat diet)*	
6	Idaamu/ aifokanbale/inira	*(Stress/ tension)*	
7	Jije suga ati ohun ti o dun ju	*(Excessive intake of sweets/ sugar)*	
8	Ai maa se ere idaraya	*(Lack of physical activity)*	
9	Ai ma je ewebe ti a sese ja bi ugu, efo, soko, tete, ewe ege, ewe koko to	*(Low intake of green leafy vegetables such as ugu, spinach, cassava leaves, cocoyam leaves)*	
10	Ai ma je ewebe alata (tomati, ata rodo,) to	*(Low intake of red/orange vegetables (e.g., red pepper, tomato)*	
11	Ai ma je ewebe onikoro bii ewa elewe to	*(Low intake of green vegetables such as green beans, okra)*	
12	Ai maa je ewebe alawo aluko, fufun (bii irugbin igba, ege) to	*(Low intake of purple, white vegetables (e.g., garden egg, cassava)*	
13	Ai maa je eso to	*(Low intake of fruits)*	
14	Ai maa je eran pupa (maalu, ewure) to	*(Low intake of red meat (beef, goat)*	
15	Ai maa je eja to	*(Low intake of fish)*	
16	Ai maa je eyin to	*(Low intake of eggs)*	
17	Ai maa je ewa to	*(Low intake of beans)*	
18	Ai maa sun to	*(Insufficient sleep)*	
19	Siga fifa	*(Smoking)*	
20	Liloyun	*(Being pregnant)*	
21	Mi ma se nkan osu	*(Having your period)*	
23	Ailera ara	*(Being sick)*	
24	Nini aran	*(Having worms)*	
25	Awon ohun miran (e so) ___________________________________	*(Others (Specify)* ______________________________	

[READ THE QUESTION AND READ OUT THE OPTIONS. TICK RESPONSES PROVIDED BY RESPONDENT. **MULTIPLE CODING POSSIBLE]**

**8_K4.** Ewo ninu awon wonyi ni ona abayo ti o muna doko fun aito eje lara?<br>*(Which of these are effective solutions for "anaemia" or "iron deficiency"?)*					UNAIDED
			**Beeni**<br>*Yes (1)*	**Beeko**<br>*No (2)*	**Mi o mo**<br>*(Don't know)*
1	Ki a ma je efo gege bi ara ounje wa	*(Eating vegetables as part of a meal)*			
2	Ki a ma gun, lo, ati mu omi ti a ri lara ewe ugu	*(Crushing, grinding and drinking the juice from pumpkin leaves)*			
3	Mimu egbogi ibile orisirisi ti a sepo	*(Drinking herbal mixes)*			
4	Jije ogede ti ko pon	*(Eating unripe plantains)*			
5	Jije edo	*(Consuming liver)*			
6	Lilo "ogun eje olomi" tabi "ogun eje onikoro'	*(Consuming 'blood tonic' or 'blood tablets)*			
7	Ki a ma je ewebe (bi ugu, efo, tete, soko) ti a sese ja	*(Consuming green leafy vegetables, such as ugu, spinach, amaranth leaves)*			
8	Ki a ma je ewebe pupa bi ata rodo, tatase, timati elegede	*(Consuming red/orange vegetables such as red pepper, tomato, pumpkins)*			
9	Jije eso	*(Consuming fruits)*			
10	Jije eran ju ti ateyin wa lo (bii, maalu, ewure)	*(Consuming more meats (e.g., beef, goat)*			
11	Jije eyin ju ti ateyin wa lo	*(Consuming more eggs)*			
12	Jije eja ju ti ateyin wa lo	*(Consuming more fish)*			
13	Jije ewa ju ti ateyin wa lo	*(Consuming more beans)*			
14	Ki a ma sun ju ti tele lo	*(Getting more sleep)*			
15	Ki a ma se ere idaraya ju ti tele lo	*(Being more physically active)*			
16	Awon ohun miran (e so) ______________________	*(Others (Specify)* ______________________			

[READ THE STATEMENTS AND READ OUT THE ANSWER OPTIONS. TICK RESPONSES PROVIDED BY RESPONDENT. IF THE RESPONDENT REALLY DOES NOT KNOW, TICK "DON'T KNOW", BUT DO NOT PROMPT]

8_K5. E jowo e mu eyi ti o ba so bi e se faramo tabi lodi si awon oro ti a ko si isale wonyi.
(Please indicate for the following statements how much you agree or disagree with the following statements.)

		**Mi o fara mo rara**<br>*Disagree Strongly (1)*	**Mi o faramo die**<br>*Disagree Slightly (2)*	**Mo faramo/Mi o faramo**<br>*Neither agree/Nor disagree (3)*	**Mo fara mo die**<br>*Agree Slightly (4)*	**Mo faramo gan**<br>*Agree Strongly (5)*	**Mi o mo**<br>*I don't know (9)*
1	Eroja iron dara fun ilera<br>*(Iron is good for the health)*						
2	Jije ounje ti o ni eronja eje ninu nse iranlowo lati pese eje<br>*(Eating foods containing iron helps build blood*						
3	Ounje ti o ni eroja eje ninu maa nran awon soja ara lowo<br>*(Foods containing iron strengthens the immune system)*						
4	Eroja eje iron ko ni iwulo kankan fun ilera ara<br>*(Iron has no healthy benefit for the body)*						
5	A ko le je eroja ti o nfun ni ni eje ni apoju<br>*(You cannot consume too much iron)*						
6	Eroja iron nran eje lowo lati gbe ategun kiri ago ara wa<br>*(Iron helps your blood carry oxygen through the body)*						
7	Jije ounje ti o ni eroja eje ma nran omo lowo lati dagba<br>*(Eating iron rich foods helps children develop)*						
8	Jije ounje ti o ni eroja eje ma nse iranlowo lati le fi okan si nkan<br>*(Consuming foods containing iron helps with concentration/focus)*						
9	Jije ounje ti o ni eroja eje maa nse iranlowo lati le ni okun ni ojumo<br>*(Consuming foods containing iron helps one have more energy during the day)*						
10	Jije ounje ti o ni eroja eje se iranlowo lati din oyi kikoni ku<br>*(Consuming foods containing iron can help reduce dizziness)*						

[READ THE QUESTION AND READ OUT THE OPTIONS. TICK RESPONSES PROVIDED BY RESPONDENT. **MULTIPLE CODING POSSIBLE**]

**8_K6.** Ewo ninu awon ounje wonyi ni o lero pe o kun fun eronja ti o n fun ni ni eje? *(Please select which of the foods described you think are good sources of iron?)*					UNAIDED
			**Beeni** *Yes (1)*	**Beeko** *No (2)*	**Mi o mo** *Don't know*
1	Eyin	*(Eggs)*			
2	Eja	*(Fish)*			
3	Eran pupa	*(Red meat)*			
4	Buredi ati iyefun	*(bread & flour)*			
5	Ewa	*(Beans)*			
6	Koro eso igi (furutu, kaju, awusa, epa ati bee bee lo)	*(Nuts (Ground nuts, Cashews, Peanuts, etc)*			
7	Ewebe alawo eweko (soko, ugu, tete, etc)	*(Green vegetables (Spinach, ugu, amaranth leaves, etc)*			
8	Koko, chocolati	*(Cocoa, Chocolate)*			
9	Akara oyinbo eleso ti eroja re pe	*(Rich fruit cake)*			
10	Woro irugbin (agbado ti a ti tun se si ounje oyinbo (Oatmeal), iresi, oka baba, ati bee bee lo etc)	*(Grains (Rice, Oatmeal, Millet, Maize, etc)*			
11	Ogede dudu	*(unripe plaintain)*			
12	Miliki	*(milk)*			
13	Oti stout, malti	*(stout, malt)*			

SECTION 9: DETERMINANTS OF BEHAVIOUR QUESTIONNAIRE

[INTERVIEWER, PLEASE READ OUT:]

"Ninu awon ibeere ti o kan a nife lati mo eroungba yin nipa obe sisie. A nife lati mo ero yin ni otito, ko si idahun ti o tona tabi to kuna. Jowo so eyi ti idahun yin ba je nipa lilo awon ohun ti a ti ko sile."

("IN THE NEXT FEW QUESTIONS WE ARE INTERESTED IN YOUR OPINION ABOUT COOKING STEWS. WE ARE INTERESTED IN YOUR HONEST OPINION, THERE ARE NO RIGHT OR WRONG ANSWERS. PLEASE INDICATE YOUR ANSWER USING THE OPTIONS BELOW EACH STATEMENT.")

[INTERVIEWER: DO NOT READ OUT THE HEADINGS IN BOLD CAPITALS, ONLY THE QUESTIONS]

KNORR BOUILLON CUBES

9_1. ATTITUDE

**9_1**	Fun emi, lilo koro Knorr nigba ti mo ba n se obe ata maa n je ki obe ata…<br>*For me, adding Knorr Bouillon cubes makes the stew…*				
a	**Ko wu oju ri rara**<br>*Very unpleasant (1)*	**Ko wu oju ri**<br>*Unpleasant (2)*	**Kosiyato**<br>*Not unpleasant, not pleasant (3)*	**O wu oju ri**<br>*Pleasant (4)*	**O wu oju ri daradara**<br>*Very pleasant (5)*
b	**O baje gaan**<br>*Very bad (1)*	**O baje**<br>*Bad (2)*	**Ko baje, ko dara**<br>*Not bad, not good (3)*	**O dara**<br>*Good (4)*	**O dara gan**<br>*Very good (5)*
b	**O fun ni ni ailera gaan**<br>*Very unhealthy (1)*	**O fun ni ni ailera**<br>*Unhealthy (2)*	**Kosiyato**<br>*Not unhealthy, not healthy (3)*	**O fun ni ni ilera**<br>*Healthy (4)*	**O fun ni ni ilera dara dara**<br>*Very healthy (5)*
d	**Ko te rara**<br>*Not at all tasty (1)*	**Ko te**<br>*Not so tasty (2)*	**Kosiyato**<br>*Neutral/undecided (3)*	**O te**<br>*Tasty (4)*	**O te gan**<br>*Very tasty (5)*
e	**Ko ma dun rara**<br>*Not at all delicious (1)*	**Ko ma dun**<br>*Not so delicious (2)*	**Kosiyato**<br>*Neutral/undecided (3)*	**Ko dun**<br>*Delicious (4)*	**Ko dun gan**<br>*Very delicious (5)*
f	**Ko ma ri ni lara rara**<br>*Not at all disgusting (1)*	**Ko ma ri ni lara**<br>*Not so disgusting (2)*	**Kosiyato**<br>*Neutral/undecided (3)*	**Ko ri ni lara**<br>*Disgusting (4)*	**Ko ri ni lara gan**<br>*Very disgusting (5)*

9_2. SOCIAL NORM (INJUNCTIVE/HUSBAND/FATHER)

[DO NOT ASK 9_2 IF MOTHER IS A WIDOW OR DIVORCED]

9_2a	Oko (baba) mi ro pe o ye ki n fi koro Knorr sinu obe ata.<br>*My husband/father thinks I should add Knorr Bouillon cubes to stews.*				
	**Mi o fara mo rara**<br>*Disagree Strongly (1)*	**Mi o faramo**<br>*Disagree (2)*	**Mo faramo/Mi o faramo**<br>*Neither agree/Nor disagree (3)*	**Mo faramo**<br>*Agree (4)*	**Mo faramo gan**<br>*Agree Strongly (5)*
b	Mo ro pe oko (baba) mi ro pe o ye ki n lo koro Knorr nigbatimo ban se obe ata.<br>*I think my husband/father expects me to use Knorr Bouillon cubes when cooking stews.*				
	**Mi o fara mo rara**<br>*Disagree Strongly (1)*	**Mi o faramo**<br>*Disagree (2)*	**Mo faramo/Mi o faramo**<br>*Neither agree/Nor disagree (3)*	**Mo faramo**<br>*Agree (4)*	**Mo faramo gan**<br>*Agree Strongly (5)*
c	Nigba ti n ba fi koro Knorr sinu obe ata mi, oko (baba) mi…<br>*When I add Knorr Bouillon cubes to my stew, my husband/father would.*				
	**Ko ni gba rara**<br>*Disapprove strongly (1)*	**Ko ni gba**<br>*Disapprove (2)*	**O le gba o, le ma gba**<br>*Neither approve nor disapprove (3)*	**Yi o gba**<br>*Approve (4)*	**Yi o gba gan**<br>*Approve strongly (5)*

9_3. SOCIAL NORM (INJUNCTIVE)

9_3a	Opolopo awon ti o se Pataki simi ro pe o ye kin fi koro Knorr si obe ata.<br>*Most people who are important to me think I should add Knorr Bouillon cubes to stews.*				
	**Mi o fara mo rara**<br>*Disagree Strongly (1)*	**Mi o faramo**<br>*Disagree (2)*	**Mo Faramo/Mi o faramo**<br>*Neither agree/Nor disagree (3)*	**Mo Faramo**<br>*Agree (4)*	**Mo Faramo gan**<br>*Agree Strongly (5)*
b	Mo ro pe awon toku ro pe o ye ki n lo koro Knorr ti mo ba n se obe ata.<br>*I think others expect me to use Knorr Bouillon cubes when cooking stews.*				
	**Mi o fara mo rara**<br>*Disagree Strongly (1)*	**Mi o faramo**<br>*Disagree (2)*	**Mo Faramo/Mi o faramo**<br>*Neither agree/Nor disagree (3)*	**Mo Faramo**<br>*Agree (4)*	**Mo Faramo gan**<br>*Agree Strongly (5)*
c	Nigbati mo ba fi koro Knorr si inu obe ata mi, awon eniyan ti eroungba won se iyebiye si mi …<br>*When I add Knorr Bouillon cubes to my stew, the people in my life whose opinions I value would…*				
	**Ko ni gba rara**<br>*Disapprove strongly (1)*	**Ko ni gba**<br>*Disapprove (2)*	**Ko ni gba/Gba**<br>*Neither approve nor disapprove (3)*	**Yi o gba**<br>*Approve (4)*	**Yi o gba gan**<br>*Approve strongly (5)*

9_4. *SOCIAL NORM (DEDUCTIVE)*

9_4a	Opolopo awon ti o se Pataki simi ni o ma n fi koro Knorr si obe ata.<br>*Most people who are important to me add Knorr Bouillon Cubes to stews.*				
	**Mi o fara mo rara**<br>*Disagree Strongly (1)*	**Mi o faramo**<br>*Disagree (2)*	**Mo Faramo/Mi o faramo**<br>*Neither agree/Nor disagree (3)*	**Mo Faramo**<br>*Agree (4)*	**Mo Faramo gan**<br>*Agree Strongly (5)*
b	Awon eniyan ti o wa ninu aye mi, ti erongba won se Pataki simi, maa n fi koro Knorr si obe ata won.<br>*The people in my life whose opinion I value, add Knorr Bouillon cubes to their stews.*				
	**Mi o fara mo rara**<br>*Disagree Strongly (1)*	**Mi o faramo**<br>*Disagree (2)*	**Mo Faramo/Mi o faramo**<br>*Neither agree/Nor disagree (3)*	**Mo Faramo**<br>*Agree (4)*	**Mo Faramo gan**<br>*Agree Strongly (5)*
c	Ninu agbegbe yin, eniyan melo ni e le so pe o n fi koro Knorr si obe ata won?<br>*In your community, how many people would you say add Knorr Bouillon cubes to their stews?*				
	**Won kere gan**<br>*Very few (1)*	**Won kere**<br>*Quite a few (2)*	**Won to ilaji**<br>*About half (3)*	**Won po**<br>*A lot (4)*	**Yi o fe je gbogbo won**<br>*Almost all (5)*

9_5. *PERCEIVED BEHAVIOURAL CONTROL*

9_5a	Fun emi, fifi koro Knorr si gbogbo obe ata ti mo n se …<br>*For me, adding Knorr Bouillon to every stews I cook would be…*				
	**Yi o Soro gan**<br>*Very difficult (1)*	**Yi o Soro**<br>*Difficult (2)*	**Kosoro/kororun**<br>*Not difficult, not easy (3)*	**O Rorun**<br>*Easy (4)*	**O Rorun pupo**<br>*Very easy (5)*
b	Ti o ba wu mi, mo le fi koro Knorr si gbogbo obe ata ti mo ba se.<br>*If I wanted to, I could add Knorr Bouillon cubes to every stew I cook.*				
	**Mi o fara mo rara**<br>*Disagree Strongly (1)*	**Mi o faramo**<br>*Disagree (2)*	**Mo Faramo/Mi o faramo**<br>*Neither agree/Nor disagree (3)*	**Mo Faramo**<br>*Agree (4)*	**Mo Faramo gan**<br>*Agree Strongly (5)*
c	Bawo ni o se wa lowo yin si lati fi koro Knorr si gbogbo obe ata ti e ba se?<br>*To what extent is it up to you to add Knorr Bouillon Cubes to the stews you cook?*				
	**Gbogbo re wa lowo elomiran**<br>*Completely up to others (1)*	**O wa lowo elomiran lopo igba**<br>*Mostly up to others 2*	**O wa laarin elomiran ati emi**<br>*Both up to others and up to me (3)*	**O wa lowo mi lopo igba**<br>*Mostly up to me (4)*	**O wa lowo emi nikan soso**<br>*Completely up to me (5)*

9_6. *HABIT*

9_6 a	Fifi koro Knorr si obe ata mi je ohun ti mo ma nse laironusi.<br>*Adding Knorr Bouillon cubes to my stews is something I do without thinking.*				
	**Mi o fara mo rara**<br>*Disagree Strongly (1)*	**Mi o faramo**<br>*Disagree (2)*	**Mo faramo/Mi o faramo**<br>*Neither agree/Nor disagree (3)*	**Mo Faramo**<br>*Agree (4)*	**Mo Faramo gan**<br>*Agree Strongly (5)*
b	Fifi koro Knorr si obe ata mi je ohun ti mo ma nse lai se pe mo ni lati ranti ki nto se.<br>*Adding Knorr Bouillon cubes to my stews is something I do without having to consciously remember.*				
	**Mi o fara mo rara**<br>*Disagree Strongly (1)*	**Mi o faramo**<br>*Disagree (2)*	**Mo faramo/Mi o faramo**<br>*Neither agree/Nor disagree (3)*	**Mo Faramo**<br>*Agree (4)*	**Mo Faramo gan**<br>*Agree Strongly (5)*
c	Fifi koro Knorr si obe ata mi je ohun ti ma ti bere si ise ki nto rip e mo nse.<br>*Adding Knorr Bouillon cubes to my stews is something I start doing before I realize I'm doing it.*				
	**Mi o fara mo rara**<br>*Disagree Strongly (1)*	**Mi o faramo**<br>*Disagree (2)*	**Mo faramo/Mi o faramo**<br>*Neither agree/Nor disagree (3)*	**Mo Faramo**<br>*Agree (4)*	**Mo Faramo gan**<br>*Agree Strongly (5)*

9_7. *INTENTION*

9_7a	Mo pero lati fi koro Knorr si obe ata mi ni awon osu ti o nbo.<br>*I intend to add Knorr Bouillon cubes to my stews in the next months.*				
	**Ko le sele rara**<br>*Extremely unlikely (1)*	**Ko le sele**<br>*Unlikely (2)*	**Kosiyato**<br>*Neither likely nor unlikely (3)*	**O le sele**<br>*Likely (4)*	**O le sele daada**<br>*Extremely likely (5)*
b	Mo pile lati fi koro Knorr si obe ata ni awon osu to nbo.<br>*I plan to add Knorr Bouillon cubes to my stews in the next months.*				
	**Mi o fara mo rara**<br>*Disagree Strongly (1)*	**Mi o faramo**<br>*Disagree (2)*	**Mo faramo/Mi o faramo**<br>*Neither agree/Nor disagree (3)*	**Mo Faramo**<br>*Agree (4)*	**Mo Faramo gan**<br>*Agree Strongly (5)*
c	Maa fi koro Knorr si obe ata ti ma se ni awon osu ti o n bo.<br>*I will add Knorr Bouillon cubes to the stews I make in the next months.*				
	**Mi o fara mo rara**<br>*Disagree Strongly (1)*	**Mi o faramo**<br>*Disagree (2)*	**Mo faramo/Mi o faramo**<br>*Neither agree/Nor disagree (3)*	**Mo Faramo**<br>*Agree (4)*	**Mo Faramo gan**<br>*Agree Strongly (5)*

GREEN LEAFY VEGETABLES

9_8. ATTITUDE

9_8	Fun mi, fifi ewebe (bi soko, tete, ati ugu) sinu obe ata mi ma nje ki obe ata mi....<br>*For me, adding green leafy vegetables (e.g., ugu, spinach, amaranth) to my stews, makes my stews …*				
a	**Ko ma wu oju ri rara**<br>*Very unpleasant (1)*	**Ko ma wu oju ri**<br>*Unpleasant (2)*	**Kosi yato**<br>*Not unpleasant, not pleasant (3)*	**Ko wu oju ri**<br>*Pleasant (4)*	**Ko wu oju ri daradara**<br>*Very pleasant (5)*
b	**Ko baje gaan**<br>*Very bad (1)*	**Ko baje**<br>*Bad (2)*	**Ko baje, ko dara**<br>*Not bad, not good (3)*	**Ko dara**<br>*Good (4)*	**Ko dara gan**<br>*Very good (5)*
c	**O fun ni ni ailera gaan**<br>*Very unhealthy (1)*	**O fun ni ni ailera**<br>*Unhealthy (2)*	**Kosiyato**<br>*Not unhealthy, not healthy (3)*	**o fun ni ni ilera**<br>*Healthy (4)*	**O fun ni ni ilera dara dara**<br>*Very healthy (5)*
d	**Ko te rara**<br>*Not at all tasty (1)*	**Ko te**<br>*Not so tasty (2)*	**Kosiyato**<br>*Neutral/undecided (3)*	**O te**<br>*Tasty (4)*	**O te gan**<br>*Very tasty (5)*
e	**Ko ma dun rara**<br>*Not at all delicious (1)*	**Ko ma dun**<br>*Not so delicious (2)*	**Kosiyato**<br>*Neutral/undecided (3)*	**Ko dun**<br>*Delicious (4)*	**Ko dun gan**<br>*Very delicious (5)*
f	**Ko ma ri ni lara rara**<br>*Not at all disgusting (1)*	**Ko ma ri ni lara**<br>*Not so disgusting (2)*	**Kosiyato**<br>*Neutral/undecided (3)*	**Ko ri ni lara**<br>*Disgusting (4)*	**Ko ri ni lara gan**<br>*Very disgusting (5)*

9_9. SOCIAL NORM (INJUNCTIVE/HUSBAND/FATHER)

[DO NOT ASK 9_9 IF MOTHER IS A WIDOW OR DIVORCED]

9_9a	Oko (baba) mi ro pe o ye ki n fi efo sinu obe ata mi.<br>*My husband/father thinks I should add green leafy vegetables to my stews.*				
	**Mi o fara mo rara**<br>*Disagree Strongly (1)*	**Mi o faramo**<br>*Disagree (2)*	**Mo faramo/Mi o faramo**<br>*Neither agree/Nor disagree (3)*	**Mo faramo**<br>*Agree (4)*	**Mo faramo gan**<br>*Agree Strongly (5)*
b	Oko (baba) mi ro pe o ye ki n fi efo sinu obe mi ti mo ban sebe ata.<br>*My husband/father expects me to add green leafy vegetables when cooking stews.*				
	**Mi o fara mo rara**<br>*Disagree Strongly (1)*	**Mi o faramo**<br>*Disagree (2)*	**Mo faramo/Mi o faramo**<br>*Neither agree/Nor disagree (3)*	**Mo Faramo**<br>*Agree (4)*	**Mo Faramo gan**<br>*Agree Strongly (5)*
c	Ti mo ba fi efo si obe ata mi, oko (baba) mi...<br>*When I add green leafy vegetables to my stew, my husband/father would.*				
	**Ko ni gba rara**<br>*Disapprove strongly (1)*	**Ko ni gba**<br>*Disapprove (2)*	**O le gba/o le ma gba**<br>*Neither approve nor disapprove (3)*	**Yi o gba**<br>*Approve (4)*	**Yi o gba gan**<br>*Approve strongly (5)*

9_10. *SOCIAL NORM (INJUNCTIVE)*

9_10a	Opolopo awon to se pataki simi ro pe o ye ki nfi efo sobe ata mi.<br>*Most people who are important to me think I should add green leafy vegetables to my stews.*				
	**Mi o fara mo rara**<br>*Disagree Strongly (1)*	**Mi o faramo**<br>*Disagree (2)*	**Mo faramo/Mi o faramo**<br>*Neither agree/Nor disagree (3)*	**Mo Faramo**<br>*Agree (4)*	**Mo Faramo gan**<br>*Agree Strongly (5)*
b	Mo ro pe awon eyan ro pe ki nfi efo si obe ti mo ba nse obe ata.<br>*I think others expect me to add green leafy vegetables when cooking stews.*				
	**Mi o fara mo rara**<br>*Disagree Strongly (1)*	**Mi o faramo**<br>*Disagree (2)*	**Mo faramo/Mi o faramo**<br>*Neither agree/Nor disagree (3)*	**Mo Faramo**<br>*Agree (4)*	**Mo Faramo gan**<br>*Agree Strongly (5)*
c	Ti mo ba fi efo si obe ata mi, awon to se pataki ninu aye mi…<br>*When I add green leafy vegetables to my stew, the people in my life whose opinions I value would…*				
	**Ko ni gba rara**<br>*Disapprove strongly (1)*	**Ko ni gba**<br>*Disapprove (2)*	**Ko ni gba/Gba**<br>*Neither approve nor disapprove (3)*	**Yi o gba**<br>*Approve (4)*	**Yi o gba gan**<br>*Approve strongly (5)*

9_11. *SOCIAL NORM (DEDUCTIVE)*

9_11a	Opolopo awon to se pataki si mi ma n fi efo sobe ata won.<br>*Most people who are important to me add green leafy vegetables to stews.*				
	**Mi o fara mo rara**<br>*Disagree Strongly (1)*	**Mi o faramo**<br>*Disagree (2)*	**Mo faramo/Mi o faramo**<br>*Neither agree/Nor disagree (3)*	**Mo faramo**<br>*Agree (4)*	**Mo faramo gan**<br>*Agree Strongly (5)*
b	Awon eniyan ti imoran won se pataki saye mi, ma n fi efo sobe ata won.<br>*The people in my life whose opinion I value, add green leafy vegetables to their stews.*				
	**Mi o fara mo rara**<br>*Disagree Strongly (1)*	**Mi o faramo**<br>*Disagree (2)*	**Mo faramo/Mi o faramo**<br>*Neither agree/Nor disagree (3)*	**Mo faramo**<br>*Agree (4)*	**Mo Fframo gan**<br>*Agree Strongly (5)*
c	Ninu agbegbe yin, eniyan melo ni e le so pe oun fi efo sobe ata won?<br>*In your community, how many people would you say add green leafy vegetables to their stews?*				
	**Won Kere gan**<br>*Very few (1)*	**Won kere**<br>*Quite a few (2)*	**Won to ilaji**<br>*About half (3)*	**Won po**<br>*A lot (4)*	**Yi o fe je gbogbo won**<br>*Almost all (5)*

9_12. *PERCEIVED BEHAVIOURAL CONTROL*

9_12a	Fun emi, fifi efo si gbogbo obe ata ti mo ba se…<br>*For me, adding green leafy vegetables to every stew I cook would be…*				
	**Yi o soro gan**<br>*Very difficult (1)*	**Yi o soro**<br>*Difficult (2)*	**Kosoro, kororun**<br>*Not difficult, not easy (3)*	**Yi o rorun**<br>*Easy (4)*	**Yi o rorun pupo**<br>*Very easy (5)*
b	Ti o ba wumi, mo le fi efo si gbogbo obe ata ti mo ba se.<br>*If I wanted to, I could add green leafy vegetables to every stew I cook.*				
	**Mi o fara mo rara**<br>*Disagree Strongly (1)*	**Mi o faramo**<br>*Disagree (2)*	**Mo Faramo/Mi o faramo**<br>*Neither agree/Nor disagree (3)*	**Mo Faramo**<br>*Agree (4)*	**Mo Faramo gan**<br>*Agree Strongly (5)*
c	Bawo ni o se wa lowo yin si lati fi efo si gbogbo obe ata ti e ban se?<br>*To what extent is it up to you to add green leafy vegetables to the stews you cook?*				
	**Gbogbo re wa lowo elomiran**<br>*Completely up to others (1)*	**O wa lowo elomiran lopo igba**<br>*Mostly up to others 2*	**O wa laarin elomiran ati emi**<br>*Both up to others and up to me (3)*	**O wa lowo mi lopo igba**<br>*Mostly up to me (4)*	**O wa lowo emi nikan soso**<br>*Completely up to me (5)*

9_13. *HABIT*

9_13a	Fifi efo sobe ata mi je ohun to ma n sele.<br>*Adding green leafy vegetablesto my stews is something I do without thinking.*				
	**Mi o fara mo rara**<br>*Disagree Strongly (1)*	**Mi o faramo**<br>*Disagree (2)*	**Mo faramo/Mi o faramo**<br>*Neither agree/Nor disagree (3)*	**Mo faramo**<br>*Agree (4)*	**Mo faramo gan**<br>*Agree Strongly (5)*
b	Fifi efo sobe ata mi je ohun ti mo ma nse lai gbiyanju lati ranti.<br>*Adding green leafy vegetables to my stews is something I do without having to consciously remember.*				
	**Mi o fara mo rara**<br>*Disagree Strongly (1)*	**Mi o faramo**<br>*Disagree (2)*	**Mo faramo/Mi o faramo**<br>*Neither agree/Nor disagree (3)*	**Mo faramo**<br>*Agree (4)*	**Mo faramo gan**<br>*Agree Strongly (5)*
c	Fifi efo sobe ata mi je ohun ti mo beere ki n to fiyesi pe mo ti n se.<br>*Adding green leafy vegetablesto my stews is something I start doing before I realize I'm doing it.*				
	**Mi o fara mo rara**<br>*Disagree Strongly (1)*	**Mi o faramo**<br>*Disagree (2)*	**Mo faramo/Mi o faramo**<br>*Neither agree/Nor disagree (3)*	**Mo faramo**<br>*Agree (4)*	**Mo faramo gan**<br>*Agree Strongly (5)*

9_14. *INTENTION*

9_14a	Mo pero lati fi efo sobe ata mi ni awon osu to n bo.<br>*I intend to add green leafy vegetables to my stews in the next months.*				
	**Ko le sele rara**<br>*Extremely unlikely (1)*	**Ko le sele**<br>*Unlikely (2)*	**Kosiyato**<br>*Neither likely nor unlikely (3)*	**O le sele**<br>*Likely (4)*	**O le sele daada**<br>*Extremely likely (5)*
b	Mo pinu lati fi efo sobe ata mi ni osu to n bo.<br>*I plan to add green leafy vegetables to my stews in the next months.*				
	**Mi o fara mo rara**<br>*Disagree Strongly (1)*	**Mi o faramo**<br>*Disagree (2)*	**Mo faramo/Mi o faramo**<br>*Neither agree/Nor disagree (3)*	**Mo faramo**<br>*Agree (4)*	**Mo faramo gan**<br>*Agree Strongly (5)*
c	Mo ma fi efo sobe ata mi ni awon osu to n bo.<br>*I will add green leafy vegetables to the stews I make in the next months.*				
	**Mi o fara mo rara**<br>*Disagree Strongly (1)*	**Mi o faramo**<br>*Disagree (2)*	**Mo faramo/Mi o faramo**<br>*Neither agree/Nor disagree (3)*	**Mo faramo**<br>*Agree (4)*	**Mo faramo gan**<br>*Agree Strongly (5)*

SECTION 10: MOTHER – DAUGHTER INTERACTION

[INTERVIEWER, PLEASE READ OUT EACH OF THE "ISSUES DISCUSSED" AND THEN READ OUT THE OPTIONS FROM "HARDLY EVER OR NEVER" TO "MORE THAN ONCE A DAY". NOTE THE RESPONSE OF THE RESPONDENT.]

**10.** Nje e le so fun wa bii igba melo ni e ma n ba omobinrin/iya yin se ajoro lori awon oro ti a ko sisale wonyi? *(Please can you indicate how often you discuss the following subjects with your daughter/mother?)*						
	**Ko to eekan losu tabi rara** *Less than once a month or never (1)*	**Eekan si emeta larin osu** *1 -3 times a month (2)*	**Eekan lose** *Once a week (3)*	**Eemeta si eemarun lose** *3-5 times a week (4)*	**Eekan lojumo** *Once a day (5)*	**Ju eekan lo lojumo** *More than once a day (6)*
Oye lori ina dida *(Cooking skills)*						
Didana ti o n fun ni ni ilera *(How to cook healthily)*						
Ooyi kiko ni, rire ara *(Feeling dizzy, tired)*						
Bi omobinrin yin (eyin) ti n se ni ile iwe *(How your daughter (you) is doing at school)*						
Ohun ti o ma n ba awon ore re jiroro le lori *(What she discusses with her friends)*						
Bi omobinrin yin (eyin) se le fi okan ba nkan lo si *(How well your daughter (you) can concentrate)*						
Iye agbara ti e ro pe e ni *(How much energy you feel you have)*						

SECTION 11: EVALUATION OF THE PROGRAMME

[ONLY FOR THE INTERVENTION GROUP IN IFE]

A nife lati bere awon ibeere lowo yin nipa erongba yin nipa eto "Daa, Roo, Run".
[WE WOULD LIKE TO ASK YOU A NUMBER OF QUESTIONS ABOUT YOUR THOUGHTS ON THE 'TOSS, STIR AND CRUMBLE' PROGRAMME.]

**11_1.** Awon eka wo ni e lero pe o se onigbowo eto "Daa, Roo, Run"? *What brands do you think sponsored the "Toss, Stir and Crumble" programme?)* [DO NOT PROMPT (SINGLE CODE)]		
1	Olupese koro Maggi *(Maggi)*	
2	Olupese koro Onga *(Onga)*	
3	Olupese koro Knorr *(Knorr)*	
4	Olupese koro Royco *(Royco)*	
5	Olupese koro Tasty *(Tasty Cubes)*	
6	Olupese Mr Chef *(Mr. Chef)*	
7	Kosi *(None)*	

[INTERVIEWER, WITH THE QUESTION 11_2, PLEASE SHOW THE LAMINATED CARD WITH A PICTURE OF THE "FOLLOW IN MY GREEN FOODSTEPS" PROGRAMME AND THE GOODY BAG".]

**11_2.** Nje e kopa ninu eto "Daa, Roo, Run" ni ile iwe omo yin obinrin" *(Did you participate in the "Toss, Stir and Crumble" programme at you daughter's school?*		
1	Beeni *(Yes)*	GO TO 11.3
2	Beeko *(No)*	GO TO 11.2A

[INTERVIEWER, PLEASE PUT THE LAMINATED CARD AWAY]

**11_2A.** Kini idi ti e ko le fi kopa ninu eto yi? *(What is the reason that you could not participate in the programme?)* [INTERVIEWER PLEASE NOTE RESPONSES AND **THEN END THE QUESTIONNAIRE: NO NEED TO CONTINUE**]

**11_3.** Nje e ti kopa ninu eto miran ti idanileko lori onje ati itoju ilera eni (bi iba, kokoro/arun edi, eto ilera mama ati omo) ni odun yi) *(Have you participated in any other programmes that educate on nutrition and health care (such as malaria, HIV/AIDS, maternal and child healthcare) this year?)*		
1	Beeni; E jowo e so eto wo ni e kopa ninu re *(Yes; please indicate which programmes you have participated in)* [INTERVIEWER PLEASE NOTE RESPONSES]	
2	Beeko *(No)*	

**11_4.** Ni odiwon ookan si eewa, ti ookan je pe e lero pe eto yen ko dara rara ati eewa je pe eto yen ko le dara ju be lo, bawo ni e e se se odiwon eto yi? *(On a scale from 1 to 10, with 1 indicating you felt the programme was very bad and 10 indicating you thought the programme really could not be better, how would you grade the programme?)*	

**11_5.** Kini e feran nipa eto yi? *(What did you like about the programme?)* [INTERVIEWER PLEASE NOTE RESPONSES]

**11_6.** Kini ohun ti e KO FERAN ninu eto na? (What did you NOT like about the programme?) [PLEASE NOTE RESPONSES]

**11_7.** Nje e ti bere si nso oro lori eto "Daa, Roo, Run" ti Knorr pelu omo/mama yin nipase eto yi? *(Did you start discussing the Knorr "Toss, Stir and Crumble" programme with your daughter/mother as a result of this programme?)*		
1	Beeni *(Yes)*	
2	Beeko *(No)*	

**11_8.**	Bawo ni e se nife tabi e ko nife si lati soro lori eto "Daa, Roo, Run" ti Knorr pelu omo yi obinrin/mama? *(To what extent did you like or dislike discussing the Knorr "Toss, Stir and Crumble" programme with you daughter/mother?)*			
**Mi o nife si rara** *Disliked it a lot (1)*	**Mi o nife si** *Disliked it (2)*	**Mo nife si/Mi o nife si** *Neither liked it nor disliked it (3)*	**Mo nife si** *Liked it (4)*	**Mo nife si gan** *Liked it a lot (5)*

**11_9.** Nje e ti bere si nso oro lori aito eroja iron (aito eje) ati awon ewu to ro mo pelu omo/mama yin nipase eto yi? *(Did you start discussing iron deficiency and its consequences with your daughter/mother as a result of this programme?)*		
1	Beeni *(Yes)*	
2	Beeko *(No)*	

11_10.	Bawo ni e se nife tabi e ko nife lati soro lori aito eroja iron (eroja eje) pelu omo yi obinrin/mama?<br>*(To what extent did you like or dislike discussing iron deficiency with your daughter/mother?)*			
**Mi o nife si rara**<br>*Disliked it a lot (1)*	**Mi o nife si**<br>*Disliked it (2)*	**Mo nife si/Mi o nife si**<br>*Neither liked it nor disliked it (3)*	**Mo nife si**<br>*Liked it (4)*	**Mo nife si gan**<br>*Liked it a lot (5)*

11_11.	Eto yi ti mu mi ma nronu si nipa aito eroja iron<br>*(The programme has made me think more about iron deficiency).*			
**Mi o fara mo rara**<br>*Disagree Strongly (1)*	**Mi o faramo**<br>*Disagree (2)*	**Mo faramo/Mi o faramo**<br>*Neither agree/Nor disagree (3)*	**Mo Faramo**<br>*Agree (4)*	**Mo Faramo gan**<br>*Agree Strongly (5)*

11_12.	Eto yi ti mu mi mo si bi mo ti se le se afikun eronja iron (eroja eje) ninu ounje mi<br>*(This programme has made me more aware of how I can increase the iron in my meals).*			
**Mi o fara mo rara**<br>*Disagree Strongly (1)*	**Mi o faramo**<br>*Disagree (2)*	**Mo faramo/Mi o faramo**<br>*Neither agree/Nor disagree (3)*	**Mo Faramo**<br>*Agree (4)*	**Mo Faramo gan**<br>*Agree Strongly (5)*

11_13.	Eto yi ti mu mi ni ajosepo to dan moran pelu omo mi obinrin/mama mi<br>*(This programme has made me interact with my daughter/mother in a more positive way).*			
**Mi o fara mo rara**<br>*Disagree Strongly (1)*	**Mi o faramo**<br>*Disagree (2)*	**Mo faramo/Mi o faramo**<br>*Neither agree/Nor disagree (3)*	**Mo Faramo**<br>*Agree (4)*	**Mo Faramo gan**<br>*Agree Strongly (5)*

(INTERVIEWER: ASK THE MOTHERS ONLY).

11_14.	Bawo lo se se si lati fi ilana ounje sise ti e ko lati eto “Daa, Roo, Run” ti Knorr le omo yin obinrin lowo pelu erongba pe yi o lo irufe ilana bayi pelu idile re ni ojo iwaju<br>*(How likely is it that you will pass on the recipes from the Knorr “TOSS, STIR AND CRUMBLE” programme on to your daughter(s) with the intention of her using that recipe with her family in the future?)*			
**Ko le sele rara**<br>*Extremely unlikely (1)*	**Ko le sele**<br>*Unlikely (2)*	**Kosiyato**<br>*Neither likely nor unlikely (3)*	**O le sele**<br>*Likely (4)*	**O le sele daada**<br>*Extremely likely (5)*

[INTERVIEWER: ASK THE DAUGHTERS ONLY]

11_15.	Bawo lo se se si pe o lo ilana ounje eto “Daa, Roo, Run” ti Knorr ti mama re ti o ba n se ounje fun idile tire ni ojo iwaju?<br>*(How likely is it that you will use your mother’s Knorr “Toss, Stir and Crumble” programme recipes when cooking for your own family in the future?)*			
**Ko le sele rara**<br>*Extremely unlikely (1)*	**Ko le sele**<br>*Unlikely (2)*	**Kosiyato**<br>*Neither likely nor unlikely (3)*	**O le sele**<br>*Likely (4)*	**O le sele daada**<br>*Extremely likely (5)*

(INTERVIEWER: ASK THE MOTHERS ONLY).

**11_16.**	Bawo lo ti se pataki si fun yin lati fi ilana ounje sise yin le omo lowo ki o le mulo ni ojo iwaju?<br>*(How important is it for you to pass down your cooking skills/style to your daughter so she uses this in the future?).*			
**Ko se Pataki rara**<br>*Very unimportant (1)*	**Ko se pataki**<br>*Unimportant (2)*	**O se Pataki/Ko s e pataki**<br>*Neither important nor Unimportant (3)*	**O se pataki**<br>*Important (4)*	**O se Pataki gan**<br>*Extremely Important (5)*

**11_17.**	Bawo lo ti se se si fun yin lati ma fi ewebe sinu obe ata ti e ngbe fun awon molebi yin ni ojo iwaju?<br>*(How likely is it that you will include leafy green vegetables in stews that you serve your family in the future?)*			
**Ko le sele rara**<br>*Extremely unlikely (1)*	**Ko le sele**<br>*Unlikely (2)*	**Kosiyato**<br>*Neither likely nor unlikely (3)*	**O le sele**<br>*Likely (4)*	**O le sele dada**<br>*Extremely likely (5)*

**11_18.**	Lasiko ati leyin eto “Daa, Roo, Run” ti Knorr, li lo isebe onikoro knorr si obe ata mi....<br>*(During and after the Knorr “TOSS, STIR AND CRUMBLE” programme, I have started using Knorr Bouillon cubes in my stews…)*			
**Ti din kun gan**<br>*A lot less (1)*	**Ti din kun**<br>*A bit less (2)*	**Bakanna ni**<br>*The same (3)*	**O le si**<br>*A bit more (4)*	**O le si gan**<br>*A lot more (5)*

**11_19.**	Lasiko ati leyin eto “Daa, Roo, Run” ti Knorr mo ti bere si nfi ewebe si obe ata mi....<br>*(During and after the Knorr “Toss, Stir and Crumble” programme, I have started adding leafy green vegetables to my stews…)*				
**Mi o fi si rara**<br>*Not at all (1)*	**Leekan larin ose meji**<br>*(Once every two weeks)*	**Leekan lose**<br>*(Once every week)*	**Leemeji lose**<br>*(Twice a week)*	**Lemeta lose**<br>*(Three times a week)*	**Lemerin lose**<br>*(Four times a week)*

**11.20** Nje e kopa ninu ibere eto “Daa, Roo, Run” ti Knorr ti o waye ni ile iwe omo obirin yin?<br>*(Did you participate in the Knorr “Toss, Stir and Crumble” School induction event at your daughter’s school?)*		
1	Beeni *(Yes)*	
2	Beeko *(No)*	

**11.21**. Nje e gba apo ti o ni awon nkan meremere ninu ni ibere eto?<br>*(Did you receive a goody bag at the induction event?)*<br>[INTERVIEWER, PLEASE SHOW THE LAMINATED CARD WITH THE GOODY BAG]		
1	Beeni *(Yes)*	
2	Beeko *(No)*	

11.22 Nje apo nkan meremere yi ni awon nkan wonyi ninu?
(Did the goody bag contain the following items?)

**11.22**.A. Isebe koro knorr?<br>*(Knorr Bouillon cubes?)*					
1	Beeni *(Yes)*		➔ A1. Nje e lo?<br>*Did you use them?*	Beeni *(Yes)*	
2	Beeko *(No)*			Beeko *(No)*	

**11.22.B.** Idi ewebe ugu kan?<br>*(A bunch of ugu leaves?)*					
1	Beeni *(Yes)*		➔ B1. Nje e lo?<br>*Did you use them?*	Beeni *(Yes)*	
2	Beeko *(No)*			Beeko *(No)*	

**11.22.C.** A ko sile nkan ti a fe ra?<br>*(A shopping list?)*

1	Beeni *(Yes)*		➔ C1. Nje e lo? *Did you use it?*	Beeni *(Yes)*	
2	Beeko *(No)*			Beeko *(No)*	

**11.22.D**. Kalenda obe ata olose mefa? *(6-week stew calender?)*					
1	Beeni *(Yes)*		➔ D1. Nje e lo? *Did you use it?*	Beeni *(Yes)*	
2	Beeko *(No)*			Beeko *(No)*	

**11.22.E**. Ohun amin lati fi mo igba ti e fi ewebe si obe ata yin? *(Stickers to track when you made stews with green leafy vegetabes?)*					
1	Beeni *(Yes)*		➔ E1. Nje e lo? *Did you use them?*	Beeni *(Yes)*	
2	Beeko *(No)*			Beeko *(No)*	

**11.22.F**. Leta ileri? *(The promise letter?)*					
1	Beeni *(Yes)*		➔ F1. Nje e koo? *Did you write it?*	Beeni *(Yes)*	
2	Beeko *(No)*			Beeko *(No)*	

**11.23**. Nje e lo kalenda lati fi mo igba ti e se obe ata pelu ewebe? *(Did you use the calender to track when you made stews with green leafy vegetables?)*		
1	Beeni *(Yes)*	
2	Beeko *(No)*	

**11.24**. Nje e ri oun to jo mo eto "Daa, Roo, Run" ti Knorr ni ile itaja to wa ni agbegbe yin? *(Did you see Knorr "Toss, Stir and Crumble" programme materials at the shops in your community?)*		
1	Beeni *(Yes)*	
2	Beeko *(No)*	

**11.25**. Melo ninu ere itage pelu Omotola ni e feti si? *(How many drama episodes with Omotola did you listen to?)*		
	(Please indicate number of radio episodes)	

**11.26**. Ewo Ninu Awon Eto Olugbe Ti eto "Daa, Roo, Run" ti Knorr ni e kopa ninu re? *(Which Resident Events of the Knorr "Toss, Stir and Crumble programme did you particpate in?)* [INTERVIEWER, PLEASE TICK APPROPRIATE BOX]		
	Eto olugbe akoko *(First resident event)*	
	Eto olugbe ekeji *(Second resident event)*	

**11.27**. Igba melo ni asoju knorr de ile yin lati soro nipa eto "Daa, Roo, Run" ti Knorr? *(How many times did Brand Ambassadors visit your home to talk about the Knorr "Toss, Stir and Crumble" programme?)*		
	(Please indicate number of visits)	

**11.28**. Nje e fi nomba ero ibanisoro yin ranse fun eto atejise alagbeka? *(Did you send in your phone-number for the mobile messaging service?)*		
1	Beeni *(Yes)*	
2	Beeko *(No)*	

**11.29**. Nje e gba awon atejise kankan lori ero ibara eni soro alagbaka yin? *(Did you receive any text messages on your mobile phone?)*

1	Beeni *(Yes)*		➔ 11.28.1. Atejise melo ni e ri gba? *How many texts did you receive?*		*(Number)*
2	Beeko *(No)*				

**11.30.** Nje e mo wipe e le je ankara (iya) tabi apo ile-iwe (omo binrin) latari kikopa sansan ninu eto na nigbayan? *(Did you know that you could win an ankara (mothers) or school bag (daughters) by actively participating in the programme then?)*		
1	Beeni *(Yes)*	
2	Beeko *(No)*	

11_31.

E jowo nje e le se itokasi bi e se korira/feran awon eto ti o wa ninu eto "Daa, Roo, Run" ti Knorr *Please can you indicate how much you (dis)liked the elements in the Knorr "Toss, Stir and Crumble" programme)* [INTERVIEWER READ OPTIONS BELOW AND READ OUT OPTIONS FOR ANSWERS ON THE LEFT]		**Mi o nife si rara** *Disliked it a lot (1)*	**Mo ni fe si** *Disliked it (2)*	**Mo fe tabi mi o ni fe si** *Neither liked it nor disliked it (3)*	**Mo ni fe si** *Liked it (4)*	**Mo ni fe si gan** *Liked it a lot (5)*
1	Apo nkan meremere *(Goody bag)*					
2	Isebe koro knorr ofe *(Free samples of Knorr Bouillon)*					
3	Leta ileri *(Promise letter)*					
4	Iwe nkan ti a fe ra *(Shopping list)*					
5	Oluran ni leti ohun ti o wa fun rira ni ile itaja *(Shopping reminder in the shops)*					
6	Alemo pelebe fun irani leti *(Reminder sticker)*					
7	Isafinhan bi a ti se ndana ni ipele akoko *(Cooking demonstration at the first event)*					
8	Idije kekrere lasiko ipele ti ile iwe *(Quiz during the school events)*					
9	Ere lori redio *(Radio drama)*					
10	Omotola ati odomobinrin re *(Omotola and daughter)*					
11	Ori ati Ijo pelu Yemi Alade *(Song & Dance with Yemi Alade)*					
12	Kalenda oluranti *(Reminder calendar)*					
13	Atejise alagbeka *(Mobile messages)*					
14	Anfani lati je aso ankara *(Chance to win an Ankara)*					
15	Anfani lati je apo ile iwe *(Chance to win a school bag)*					

**11_32**	Bawo le ti se ri ipa ti Knorr nko ninu eto "Daa, Roo, Run" ti Knorr si? *How do you feel about the role that Knorr plays in the Knorr "Toss, Stir and Crumble" programme?*			
**Mi o ni fe si rara** *Disliked it a lot (1)*	**Mi o ni fe si** *Disliked it (2)*	**Mo ni fe si, mi o ni fe si** *Neither liked it nor disliked it (3)*	**Mo ni fe si** *Liked it (4)*	**Mo ni fe si gan** *Liked it a lot (5)*

11_33. Ni bayii ti o ti kopa ninu eto naa, bawo ni o se fara mo tabi lodi si awon gbolohun wonyi?

Having participated in the programme, to what extent do you agree or disagree with the following statements: [INTERVIEWER READ OPTIONS BELOW AND READ OUT OPTIONS FOR ANSWERS ON THE LEFT]		**Mi o fara mo rara** *Disagree Strongly (1)*	**Mi o faramo** *Disagree (2)*	**Mo faramo/Mi o faramo** *Neither agree/Nor disagree (3)*	**Mo Faramo** *Agree (4)*	**Mo Faramo gan** *Agree Strongly (5)*
1	Mo fokan tan eya eroja Knorr *(I trust the Knorr brand)*					
2	Mo ri eroja Knorr gege bi eyi to n fun ni ounje asaralore *(I associate Knorr with nutritious cooking)*					
3	Mo gbagbo pe eroja Knorr je ojulowo ti o le gbe adun ounje re jade *(I believe Knorr are experts in helping you bring out the flavours of your dishes)*					
4	Eroja Knorr n ran mi lowo lati pese ounje aladun ti o tayo *(Knorr helps me create great tasting meals)*					
5	Eroja Knorr maa n fun mi ni iwuri lati se awon ounje tuntun pelu ogbon ati imo perete *(Knorr inspires me to cook new dishes with simple smart meal ideas)*					
6	Mo gbagbo pe eroja Knorr kun fun awon eroja abinibi *(I believe Knorr contain natural ingredients)*					
7	Knorr je orisun eroja iron ti o peye ninu ounje mi *(Knorr is a good source of iron in my dishes)*					

**11_34.** Bawo ni eto “Daa, Roo, Run” ti Knorr se ri si lara re lori eya naa? *(How does the Knorr ‘Toss, Stir and Crumble ’ programme, make you feel about the brand?)* *[ONE OPTION ONLY]*		
1	O je ki n feran eya naa gaan *(It makes me like the brand a lot)*	
2	O je ki n feran eya naa *(It makes me like the brand)*	
3	Emi ko nife si eya naa. Bee ni n ko si korira re *(I am neutral about the brand)*	
4	O je ki n korira eya naa *(It makes me dislike the brand)*	

**11_35**	Bawo lo ti se se si wipe e ma ra koro Knorr ni ojo iwaju ? *How likely is it that you will buy Knorr stock cubes in the future?*			
**Ko le sele rara** *Extremely unlikely (1)*	**Ko le sele** *Unlikely (2)*	**O le sele, ko le sele** *Neither likely nor unlikely (3)*	**O le sele** *Likely (4)*	**O le sele daada** *Extremely likely (5)*

**11_36**	Bawo lo se se e se fun yin lati rohin koro Knorr fun awon ore ati molebi yin nigba ti e ti kopa ninu eto “Daa, Roo, Run” ti Knorr? *How likely are you to recommend Knorr stock cubes to friends and family having participated in the Knorr “Toss, Stir and Crumble” campaign?*			
**Ko le sele rara** *Extremely unlikely (1)*	**Ko le sele** *Unlikely (2)*	**Ko si ya to** *Neither likely nor unlikely (3)*	**O le sele** *Likely (4)*	**O le sele daada** *Extremely likely (5)*

[INTERVIEWER: ASK THE DAUGHTERS ONLY]

11_37	Bawo lo ti se se si fun e lati so fun mama re ki won lo koro Knorr nigbati won ba ndana ni ojo iwaju?<br>*How likely are you to ask you mum to use Knorr stock cubes in the future when she cooks?*			
**Ko le sele rara**<br>*Extremely unlikely (1)*	**Ko le sele**<br>*Unlikely (2)*	**Ko si ya to**<br>*Neither likely nor unlikely (3)*	**O le sele**<br>*Likely (4)*	**O le sele daada**<br>*Extremely likely (5)*

[INTERVIEWER: ASK THE DAUGHTERS ONLY]

11_38	Bawo lo ti se se si fun e lati lo isebe onikoro knorr ni ojo iwaju ti o ba ndana?<br>*How likely are you to use Knorr stock cubes in future when you cook?*			
**Ko le sele rara**<br>*Extremely unlikely (1)*	**Ko le sele**<br>*Unlikely (2)*	**Ko si ya to**<br>*Neither likely nor unlikely (3)*	**O le sele**<br>*Likely (4)*	**O le sele daada**<br>*Extremely likely (5)*

11_39	Ni eyin igba ti mo ti kopa ninu eto eto “Daa, Roo, Run” ti Knorr mo ma nri wipe mo nfi koro knorr ati ewebe si obe ata mi ni igba gbogbo<br>*After participating in the Knorr “Toss, Stir and Crumble” programme, I make sure that I always add both Knorr Bouillon cubes and green leafy vegetables to my stews.*			
**Mi o fara mo rara**<br>*Disagree Strongly (1)*	**Mi o faramo**<br>*Disagree (2)*	**Mo faramo/Mi o faramo**<br>*Neither agree/Nor disagree (3)*	**Mo Faramo**<br>*Agree (4)*	**Mo Faramo gan**<br>*Agree Strongly (5)*

TIME: ENDED: ____/____/ (HH/MM)

E SEUN FUN IDAHUN ATI ASIKO YIN

THANK YOU FOR YOUR ANSWERS AND THE TIME SPENT WITH ME
