# Supplementary material for: The effect of the “Follow in my Green Food Steps” programme on cooking behaviours for improved iron intake: a quasi-experimental randomized community study
Source: Int J Behav Nutr Phys Act. 2018 Aug 16;15:79. doi: 10.1186/s12966-018-0710-4 (PMC6097342; doi:10.1186/s12966-018-0710-4)
Supplement: Supplementary file 5 — Revised Protocol for Follow in my Green Food Steps Behaviour Change Impact study. (PDF 1759 kb) [file 12966_2018_710_MOESM5_ESM.pdf]

Study Title

Pilot Follow in my Food Steps Behaviour Change Impact Study

Reference Number: SPB01_2015

Nigerian Team: Dr. Oyedunni Arulogun B.Ed, MEd, MPH, PhD, FRSPH
Principal Investigator
University of Ibadan,
oyedunniarulogun@gmail.com
Mob: +234-803-579-4630
Mr. M. A. Titiloye BSc, MPH– Co-Investigator
University of Ibadan,
msbnsmtiti@yahoo.com
Mob: +234-708-214-4351

International Team: Dr. René Lion, PhD, Senior Consumer Scientist
International Principal Investigator
Unilever R&D Vlaardingen
PO Box 114 3130 AC Vlaardingen
Tel: +31-(0)10- 460 6116
Mob: +31-(0)6-53758503
E-mail: rene.lion@unilever.com

Signature:

Summary

Ethical clearance is requested for a study investigating the effectiveness of a behaviour change intervention aimed at increasing the intake of green leafy vegetables and iron-fortified Knorr Bouillon cubes in Nigerian participants. In a quasi-experimental design with 2 intervention communities and 2 control communities that are matched on demographics, the impact of the intervention is measured at a household level by means of self-reported intake of Knorr iron-fortified cubes and green leafy vegetables, and at an individual level in changes in behavioural determinants. From each community, 100 households, i.e., 100 mothers and 100 daughters will be recruited, for a total of 800 respondents (200 mothers and 200 daughters for the intervention group, 200 mothers and 200 daughters for the control group). Furthermore, we will explore whether the programme leads mothers and daughters to discuss iron and anaemia and the steps that can be taken to increase iron intake.

Background and introduction

Anemia is considered the most prevalent nutritional deficiency globally. It affects 1.62 billion people, which corresponds to 24.8% of the population. The highest prevalence is in preschool-age children (47.4%) and the lowest prevalence is in men (12.7%). The population group with the greatest number of individuals affected is non-pregnant women (468.4 million) (WHO, 2008), of which roughly half is due to iron deficiency (Zimmerman & Hurrell, 2007). Nutritional iron deficiency has been identified as one of the ten leading factors for disease, disability and death (WHO, 2002). Prevalence of anaemia appears to be highest in Central and West Africa, where approximately half of the women have anaemia (Stevens et al., 2013).

Three approaches have been proposed to correct nutritional iron deficiency: modifying the diet to improve the nutritional value and iron bio-availability, supplementation (e.g., through pills) and fortification (i.e., the addition of micronutrients to processed foods)(Lynch, 2005). Of these, supplementation is the least preferred option for long-term supplementation, as it is relatively expensive, requires close monitoring and lack of compliance is a key limitation of supplementation programmes (Lynch, 2005). Dietary modification is the preferred method, although changing dietary behaviours is challenging and can have practical limitations, such as the availability of iron-rich foods. In practice, fortification is considered to be a practical and cost-effective long-term solution (Lynch, 2005; WHO, 2004). Furthermore, bouillon cubes can be a good micronutrient fortification vehicle, as it is widely used in cooking and micronutrients included in the bouillon cubes will reach the whole family when the family members consume the dishes prepared with the bouillon cubes.

As part of Knorr's Social mission, Knorr has set itself the goal to create a social mission programme around nutritious cooking. The social mission programme combines the two preferred methods of improving dietary intake of iron, i.e., modification of the diet and fortification of processed foods. Specifically, it aims to increase the nutritional value of people's diets by getting consumers to:

- add green, leafy vegetables
- use Knorr iron-fortified Bouillon Cubes

The programme will be piloted it Nigeria, before being rolled out further in Nigeria and the rest of Africa. Small scale studies in Southern Nigeria have indicated a large range of iron intake, ranging from 10-32mg/d (Anyika et al., 2009; Ekpo & Eob, 2006; Glew et al., 2001; Otemuyiwa & Adewusi, 2012), with a significant proportion not meeting the recommended daily intake of 20mg/d (FAO/WHO). Furthermore, a larger intake study conducted in 2008 suggested that Nigerian households do not have much variation in the diet and that micronutrient intake (including iron) is low (Sanusi, 2010). This suggests that a behaviour change programme should be able help more Nigerian women to reach the recommended daily intake of iron.

This protocol describes a study to evaluate the effectiveness of the programme in getting consumers to add green, leafy vegetables and use Knorr iron-fortified bouillon cubes by conducting a quasi-experimental design, with pre-and post-intervention measures to assess the impact.Specifically, the objective is to assess the impact of the programme on food intake behaviours as well as the determinants of behaviour that are assumed to mediate this change (Ajzen, 2012; Ajzen& Cote, 2008; Webb & Sheeran, 2006). Furthermore, we will explore whether the intervention has led mothers and daughters to discuss lack of iron and how cooking skills (e.g. adding green leafy vegetables at the end) can help alleviate this, as well as self-reported well-being and health.

Objectives

Main objective

1. To assess the effectiveness of the Behaviour Change campaign versus no intervention in changing behaviours in a real-life setting using self-reported measures of
    a. Knorr Fortified bouillon cubes use
    b. The addition of green leafy vegetables (specifically pumpkin leaves) to two stews per week

Secondary objectives

2. To establish whether changes in behaviour can be explained by changes in behavioural determinants (e.g., attitudes towards using iron-fortified Knorr cubes and adding green leafy vegetables).
3. To assess the extent to which mothers and daughters discuss iron enriched food and food preparation.

Location

The participants involved in the intervention leg of the study will be recruited in the South-East and West of Nigeria. Ijebu-Ode in Ogun State and Amaigbo, in Imo State, Nigeria constitute the intervention sites.The participants involved in the control leg of the study will be recruited from Osogbo, in Osun state and Umundu in Enugu state.

Participants

The intervention focuses on mothers (the main cooks) and the oldest daughter between 12 and 16 years (who assists in the cooking) in order to impact the food intake of the whole household.
In each community, 100 mothers and 100 daughters will be recruited, for a total of 400 mothers and 400 daughters: 400 mothers and daughters in the intervention group and 400 in the control group.

For each household, the mother and the eldest teenage daughter will be asked to answer all the questions regarding food intake together, and questions pertaining to the behavioural determinants, the interaction between mother and daughter and some additional questions regarding general well-being and health separately.

Participants will be informed that they can stop participating at any point during the study, without the obligation to provide a reason for stopping. They will also be informed that if either of the participants (the mother or the eldest daughter) withdraws from the study, the other will also be withdrawn from the study.

Inclusion Criteria

- Families with an eldest daughter between 12-16 years old living in the same house
- Fluency in the Nigerian language relevant to the region in which the study is conducted.
- Ability to read (either in the local language or English)

Exclusion Criteria

- Families where one of the family members has specific dietary restrictions (e.g., seafood intolerance)
- Working for a market research agency, advertising, home and personal care industry, or professionally involved with foods (e.g., working in the food business).

Methodology

Study material

Food intake measures

The primary objective of the "Follow in my Green Food Steps" programme is to ensure that those exposed to the programme have increased their intake of green leafy vegetables and iron-fortified Knorr cubes. The primary measure of interest therefore focuses on these key behaviours. The gold standard in food intake measures is the 24-hour dietary recall, which allows the calculation of nutrient intakes. These measures are intended to assess food intake at the individual level, which is not the main interest of this study. When conducted over two days in the week and with sufficient respondents, a 24-hour dietary recall can provide a good insight into the group mean intake, and should be able to pick up differences between the intervention and the control group. However, a drawback is that this methodology requires considerable time and effort, both from the respondents and the research team.

An alternative methodology is to specifically assess how often the target behaviours (using iron fortified cubes, adding leafy green vegetables) have been performed over the past weeks by using a Short Food Frequency questionnaires methodology (see e.g., (Bogers, 2004). The advantages of such

a short Food Frequency Questionnaire is that it is relatively easy to administer and more specific with respect to the target behaviours we are interested in. The questionnaire used in this study is therefore based on the short Food Frequency methodology and focuses on the two dish categories that are most prepared in Nigeria, stews and soups. For each of these categories, respondents are asked to indicate how often they have prepared these. Furthermore, for each of the dishes they have prepared, they are asked to indicate from a set list of ingredients how much of these ingredients they have used. The list covers the target ingredients (bouillon cubes and green leafy vegetables) as well as the main other vegetable and protein sources typically used in these dishes. By focusing on the specific target dishes, the fact that it is difficult for people to recall what they have cooked in the past few weeks will be circumvented.

Determinants of behaviour

One of the most widely used models in explaining and predicting behaviour and behaviour change is the Theory of Planned Behaviour (TPB; Ajzen, 1991, 2011, 2012; Ajzen & Gilbert-Cote, 2008; McEachan, Conner, Taylor, & Lawton, 2011; Sniehotta, Presseau, & Araujo-Soares, 2014). It has proven to be a parsimonious and useful model to explain the main determinants of behaviour and identify key targets for developing behaviour change interventions. Furthermore, over the past decades, it has become increasingly clear how the constructs defined in the model should be operationalised and measured (Ajzen, 2001), and when applied in a theory-consistent way, can deliver encouraging results (Ajzen, 2014; Webb, Joseph, Yardley, & Michie, 2010; Webb & Sheeran, 2006). The TPB postulates that the attitude, social norm and perceived behavioural control with respect to the target behaviour are the key determinants of the intention to perform a behaviour, which in turn is the key determinant of behaviour. The influence of other (distal) factors, such as gender, context etcetera is mediated through these three constructs. Of course, in a general model like this, specific determinants pertinent to this model can be added. Recent theorizing suggests that "habit" is an additional key determinant that can explain behaviour over and above the other key determinants of behaviour (Ajzen, 2011; de Bruijn, 2010; de Bruijn et al., 2007; Gardner, de Bruijn, & Lally, 2011).

One under-investigated aspect in the study of the Theory of Planned Behaviour and behaviour change is the assumption that changes in the determinants of behaviour (attitudes, social norm and perceived behavioural control) are associated with changes in intention and subsequently behaviour (Hardeman et al., 2002; Sainsbury, Mullan, & Sharpe, 2015; Sniehotta et al., 2014). Therefore, the inclusion of measures to assess key determinants of behaviour both at baseline and after the intervention, can help evaluate the impact of the behaviour change intervention on these key determinants, as well as to what extent they are related to changes in the key associated behaviours.

The key factors to be measured for this study are 1) knowledge about iron, i.e., the understanding that a lack of iron is associated with tiredness, dizziness, lack of concentration and that consuming iron-fortified cubes as well as green leafy vegetables is a way to alleviate this issue; 2) attitudes towards the key behaviours (using Knorr fortified cubes and consuming green leafy vegetables); 3) the social norm towards these behaviours; 4) self-efficacy with respect to cooking the "Follow in my

Green Food Steps" way; 5) to what extent the target behaviours are habits. All these variables are measured both before the intervention as well as after the intervention. The full questionnaire can be found in appendix 1.

Measure of mother-daughter interaction
The mother-daughter interaction in the programme is used as a means to communicate the key insights around iron and anaemia.Therefore, the study will measure the extent to which the programme increased the likelihood that mothers and daughters discussed food preparation and iron-deficiency.

Study procedure
The programme will be delivered by EXP, a social marketing agency based in Nigeria. The ambition is to enrol 50,000 participants (or 25,000 households) in the initial behaviour change programme. Half of these will be enrolled in Ijebu-Ode (Ogun State) and the other half will be enrolled in Amaigbo (Imo State).

Two hundred households (100 per community) out of these 25,000 householdswill be recruited by EXP to take part in the impact evaluation study. An additional 200 households will be recruited in the two control communities Osogbo, in Osun state and Umundu in Enugu state by Ibadan University.EXP will recruit participants for the "Follow in my Green Food Steps" programme through town-hall meetings, meetings of Women associations, school sessions and door-to-door recruitment. EXP will also pre-recruit participants for the study from the door-to-door recruitment activities. Whilst going from door to door, EXP will also ask if the participants are willing to take part in a study. The overall objectives of the study will be explained in such a way that it is not apparent that the study is linked to the evaluation of the programme. If they agree, their contact details will be noted and these will be handed over to the Principal Investigator (PI). It will be made clear that agreeing to pass on contact details does not imply in any way that they are obliged to take part in the study.

Interviewers from the PI's team will then contact these households, explain again the purpose of the study and hand over an informed consent form for them to read at their own pace. Once the informed consent form has been signed, the interviewer will conduct the baseline interview. The informed consent form will not explicitly mention the relation between the intervention programme and the study, as this might impact the results (experimenter demand).

Before the commencement of data collection, the following activities will be implemented.

1. *Recruitment of supervisors and Interviewers:* Efforts will be made to select and recruit experienced interviewers who had at least National diplomas certificate while the supervisors will be Master of Public Health graduates.
2. *Enhancing tool reliability:* Training fieldwork manual will be developed. The English version of the survey will be translated to the local language (Yoruba and Igbo) and back translated to English to ensure accuracy.

3. *Training of Study Personnel and Field testing of Tools*: All research assistants/supervisors will be trained for five days before the field testing of instrument and prior to baseline data collection. Refresher training will be done for 3 days before post- intervention data collection. Field testing of the instrument for data collection will be made to ensure validity and reliability.
4. *Purchase of Project equipment and materials*: Interviewers and supervisors' data collection kits will be purchased for use by the team before data collection.

After the pre-field activities, baseline data will be collected for a total period of 4 weeks with continuous supervision of all the research assistants. Completed questionnaire will be collected in hard copy and then entered into computers at a central location. Details of the project activities and time allotted are shown under the "Timeline" below.

Timeline of Activities

**Activities**	**June '15**	**July '15**	**Aug '15**	**Sept. '15**	**Oct. '15**	**Nov. '15**	**Dec. '15**	**Jan. '16**	**Feb '16**
Finalisation and signing of Contract	■								
Finalisation of the Protocol		■							
Protocol submission for Ethical Approval		■							
Recruitment and Training/Pre-testing			■						
Baseline Data Collection			■	■					
Baseline Data Analysis				■					
Intervention				■	■	■			
Recruitment and Re-Training of interviewers						■			
Post-Intervention Data Collection							■		
Data Analysis							■	■	
Report Writing							■	■	
Submission of Draft report								■	
Submission of Final report									■

Once recruited, a trained interviewer will contact the participants for an interview. At this interview, the participants will be asked to sign an informed consent (see appendix 8) and the baseline interview will be conducted.

At baseline the interviewer will ask the mother a number of key demographic questions, where they shop, the availability of key ingredients, how often they cook and what devices and utensils they have available to do the cooking(see appendix 2).The interviewer will also ask for permission to make a picture of the kitchen. The daughter will only be asked her age.
Then, they will interview the mother and the daughter together regarding their food intake with respect to the key dishes and relevant ingredients (e.g., the specific vegetables targeted in the programme). They will then be separately interviewed with regards to the behavioural determinants, knowledge about iron and anaemia and the interaction with their mother/daughter.

Intervention programme: "Follow my Green Food Steps"
In recent years, a number of meta-analytical reviews have been conducted in the area of behaviour change and life-style change and/or food intake behaviours (e.g., Greaves, Reddy, & Sheppard, 2010; Michie, Abraham, Whittington, & McAteer, 2009; Olander et al., 2013; Prestwich et al.; Webb et al., 2010). Although all these reviews comment on the heterogeneity of the models used and the frequent lack of detailed information which behaviour change techniques have been used and how they were operationalised, some general conclusions can be drawn. First, although some reviews indicate that theory-based interventions are more effective than interventions that are less theory-driven (Webb et al., 2010),others indicate that this relationship is weak (Greaves et al., 2010; Prestwich et al., 2014). Our programme employs the framework that was also used for the Lifebuoy hygiene programme which has been shown to be effective (Nicholson et al., 2014)and uses behaviour change techniques that have been shown to be effective. These are the mobilisation of social support (Greaves et al., 2010; Olander et al., 2013), self-regulation techniques (i.e., creating the intention, setting a goal, monitoring behaviour and reviewing of the behavioural goals;Greaves et al., 2010; Michie et al., 2009; Webb et al., 2010) and behavioural prompts/cues. The latter technique was included in the behaviour change programme, as research on habits indicates that providing cues/prompts at the location where the behaviour should be performed increases the likelihood that the behaviour is performed (Danner, Aarts, & de Vries, 2008; Verplanken, 2006; Verplanken & Wood, 2006) and (Olander et al., 2013) found it to have the biggest effect in the area of physical activity.

Based on internal reports on the kinds of ingredients used for dishes in Nigeria, how they are cooked and how much consumers know about anemia and the role of iron in the diet, it became clear that the knowledge about iron in Nigeria was quite low; nutritious food is mainly seen as filling food and only 27% of respondents ranked "added iron" in the top 3 of nutritional benefits sought after by consumers when doing shopping (Ipsos, 2014). Therefore, two additional components were included in the programme, i.e., providing information the role of iron as part of a healthy diet and the consequences of a lack of iron, as well as providing information about how the amount of iron in the diet can be increased through the use of iron-fortified Knorr Bouillon cubes and by adding green leafy vegetables (i.e., a skills training, Webb et al., 2010).

The programme that was created using these building blocks is the "Follow in my Green Food Steps" programme. The programme is built around the insight that both mothers and daughters are hoping

for a better future and that getting more iron in her daughters diet (by adding iron enriched Knorr Cubes and green vegetable to stews) helps her get the most out of life. It leverages the popular game "Do as I do" and uses a song and dance that helps engrain the behaviour change in people's lives. The key message the behaviour change campaign tries to bring across is "Follow my Green Food Steps so that your daughter can take growing changes in her stride as she nourishes her body with more iron the smart and flavoursome way". The idea of the programme is to ensure that there is at least one interaction per week. An overview of the programme is provided below.

The well-known Nigerian actress Omotola and her daughter Meriah will play a crucial part as a role-model for the new behaviours. In addition to showing the desired behaviour in a video in which she shows the Knorr way of cooking ("Toss, stir and crumble"), she will visit a number of locations where the programme is running. Furthermore, a selection of best families will get a chance to meet Omotola and her daughter Meriah. She will also be a guest on a number of radio episodes.

The programme is supported by various media activities that those enrolled in the programme can engage in:

- A radio programme that will be aired during the programme
- An SMS-based subscription service, that will send messages and reminders to those that have subscribed to it (see appendix 9 for an outline). The objective of this is to make a link with the radio drama and reply to questions regarding the programme.
- Interactive Voice Recorded (IVR). The objective of the IVR is to create an individualised interaction to repeat the key messages and reinforce the behaviour. Based on the response from the consumer, IVR will follow an individualised flow of communication (see appendix 9 for an outline).

The programme uses a home-based community approach focusing on the mothers and a school-based programme focusing on the daughters (see Fig. 1 for a description of the flow and Table 1 for a description of how the various media activities are meshed with the community and school-based programme). It will run for 12 weeks.

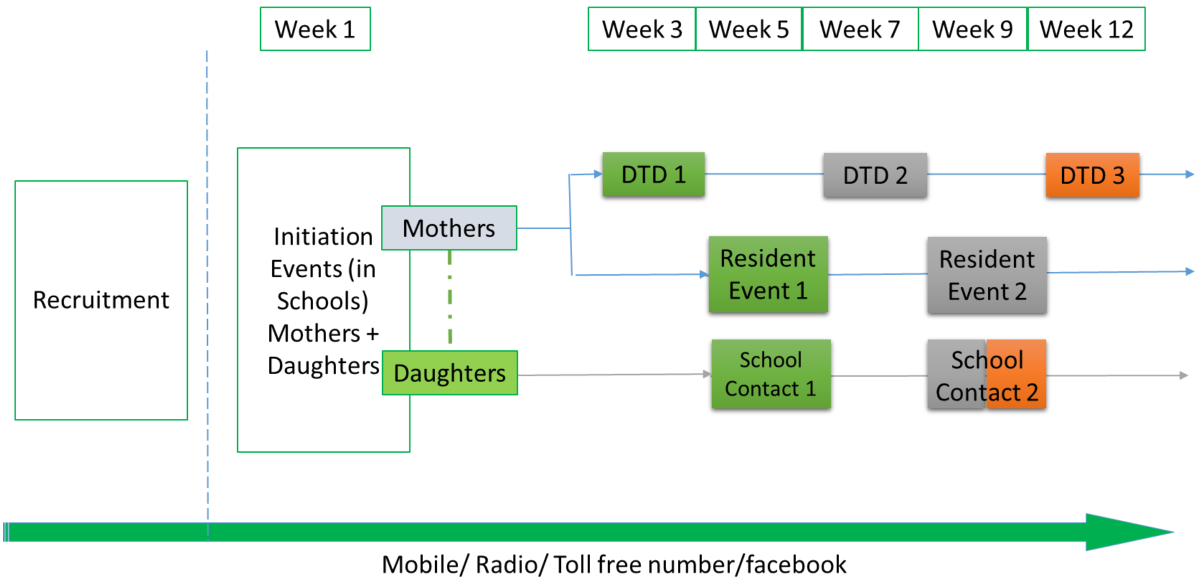

Figure 1. Schematic overview of the behaviour change programme

Overlaying mobile and the program elements													
**Activity**	**Objective**	**Week 1**	**Week 2**	**Week 3**	**Week 4**	**Week 5**	**Week 6**	**Week 7**	**Week 8**	**Week 9**	**Week 10**	**Week 11**	**Week 12**
Initiation event	Flash bulb moment												
DTD 1	Individual interaction-shopping			Shopping									
Resident event 1	Social norm-shopping					Shopping							
School contact 1	Reminder tools					Shopping							
DTD 2	Individual interaction-cooking							Cooking					
Resident event 2	Social norm-cooking									Cooking			
School contact 2										Cooking/Appreciation			
DTD 3	Individual interaction/appreciation												Appreciation
SMS	Link to radio drama/proof of shopping –cooking -appreciation		Welcome to the programme. Link to episode 1		Radio drama 2/Greens shopping proof		Radio drama 3/Interact with Omotola life		Radio drama 4		Radio drama 5		Radio drama 6
IVR	Personal interaction/repeat and reinforce behaviour/feed back	Enrol/profile	Feedback on initiation event										Announce the winners
Radio drama		Episode 1-Flash bulb moment	Consumer engage ment-shopping	Episode 2-shopping	Consumer engage ment-shopping	Episode 3-Mom-daughter bonding	Consumer engage ment-cooking	Episode 4-cooking	Consumer engagement-cooking	Episode 5-Appreciation and appreciation	Consumer engage ment-appreciation and acceptance	Episode 6-Finale - Every one is doing it	Consumer engage ment-grand finale winners
Facebook	Recognition/reward/announcement												
Omotola visits	1-2 times during the programme and 1 grand finale						Be present on the radio show				Visit few of the families		

Table 1: Overlaying mobile and the program elements

The programme starts off with an initiation event at schools for both mothers and daughters. The take-out of the event should be that Mothers and daughters understand the importance of iron deficiency and how with just little effort they can get their daily dose of iron through adding greens and Knorr in their stew. The event should also encourage bonding between the mother and the daughter. During the initiation event, a key teacher and class monitor will also be assigned at each school.
The initiation event consists of four elements:

1) A pre-initiation, during which any girls not engaged during the initial recruitment activities for the behaviour change programme will be engaged to take part in the behaviour change programme. These girls will not be eligible to participate in the impact evaluation study.
2) An introduction session, during which there will be a few short talks about the key Knorr Food Steps messages. Attendees will be presented with the key steps that can help increase iron intake: *Toss* green vegetables into your stews, *Stir* it all together, *Crumble* in iron-enriched Knorr.
3) A demo session consisting of the following elements:
    a. Re-enactment of the radio-drama
    b. Interactive cooking & video session (cooking done by a well-known local chef in the community
    c. A Song & Dance featuring Yemi Alade. This song collates the necessary information on iron deficiency, symptoms associated with iron deficiency, benefits of iron and the two key behaviours in the behaviour change programme.
4) Commitment;
    a. Signing of the commitment card
    b. Sharing of the "stew" calender
    c. A starter kit consisting of Knorr Fortified bouillon cubes and a bunch of pumpkin leaves
    d. A promise letter – to be recited by the mothers and daughters

In order to increase the chance that the target behaviours will be performed, a number of follow-up activities are an integral part of the "Follow in my Green Food Steps" programme. The follow-up activities are centred around the areas where the shopping is done (i.e. with the shops and retailers), in and around the home (targeted at the mothers), and around the schools (targeted at the daughters). The specific activities are described in more detail in the following sections.

Shopping

In and around the areas where people tend to do their shopping, reminders will be placed, such as posters, market entrance signage and retailer outlet signage (meat shops, wet markets).

The mothers

Following the initiation events, the mothers will be contacted at home in 3 door-to-door (DTD) events and two resident events.

DTD1 "Shopping":

The objective of the first door-to-door event is to follow up on the initiation event, initiate new recruits, reinforce "Toss, Stir & Crumble" message as well as the benefits of Knorr Iron Fortified

Cubes and the addition of green leafy vegetables. There will be a special focus on Veggie Shopping experience. The following activities are part of the first door-to-door event:

- Reintroduce the programme
- Repeat key message: Knorr + Green = Iron enriched life for family
- Hand over a shopping list (see appendix x)
- Address shopping list/ Best time to shop for Greens
- Address any issues/ Challenges in following through programme
- Speak on why Knorr must be added to meals
- Assess Calendar & encourage filling
- Inform when the resident event will happen.

DTD2 "Cooking":

The objective of the second door-to-door visit is to follow up on the habit of shopping for green leafy vegetablesand Knorr Fortified Bouillon cubes, reinforce the Knorr way message ("Toss Stir & Crumble" and the benefits of Knorr Iron Fortified Bouillon cubes and the addition of green leafy vegetables. Furthermore, mothers and daughters will be encouraged to spend cooking time together. The following activities are part of the second door-to-door event:

- They will be asked if they have listened to the radio drama and be reminded of the time and dial in details.
- Address queries if they have any
- Check and encourage calendar update/use.
- Hand out reminder stickers that can be put on the calender when they put greens + Knorr in a stew
- They will be asked to write their best Omotala Stew recipe, whichwill be collected the next time/at the resident event, with Collect best recipe by mothers.
- Inform mothers of the resident event (where and when)

DTD 3 "Dish appreciation & acceptance":

The objective of this third door-to-door activity is to help the family appreciate and accept the taste and look of the food. The following activities are part of the third door-to-door event:

- They will be informed that this is the last time they will be visited by the team, and that they will hopefully continue to use the Knorr Green Food Steps.
- They will talk about how the dish has been accepted and address any queries about dish acceptance and appreciation
- The Omotola Stew Recipes will be collected.

- The calender will be collected and that based on this and their participation, they might get to meet Omotola and Meriah

The resident events aims to create a social norm around “Toss, Stir & Crumble”. The first event will focus on shopping, the second on cooking and dish appreciation.

Resident event 1 consists of the following activities:

- Knorr Song & Dance
- Quiz on the radio drama of same week
- Mini Cooking Demo (best recipes from DTD)
- Replay Radio Drama (if required)
- Interactive discussion around shopping

The resident event 2 consists of the following activities:

- Knorr Song & Dance
- Recognize the best recipes.
- Quiz on the radio drama of same week
- Mini Cooking Demo with mom with the best recipe before all.
- Radio Drama of the week replayed
- Interactive discussion around cooking/appreciation

In parallel to the door-to-door contacts and the resident events organised for the mothers, the daughters will participate in two school contacts.

School contact 1:
The objective of the first school contact is to reinforce “Toss, Stir & Crumble” message as well as the benefits of Knorr Iron Fortified Cubes and the addition of green leafy vegetables. The following activities are part of the first school contact:

- Introduction, including scientific knowledge on why it’s important to have greens and Knorr
- The children are provided with extra green stickers
- The children’s version of the Yemi Alade song will be collected (pictures, dance)
- If the children have prepared a performance, they should give it and the RA should record it (1 per class max)
- Children will be asked to come up with good ideas to make their mothers remember to buy greens. Best ideas will be rewarded.

- Inform them that at the next school event, there will be a cooking competition, and contestants will be chosen with a lucky draw. They will need to prepare an Omotola Stew recipe created by their mothers. They will be handed over the Omotola Stew basic recipe that they should discuss with their mothers and adapt.
- Interactive activity with pupils via drama depicting connection between mother and daughter.
- They will be informed of the next visit
- The session will end with the Knorr Song & Dance

The following assets will be used: cooking set, branding, green reminder, stickers

School contact 2:

The objective of the second school contact is to further reinforce the key messages and monitor the practice of new Food Steps and consist of the following activities:

- Get feedback on cooking calendar use
- Give feedback on experience with spending more time with mother – Connection to mother
- Knorr Song & Dance – Pupils to perform their own version.
- Collect photographs
- Recognize and reward best photos
- The children will be provided with extra green stickers

The following assets will be used: cooking set, branding.

Following the intervention programme, post-intervention measures will be taken for the cooking behaviours, the behavioural determinants, knowledge and the mother-daughter interaction. Furthermore, the intervention group will be asked questions to evaluate how much the participants enjoyed participating in the programme itself.

A schematic outline of the study is provided in Fig. 2.

Recruitment

Interviews<br>• Informed consent<br>• Demographics [Mother]<br>• Food intake (behaviour) [Mother & Daughter together]<br>• Shopping/cooking questions [Mother]<br>• Knowledge [Mother and Daughter seperately]<br>• Determinants of behaviour [Mother and Daughter seperately]<br>• Mother/Daughter interaction [Mother and Daughter seperately]	Interviews<br>• Informed consent<br>• Demographics [Mother]<br>• Food intake (behaviour) [Mother & Daughter together]<br>• Shopping/cooking questions [Mother]<br>• Knowledge [Mother and Daughter seperately]<br>• Determinants of behaviour [Mother and Daughter seperately]<br>• Mother/Daughter interaction [Mother and Daughter seperately]
Follow in my Green Foodsteps Programme	No intervention
Interviews<br>• Food intake (behaviour) [Mother & Daughter together]<br>• Knowledge [Mother and Daughter seperately]<br>• Determinants of behaviour [Mother and Daughter seperately]<br>• Mother/Daughter interaction [Mother and Daughter seperately]<br>• Evaluation questionnaire	Interviews<br>• Food intake (behaviour) [Mother & Daughter together]<br>• Knowledge [Mother and Daughter seperately]<br>• Determinants of behaviour [Mother and Daughter seperately]<br>• Mother/Daughter interaction [Mother and Daughter seperately]

Figure 2. Schematic outline of the study flow

Ethical consideration
Risks and Discomforts:

There is a slight risk that participants may feel uncomfortable talking about some of the topics. However, we do not wish this to happen, and participants may refuse to answer any question or not take part in a portion of the discussion if participants feel the question(s) makes participants uncomfortable.

Benefits:

There will be no direct benefit to participants but the information obtained from this study will help to provide suggestions that will enable the researchers develop an appropriate intervention program to enable mothers increase the intake of green leafy vegetables and iron-fortified Knorr Bouillon.

Liability and Insurance statement

It is Unilever's policy to provide compensation to subjects participating in studies in the extremely unlikely event that they suffer any injury or deterioration in health as a result of participating in the study.

Timing

After ethics approval has been granted it is intended that the study will commence in August 2015 and data collection will be finalised by the end of February 2016.

Incentives:

We will provide participants with a present of napkin or Unilever product as a token of our appreciation of their time and effort for participating in this interview.

Confidentiality:
We have taken the following steps to ensure that participants are safe and that the information participants provide is confidential.

1. The interview will take place in a private place as convenient for the respondents, where no one else hears what participants discuss with the interviewer.
2. The information that we collect from this research project will be kept confidential.
3. Information collected from participants will be stored in a file that will not have their name on it, but a number assigned to it instead.
4. The questionnaire containing the interview will be stored for the duration of the study (2 years) after which it would be destroyed.

5. There will be no name associated with the number assigned to each file and such will be kept under lock and key and will not be disclosed to anyone except staff working on this study.
6. Participants may talk to the leader of the research team in case they have any concern or questions.

Alternative to Participation/Voluntariness:

Participants do not have to take part in this research if they do not wish to do so, and refusing to participate will not affect you future at the community/ school. Even if they do not wish to answer these questions they may still benefit from the study. Participant may stop participating in the interview at any time that they wish, and there will be no negative consequences for them in any way.

Statistical Analysis

Two dish-specific food intake behaviours are targeted and that the dependent measure will be specifically developed for this study. Furthermore, the intervention has been developed from scratch.

Assuming that the number of adding green leaves to a dish is a binomial distribution, sample sizes can be calculated for comparing two binomial distributions based on proportions per group. These proportions indicate the frequency of adding green leaves to a dish per week. Some assumptions were made for these proportions. It is assumed that subjects make 7 dishes per week where green leaves can be added. It is assumed that some of the subjects of the control group do add green leaves to their dishes (a proportion is needed for the calculation). In the sample size consideration two proportions for the control group are assumed, 0.05 and 0.07. These can be seen as 0.35x per 7 days or 0.5x per 7 days of adding green leaves to dished (overall mean of the group). For the test group is two proportions are tested, 1x per 7 days (0.14) or 2x per seven days (0.28). The alpha used for testing is 0.05 and the power is 0.8). *Table 2*2 shows the result of the sample size consideration.

Table 2: Sample size consideration with Number of Subjects per group

Proportion1	Proportion2	Alpha	Nominal Power	NPerGroup
0.05	0.14	0.05	0.8	166
	0.28	0.05	0.8	40
0.07	0.14	0.05	0.8	300
	0.28	0.05	0.8	51

So for testing if the test group has a behaviour change (increase) of 2x per week (0.28) 40 subjects per group are required when assumed that the control group has a usage of 0.35x per week (0.05).

Decreasing the frequency for the test group to 1x per week (0.14) results in a major increase in number of subjects per group (166 per group). Increasing the assumed usage of the control group to 0.5x per week also results in an increase in number of subjects needed per group.

Based on these sample size considerations, as well as the fact that this is the first time that the programme is being run and it would be desirable to show that the programme increases the target behaviour by 1x per week, and the fact that 200 respondent are required for SEM analyses per subgroup, 100 households or 200 respondents (i.e., 100 mothers and 100 daughters) will be recruited per community, leading to a total of 800 respondents.

Differences between the intervention and control group
The primary analysis to determine the effect of the intervention will be based on calculating the differences in intake of Knorr bouillon cubes and green leafy vegetables between pre- and post-intervention measurements. A multilevel model will be used to estimate the average intake and the variation for each group of households. A t-test can then be used to compare the two groups using these estimates. Significance levels will be set at $\alpha = 0.05$. Furthermore, associations between behavioural determinants and the size of the reduction will be investigated, to identify determinants of change, using SEM.

Secondary explorative analyses might include investigating whether mothers and daughters report discussing iron, anaemia and cooking skills more in the intervention group versus the control group.

For the analyses of the impact of behaviour determinants on intake, Structural Equation Modelling (SEM) are a preferred methodology. This requires at least approximately 200 households per group, depending on the number of variables included in the measurement model.

Descriptive analyses
Descriptiveanalyses on the baseline data include:
1) Frequency intake of Knorr bouillon cubes, stews, soups and the key ingredients in the recipes;
2) Average scores on factors measuring the behavioural determinants;
3) Average scores on the measures of the interaction between mother and daughter;
4) Analyse whether the independent variables mentioned in bullet 1 and 2 are dependent on demographic and psychographic variables such as community location, household size, age, gender and family income.

<u>Reporting</u>
The results of these analyses will be reported in an internal scientific Unilever report and Power-point presentations that will be presented internally and externally as appropriate. Furthermore, it is the intention to publish the data in a peer-reviewed academic journal.

MANAGEMENT PLAN

RESEARCH TASKS, RESPONSIBILITIES AND RELATIONSHIPS

Our team is composed of three key players to include Unilever, EXP and Faculty of Public Health, University of Ibadan. The descriptions of each group's roles and responsibilities are described below

Unilever will be responsible for

- Funding
- Monitoring
- Tracking progress

The EXP will be responsible for

- Selection of participants in the intervention communities
- Behaviour Changeintervention
    - Building awareness of iron deficiency and Knorr way
    - Trial and commitment
    - Repetition and reward

University of Ibadan will be responsible for

- Selection of communities in the control group
- Training and fieldwork
- Execution of the entire baseline and post intervention activities
- Management of field operations
- Administration and collection of all surveys
- Supervision of data collection
- Analysis of data

EXPERIENCE IN RESEARCH AND TECHNICAL AREAS

In 1948, the University College, Ibadan was established under the University of London. Shortly after independence, in December 1962, the Nigerian Federal Parliament passed a bill for an Act to establish the University of Ibadan. From the 27th of December 1962, the University became an autonomous institution. The University is governed by a Council with embers appointed by the Visitor of the University, i.e. the President of the country. In the 1977/78 session the then Federal Military Government designated our Faculty of Medicine as a Centre of Excellence in Medicine amongst Nigerian Universities.

The College plays its traditional roles of teaching, research and community service and consultancy through faculties, departments, institutes and other specialized centres that are linked with the College Consultancy Services Unit.

The Faculty of Public Health has the Departments of Health Promotion and Education, HPE (formerly the African Regional Health Education Centre, ARHEC) as foundation Department, Epidemiology and Medical Statistics, Environmental Health Sciences, Human Nutrition and Dietetics, Institute of Child Health, Health Policy and Management and Primary Care and Preventive Medicine with a pool of Public health specialists. The Staff of the Faculty of Public Health, University of Ibadan have been major players in the NPHCDA projects, one of which is capacity building. For example, in the past five years the Faculty has carried out projects for NPHCDA including health systems reform through Stakeholders including the World Bank, Pathfinder, ARFH, WHO, UNICEF, USAID, CEDPA, IDRC and PERFRON.The team drawn from the Faculty of Public Health is therefore well placed and specialised to carry out the consultancy satisfactorily and successfully.

Management of Research Funds: In regards to managerial capacity for research funds the team members have received and successfully managed numerous grants for research, evaluation and consultation from the World Health Organization (TDR), the Rockefeller Foundation, the John D. and Catherine T. MacArthur Foundation, USAID (e.g. BASICS, WASH), UNICEF, Africare, US Peace Corps, Advocates for Youth,the World Bank, AMREF, PATH, UAPS, CEDPA, NPHCDA, and IDRC among others.

ADMINISTRATIVE AND FINANCIAL MANAGEMENT

University of Ibadan is an academic University with vast experience in managing sponsored awards and sub-recipient relationships. The University has policies and procedures in place for receipt of invoices to monitor recipient use of funds and performance of work, which shall be maintained and followed throughout the duration of this activity.

COMPLIANCE AND ETHICAL STANDARDS

The University of Ibadan has a well-established Institutional Review Board that will be responsible for review and approval of protocol and procedures described in this proposal.

Communication and Coordination with UNILEVER

The consultant will establish communication schedule with Unilever for all activities related to theconsultancy. The form of communication will be through emails and conference calls. This communication schedule is requested for information clarification and reply purposes.

PERSONNEL AND STAFFING

Consultants’ Qualifications

The Lead consultant Dr Oyedunni Arulogun, holds a B.Ed and M.Ed degrees in Special Education, a Master of Public Health and PhD in Health Promotion and Education. She had been involved in work that improved the quality of life of persons with disabilities. Such included Effect Of Drama As A Communication Tool In Promoting Safe Reproductive Health Practices Among In-School Deaf Junior Secondary One Students In Ibadan, Nigeria funded by John D. & Catherine T. MacArthur Foundation (2002 – 2004), Child Fosterage Promises and Trafficking in Children for Domestic Work in Nigeria: Implications for Reproductive Health funded by NDP/UNFPA/WHO/WORLD BANK Special Programme of Research, Development and Research Training in Human Reproduction [HRP] (2007 – 2008), Exploring the sexual and reproductive health needs of deaf Girls in Ibadan, Oyo state, Nigeria WHO Grant on Deaf girls UNDP/UNFPA/WHO/WORLD BANK Special Programme Of Research, Development And Research Training In Human Reproduction (2010 – 2012), Games and Sports as Indicators of Physical and Mental Development of the Visual Impaired Individuals and Comparison of blood pressure in deaf secondary School Children and their Hearing Counterparts: Association between Noise and Blood Pressure.Similar consultancies done in collaboration with other experts included communication consultancy for Center for Communication Program Nigeria (CCPN); 09-8754094 at several occasions and Consultancy for Project Proposal Development Access to Maternal and New Born Health Services For Persons With Disabilities for Handicap International (HI) Mano River (Liberia & Sierra Leone), November 2013.

The co-consultant has a Master of Public Health degree with expertise in quantitative research methods. He has also been involved in similar work as the lead consultant.

Dr. René Lion holds a Masters in Social Psychology from Radboud University Nijmegen and a PHD in Health Promotion & Education from Maastricht University. He has worked as a consumer scientist with Unilever R&D Vlaardingen for the past 15 years. His main areas of interest involve risk perception and communication and behaviour change, especially in the area of food intake (e.g., sodium reduction).

Draft Instrument

Appendix 1. Demographics [only at baseline]

S1. Please indicate your age, in years.___________ Years

[if age 12- 18, continue with behavioural questionnaire; if age >18 and mother continue with S3]

S2. We are looking for people working in specific industries. Which most closely describes the industry in which you work?

1. Farming
2. Trading
3. Artisan
4. Civil Servant (Teacher etc)
5. Housewife
6. None of the above

S3. Can you please specify your level of involvement in each of the household tasks below?

	Household tasks	High	Moderate	Low
1	Cooking			
2	Daily grocery shopping			

S4. Can you please specify if responsible for the following activites

1. I am fully responsible for this in my household
2. I am responsible for this together with someone else
3. I am not responsible for this task

	Household tasks	Responsible	Responsible with someone else	Not Responsible
1	Cooking			
2	Daily grocery shopping			

Screen-out if not (co-)responsible for cooking AND grocery shopping (if S4_1=3 and S4_2=3).

S5. Are you a vegetarian?

1. Yes
2. No

S6. Are you <u>currently</u> on a particular diet? (for weight loss, diabetes, hypertension etc.)

1. Yes
2. No

S7. How many people live in your household including yourself? ___________________________

S8. Do you have any children living in your household?

1. Yes
2. No– TERMINATE (should not be part of the sample)

<skip if S7=1 orS8=2>

S9. How many children, in each of the following age groups, live in your household?

1. 16+ years _____children
2. 12-15 years old _____children
3. 9-11 years old _____children
4. 6-8 years old _____children
5. 3-5 years old _____children
6. 0-2 years old _____children

S10. What is your marital status?

1. Married / living together with partner
2. Widowed
3. Divorced
4. Other, please specify:_______________________________

S11. What is your level of education?

1. None

2. Primary
3. Secondary
4. Tertiary

S12. What is your professional situation?

1. Employment, full time
2. Employment, part time
3. Student
4. Homekeeper
5. Currently not employed
6. Others, pleasespecify:

S13. Which of the following categories best describes the total net monthly income of your household?

1. <₦10,000
2. Between ₦10,000 – ₦20,000
3. Between ₦20,000 – ₦50,000
4. Between ₦50,000 – ₦100,000
5. Above ₦100,000

<u>Section 2. General questions related to shopping and cooking</u> [only during baseline]

C1. For how many people do you usually cook?______________________________

C2. Which of the following utilities do you have in your home?

**Utilities**	**Yes**	**No**
Electricity		
Water (piped directly into your home		
Air conditioning (built in NOT portable)		
Electronic home security system (e.g. burglar alarm)		
Seated / non-seated flushing toilet (NOT manually flushed using bucket)		
No seated / non-seated flushing toilet		

C3. Which of these items do you have in working order in your home?

**Items have in working order**	**Yes**	**No**
Kitchen sink		
Dining table		
Primus (paraffin stove) / cylinder based gas stove		
Refrigerator (Stand alone – this may have ice box but NOT separate freezer section with its own door)		
Fridge freezer (i.e. the freezer section has its own door, separate from the refrigerator door)		
Separate deep freezer		
Cooker (i.e. oven & hob appliance)		
Electric kettle		
Microwave oven		
Dish washing machine		
Electric deep fat fryer		
Electric mixer		
Water filtering device		
Hi-Fi/stereo system (cassette, CD or vinyl)		
Video player/recorder (VCR)		
DVD/Blu-ray player/recorder		
Docking station (for MP3 / iPod)		
PC (laptop/computer)		
Mobile phone (not smartphone)		
Smartphone		

C4. How easy is it for you to get the following ingredients?

1. Very difficult
2. Difficult
3. Not easy, not difficult
4. Easy
5. Very easy

**Ingredients**	
Knorr Bouillon cubes	
Other bouillon cubes	
Green leafy vegetables (e.g., spinach, ugwu)	
Fresh green vegetables (e.g., okra)	
Tomatoes	
Red bell peppers (Tatache)	
Fresh fruits	
Onion	
Meat	
Fish	
Fresh herbs	
Spices (ginger and galic)	
Fresh drinking water	

C5. How affordable are the following ingredients for you?

1. Very unaffordable
2. Unaffordable
3. Not affordable, not unaffordable
4. Affordable
5. Very affordable

**How affordable are the following items**	
Knorr Fortified Bouillon cubes	
Other fortified bouillon cubes	
Green leafy vegetables (e.g., spinach, ugwu)	
Fresh green vegetables (e.g., okra)	
Tomatoes	
Fresh fruits	
Red bell peppers (Tatache)	
Onion	
Meat	
Fish	
Fresh herbs	
Spices (Ginger and galic)	
Drinking water	

Section 3. Behavioural questionnaire

A1. During the past month, on average how often have you cooked soup?

	Egusi/melon soup	Ewedu soup	Vegetable soup	other
Never during the past month				
Once a month				
Twice a month				
Three times a month				
Once a week				
Twice a week				
Three times per week				
Four times per week				
More than four times a week				

A2. When cooking egusi soup

- Which of the following ingredients did you add?
- How much of each ingredient did you add

A2a. EGUSI/MELON SOUP			
**Name of ingredient**	**Tick box if added**	**Indicate amount**	**Serving size**
Onions			
Tatashe (red bell pepper)			
Tomatoes			Medium-sized tomatoes
Ground melon seeds (Egusi)			Milk tins
Knorr cubes			cubes
Other Cubes			cubes
Green leafy vegetables (ugwu, spinach, amaranth leaves, cassava leaves, cocoyam leaves)			bunches
Green vegetables (bell pepper, okra)			bowls

A2b. EWEDU SOUP			
**Name of ingredient**	**Tick box if added**	**Indicate amount**	**Serving size**
Onions			
Tatashe (red bell pepper)			
Tomatoes			Medium-sized tomatoes
Ground melon seeds (Egusi)			Milk tins
Knorr cubes			cubes
Other Cubes			cubes

Green leafy vegetables (ugwu, spinach, amaranth leaves, cassava leaves, cocoyam leaves)			bunches
Green vegetables (bell pepper, okra)			bowls

**A2c. VEGETABLE SOUP**			
**Name of ingredient**	**Tick box if added**	**Indicate amount**	**Serving size**
Onions			
Tatashe (red bell pepper)			
Tomatoes			Medium-sized tomatoes
Ground melon seeds (Egusi)			Milk tins
Knorr cubes			cubes
Other Cubes			cubes
Green leafy vegetables (ugwu, spinach, amaranth leaves, cassava leaves, cocoyam leaves)			bunches
Green vegetables (bell pepper, okra)			bowls
Onions			

**A2d. OTHER SOUP [PLEASE INDICATE]**			
Name of ingredient	Tick box if added	Indicate amount	Serving size
Onions			
Tatashe (red bell pepper)			
Tomatoes			Medium-sized tomatoes
Ground melon seeds (Egusi)			Milk tins
Knorr cubes			cubes
Other Cubes			cubes
Green leafy vegetables (ugwu, spinach, amaranth leaves, cassava leaves, cocoyam leaves)			bunches
Green vegetables (bell pepper, okra)			bowls

B1. During the past month, on average how often have you cooked any of the following stews?

	**Fish stew**	**Beef stew**	**Goat stew**	**Fried stew**	**other**
Never during the past month					
Once a month					

Twice a month					
Three times a month					
Once a week					
Twice a week					
Three times per week					
Four times per week					
More than four times a week					

B2. When cooking stews

- Which of the following ingredients did you add?
- How much of each ingredient did you add?

**B2a. BEEF STEW**			
**Name of ingredient**	**Tick box if added**	**Indicate amount**	**Serving size**
Meat			
Onions			
Tatashe (red bell pepper)			
Tomatoes			Medium-sized tomatoes
Ground melon seeds (Egusi)			Milk tins
Knorr cubes			cubes
Other Cubes			cubes
Green leafy vegetables (ugwu, spinach, amaranth leaves, cassava leaves, cocoyam leaves)			bunches
Green vegetables (bell pepper, okra)			bowls

**B2b. FISH STEW**			
**Name of ingredient**	**Tick box if added**	**Indicate amount**	**Serving size**
Meat			
Onions			
Tatashe (red bell pepper)			
Tomatoes			Medium-sized tomatoes
Ground melon seeds (Egusi)			Milk tins
Knorr cubes			cubes
Other Cubes			cubes
Green leafy vegetables (ugwu, spinach, amaranth leaves, cassava leaves, cocoyam leaves)			bunches
Green vegetables (bell pepper, okra)			bowls

B2c. GOAT STEW			
**Name of ingredient**	**Tick box if added**	**Indicate amount**	**Serving size**
Beef			
Onions			
Tatashe (red bell pepper)			
Tomatoes			Medium-sized tomatoes
Ground melon seeds (Egusi)			Bowls
Knorr cubes			cubes
Other Cubes			cubes
Green leafy vegetables (ugwu, spinach, amaranth leaves, cassava leaves, cocoyam leaves)			bunches
Green vegetables (bell pepper, okra)			bowls

B2d. OTHER STEW			
**Name of ingredient**	**Tick box if added**	**Indicate amount**	**Serving size**
Beef			
Onions			
Tatashe (red bell pepper)			
Tomatoes			Medium-sized tomatoes
Ground melon seeds (Egusi)			Milk tins
Knorr cubes			cubes
Other Cubes			cubes
Green leafy vegetables (ugwu, spinach, amaranth leaves, cassava leaves, cocoyam leaves)			bunches
Green vegetables (bell pepper, okra)			bowls

C. How often have you prepared any of the following dishes during the past month?

	Boiled rice	Jollof Rice	Side dish of green leafy vegetables	Fried Rice	Other	Other
Never during the past month						
Once a month						
Twice a month						
Three times a month						
Once a week						
Twice a week						
Three times per week						
Four times per week						
More than four times a week						

Section 4. Knowledge/awareness questions on Iron deficiency

K1. **ASK ALL.** How often do you experience these symptoms?

	**Hardly ever or never**	**Less than once a month**	**Once a month**	**Once every 3 weeks**	**Once every 2 weeks**	**Once a week**	**3-5 times a week**	**Once a day**	**More than once a day**
Poor concentration									
Tiredness, even though you have had enough sleep									
Increased irritability, when nothing has really gone wrong									
Dizziness									
Paler complexion									

K2. Have you ever heard of a condition called "anaemia"?

Yes = 1 – continue to K3

No = 2-If No go to K5

K3: Which of these conditions/situations increase the risk of anaemia?

Show the screen to the respondents. Read the question and select the options (Multiple coding possible).

Family history of anaemia	01
Being underweight	02
Being overweight/obese	03
Having a large waist/ a tummy	04
High fat diet	05
Stress/ tension	06
Excessive intake of sweets/ sugar	07
Lack of physical activity	08
Low intake of green leafy vegetables such as ugwu, spinach, amaranth leaves, cassava leaves, cocoyam leaves	09
Low intake of red/orange vegetables (e.g., red pepper, tomato)	10
Low intake of green vegetables such as green beans, okra	11
Low intake of purple, white vegetables (e.g., egg plant, cassava)	12
Low intake of fruits	13
Low intake of red meat (beef, goat)	14
Low intake of fish	15
Low intake of eggs	16
Low intake of beans	17
Lack of sleep	18
Smoking	19
Being pregnant	20
Having your period	21
Don't know/ Can't say (Unaided)	22
Others ___________	98

K4: Which of these are effective solutions for "anaemia"?
Read the question and select the options. *Multiple coding possible*.

	**Yes (1)**	**No (2)**
Eating vegetables as part of a meal		
Crushing, grinding and drinking the juice from pumpkin leaves		
Drinking herbal mixes		
Eating unripe plantains		
Consuming liver		
Consuming 'blood tonic' or 'blood tablets'		
Consuming green leafy vegetables, such as ugwu, spinach, amaranth leaves, cassava leaves, cocoyam leaves		
Consuming red/orange vegetables such as red pepper, tomato, pumpkins		
Consuming purple, white vegetables (e.g., egg plants)		
Consuming fruits		
Consuming more red meats (e.g., beef, goat)		
Consuming more eggs		
Consuming more fish		
Consuming more beans		
Getting more sleep		
Being more physically active		
Don't know/ Can't say (Unaided)		
Others ____________		

K5. Please select which of these describes how much you agree or disagree with the following statements?

	**Agree Strongly (5)**	**Agree Slightly (4)**	**Neither/Nor (3)**	**Disagree Slightly (2)**	**Disagree Strongly(1)**
Iron is good for the health					
Eating foods containing iron helps build red blood cells					
Eating foods containing iron helps build white blood cells					
Foods containing iron strengthens the immune system					
Iron has no healthy benefit for the body					
You cannot consume too much iron					
Iron helps your blood carry oxygen through the body					
Eating iron rich foods helps my children develop					
Consuming foods containing iron helps with concentration/focus					
Consuming foods containing iron helps me have more energy during the day					
Consuming foods containing iron can help reduce your dizziness					

K6. Which of these foods do you think are good sources of iron?

	Food that are good sources of iron	**Yes (1)**	**No (2)**
1	Eggs		
2	Fish		
3	Red meat		
4	Liver		
5	Wholemeal bread & flour		
6	Beans		
7	Nuts (Almonds, Cashews, Walnuts, Peanuts, etc)		
8	Green vegetables (Spinach, Ugwu, amaranth, etc)		
9	Cocoa, Chocolate		
10	Rich fruit cake		
11	Grains (Oatmeal, Rice, Millet, etc)		
12	Others specify		

Section 5. Determinants of Behaviour Questionnaire

KNORR BOUILLON CUBES

1. Attitude

For me, using Knorr Bouillon cubes when cooking stews makes the stew

a.	Very unpleasant						Very pleasant
b.	Very bad						Very good
c.	Very unhealthy						Very healthy
d.	Not at all tasty						Very tasty
e.	Not at all delicious						Very delicious
f.	Very disgusting						Not at all disgusting

2. Social Norm (Injunctive/Husband/father)

a. My husband/father thinks I should add Knorr Bouillon cubes to stews

Completely disagree						Completely agree

b. I think my husband/father expects me to use Knorr Bouillon cubes when cooking stews

Completely disagree						Completely agree

c. When I add Knorr Bouillon cubes to my stew, my husband/father would

Strongly disapprove						Strongly approve

3. Social Norm (Injunctive)

a. Most people who are important to me think I should add Knorr Bouillon cubes to stews

Completely disagree						Completely agree

b. I think others expect me to use Knorr Bouillon cubes when cooking stews

Completely disagree						Completely agree

c. When I add Knorr Bouillon cubes to my stew, the people in my life whose opinions I value would

Strongly disapprove						Strongly approve

4. *Social Norm (Deductive)*

a. Most people who are important to me add Knorr Bouillon Cubes to stews

Completely disagree						Completely agree

b. The people in my life whose opinion I value, add Knorr Bouillon cubes to their stews

Completely disagree						Completely agree

c. In your community, how many people would you say add Knorr Bouillon cubes to their stews.

Very few						Almost all

5. *Perceived Behavioural Control*

a. For me, adding Knorr Bouillon to every stews I cook would be

Very difficult						Very easy

b. If I wanted to, I could add Knorr Bouillon cubes to every stew I cook

Definitely false						Definitely true

c. To what extent is it up to you to add Knorr Bouillon Cubes to the stews you cook?

Not at all up to me						Completely up to me

4. Habit

a. *Addin*g Knorr Bouillon cubes to my stews is something I do automatically

Completely disagree						Completely agree

b. Adding Knorr Bouillon cubes to my stews is something I do without having to consciously remember

Completely disagree						Completely agree

c. Adding Knorr Bouillon cubes to my stews is something I do without thinking

Completely disagree						Completely agree

d. Adding Knorr Bouillon cubes to my stews is something I start doing before I realize I'm doing it

Completely disagree						Completely agree

5. Intention

a. I intend to add Knorr Bouillon cubes to my stews in the next months

Extremely unlikely						Extremely likely

b. I plan to add Knorr Bouillon cubes to my stews in the next months

Completely disagree						Completely agree

c. I will add Knorr Bouillon cubes to the stews I make in the next months

Definitely false						Definitely true

Green Leafy Vegetables

6. Attitude

For me, adding green leafy vegetables(e.g., ugwu, spinach, amaranth) to my stews, makes my stews

a. Very unpleasant						Very pleasant
b. Very bad						Very good
c. Very unhealthy						Very healthy
d. Not at all tasty						Very tasty
e. Not at all delicious						Very delicious
f. Very disgusting						Not at all disgusting

7. Social Norm (Injunctive/husband/father)

a. My husband/father thinks I should add green leafy vegetables to my stews

Completely disagree						Completely agree

b. My husband/father expects me to addgreen leafy vegetables when cooking stews

Completely disagree						Completely agree

c. When I add green leafy vegetables to my stew, my husband/father would

Strongly disapprove						Strongly approve

8. Social Norm (Injunctive)

a. Most people who are important to me think I should add green leafy vegetables to my stews

Completely disagree						Completely agree

b. I think others expect me to addgreen leafy vegetables when cooking stews

Completely disagree						Completely agree

c. When I add green leafy vegetables to my stew, the people in my life whose opinions I value would

Strongly disapprove						Strongly approve

9. Social Norm (Deductive)

a. Most people who are important to me add green leafy vegetables to stews

Completely disagree						Completely agree

b. The people in my life whose opinion I value, add green leafy vegetables to their stews

Completely disagree						Completely agree

c. In your community, how many people would you say add green leafy vegetables to their stews.

Very few						Almost all

10. Perceived Behavioural Control

a. For me, adding green leafy vegetables to every stews I cook would be

Very difficult						Very easy

b. If I wanted to, I could add green leafy vegetables to every stew I cook

Definitely false						Definitely true

c. To what extent is it up to you to add green leafy vegetables to the stews you cook?

Not at all up to me						Completely up to me

11. Habit (Gardner et al., 2012)

a. Adding green leafy vegetablesto my stews is something I do automatically

Completely disagree						Completely agree

b. Adding green leafy vegetables to my stews is something I do without having toconsciously remember

Completely disagree						Completely agree

b. Adding green leafy vegetablesto my stews is something I do without thinking

Completely disagree						Completely agree

c. Adding green leafy vegetablesto my stews is something I start doing before I realize I'm doing it

Completely disagree						Completely agree

12. Intention

a. I intend to add green leafy vegetables to my stews in the next months

Extremely unlikely						Extremely likely

b. I plan to add green leafy vegetables to my stews in the next months

Completely disagree						Completely agree

c. I will add green leafy vegetables to the stews I make in the next months

Definitely false						Definitely true

Section 6: Mother – daughter interaction

Please can you indicate how often you discuss the following subjects with your mother/daughter?

	**Hardly ever or never**	**Less than once a month**	**Once a month**	**Once every 3 weeks**	**Once every 2 weeks**	**Once a week**	**3-5 times a week**	**Once a day**	**More than once a day**
Cooking skills									
How to cook healthily									
Feeling dizzy, tired									
How you/your daughter is doing at school									
What she discusses with her friends									
How well you/your daughter can concentrate									
How much energy you/your daughter feel you have									

Section 7: Post Intervention Evaluation Questionnaire

[Only for the intervention group]

You participated with you family in the "Follow in my Green Food Steps" programme. We would like to ask you a number of questions about your thoughts on this programme.

Q1. Have you participated in other programmes, such as malaria, HIV/AIDS, maternal and child healthcare?

1. Yes
2. No

Please indicate which programme you have participated in:

Q2. On a scale from 1 to 10, with 1 indicating you felt the programme was very bad and 10 indicating you thought the programme really could not be better, how would you grade the programme?
1-10

Q2b. What did you like about the programme?____
____
Q20c. What could be improved about the programme?____
____

Q3. Did you start discussing iron deficiency and its consequences with your daughter/mother as a resultof this programme?

1. Yes
2. No

Q4. To what extent did you enjoy discussing iron deficiency with your daughter/mother?

1. Like it a lot
2. Liked it
3. Neutral
4. Disliked it
5. Disliked it a lot

Q5. To what extent did you enjoy discussing the green food steps with your daughter/mother?

1. Like it a lot
2. Liked it
3. Neutral
4. Disliked it

5. Disliked it a lot

Q6. During and after the “Follow my green Food Steps” programme, I have started using Knorr Bouillon cubes in my stews

1. A lot less
2. A bit less
3. The same
4. A bit more
5. A lot more

Q7. During and after the “Follow my green Food Steps” programme, I have started adding leafy green vegetables to my stews

1. Not at all
2. Once every two weeks
3. Once every week
4. Twice a week
5. Three times a week
6. Four times a week

Q8. Below are a few questions about the topic of the programme. Can you indicate to what extent you agree or disagree with these statements?

1. Completely agree
2. Agree a little
3. Disagree
4. Disagree a little
5. Completely disagree

1. The programme has made me think more about iron deficiency
2. This programme has made me more aware of how I can increase the iron in my meals
3. This programme has made me interact with my daughter/mother in a morepositive way

Q9. How likely is it that you will your recipes on to your daughter with the intention of her using that recipe with her family in the future?’

1. Not at all likely
2. Not so likely
3. Neutral

4. Likely
5. Very likely

Q10. How likely is it that you will use your mother's recipes when cooking for your own family in the future?

1. Not at all likely
2. Not so likely
3. Neutral
4. Likely
5. Very likely

Q11. How important is it for you to pass down your cooking skills/style/recipes to your daughter so she uses this in the future?'

1. Not at all likely
2. Not so likely
3. Neutral
4. Likely
5. Very likely

Q12. How much do you value the cooking skills that your mother teaches you?

1. Not Valued
2. Slightly Valued
3. Neutral
4. Moderately value
5. Highly value

Q13. Will you include leafy green vegetables in stews that you serve your family in the future?

1. Not at all likely
2. Not so likely
3. Neutral
4. Likely
5. Very likely

Q14. Please can you indicate how much you (dis)liked the elements in the Green Food Steps programme.

1. Like it a lot
2. Liked it
3. Neutral
4. Disliked it
5. Disliked it a lot

	Elements in the Green Food Steps programme	
a.	Song and Dance	
b.	Celebrity and daughter	
c.	Take-home bag	
d.	Reminder song	
e.	Commitment card	
f.	Indicating that you performed would perform the behaviour on the commitment card	
g.	Making a video at home	
h.	Caller-back ring tune	
i.	Gift provided for the commitment card and the video	

Q15. How do you feel about the role that Knorr plays in the Follow My Green Food Steps programme?

1. Like it a lot
2. Liked it
3. Neutral
4. Disliked it
5. Disliked it a lot

Q16. Do you have any suggestions on how we might improve the programme?________________________
____

Q17. Do you have any other remarks about the programme?____
____

THANK YOU FOR THE TIME SPENT WITH ME

O. Oladepo
B.Sc (Ib); MPH (Ib.); PhD (Ib.); FRSPH (UK)
Professor
oladepod@yahoo.com
ooladepo@comui.edu.ng

A. J. Ajuwon
B.Sc (Lagos); MPH (Ib.); PhD (Ib.)
Professor
ajajuwon@yahoo.com

J. D. Adeniyi
B.A. (Ife) MPH (Chapel Hill); ARSH (Lond.); Dr. PH (John Hopkins)
Professor/ Adjunct Lecturer
jdadeniyi@yahoo.com

Oyedunin S. Arulogun
B.Ed. (Ib); M.Ed (Ib); MPH (Ib.); PhD (Ib.), FRSPH (UK); CCST (Nig)
Reader
oyedunniarulogun@gmail.com

F. O. Oshiname
B.Sc (Benin); MPH (Ib.); M.A (Cleveland); PhD (Ib.)
Senior Lecturer
foshiname@yahoo.com

O. E. Oyewole
B.Sc (Ib); M.Sc (Ib); MPH (Ib.); PhD (Ib.)
Senior Lecturer
oyewole2002@yahoo.com

M. A. Titiloye
B.Sc (UNAAB); MPH (Ib.)
Lecturer I
msbnsmtiti@yahoo.com

I. O. Dipeolu
B.Ed.; M.Ed. (Ib); MPH (Ib.)
Lecturer II
oludipeolu@yahoo.com
io.dipeolu@ui.edu.ng

Adeyimika T. Desmennu
B.Sc. (Ib); MPH (Ib.)
Lecturer II
adeyimikades@hotmail.com

Mojisola M. Oluwasanu
B.Sc. (Ib); MPH (Ib.)
Lecturer II
ope3m@yahoo.com

Yetunde O. John-Akinola
B.Sc (Ib.); MPH (Ib.)
Assistant Lecturer
zfisayo@yahoo.com

Appendix 2a. Informed Consent Form

Draft Consent form for Survey Mothers Respondents

Name of Principal Investigator: Dr. Oyedunni S. Arulogun
Name of Co-Principal Investigator: Dr. Rene Lion
Name of Organization: Unilever R & D Vlaardingen
Name of Sponsor: Unilever
Reference Number: SPB01_2015
IRB Research approval number: ####
Approval will elapse by: dd/mm/yyyy

Title of project: Pilot Follow in my Food Steps Behaviour Change Impact Study

Greetings; My name is ___________________________ and I work for the Department of Health Promotion and Education, College of Medicine, University of Ibadan. I am part of a team doing a research study.

The purpose of this research is to understanding what meals are prepared and consumed in homes in Nigeria. Furthermore, we would like to understand the role of knowledge about iron, beliefs about specific ingredients and the social interaction between mother and daughter in determining what meals are consumed and prepared in the home.

Purpose of the research:

We are planning to investigate the effectiveness of a behaviour change intervention aimed at increasing the intake of green leafy vegetables and iron-fortified Knorr Bouillon cubes in Nigerian participants.

Duration of the research:

We expect you to be involved in this research for about 2 hours. One hour today and another hour in about 12 week’s time.

Procedures:

To find answers to some of these questions, we invite you to take part in this research project and participate in an interview. If you accept, you will be asked to participate in an interview with myself. If you do not wish to answer any of the questions posed during the interview, you may say so and the interviewer will move on to the next question. The interview will take place in your house at a time that is convenient for you. No one else but the interviewer will be present. The information recorded is considered confidential, and no one else except Dr. Arulogun and her colleagues will have access to the information documented during your interview.

We will record your answers to these questions on this questionnaire. This is done so that we remember everything that you have told us. Although it is important for the research that you answer all the questions, if you do not wish to answer any of the questions included in the survey, you may ask to move on to the next question.
The information recorded is considered confidential, and no one else except the study coordinators will have access to your answers.

The interview will last for approximately 45 minutes.

Risks and Discomforts:

There is a slight risk that you may feel uncomfortable talking about some of the topics. However, we do not wish this to happen, and you may refuse to answer any question or not take part in a portion of the discussion if you feel the question(s) makes you uncomfortable.

Benefits:

There will be no direct benefit to you but the information obtained from this study will help to provide suggestions that will enable the researchers develop an appropriate intervention program to enable mothers to create healthier meals.

Incentives:

We will provide you with a present of napkin or Unilever product as a token of our appreciation of your time and effort for participating in this interview.

Confidentiality:
We have taken the following steps to ensure that you are safe and that the information you provide is confidential.

1. The interview will take place in a private place as convenient for the respondent, where no one else hears what you discuss with the interviewer.
2. The information that we collect from this research project will be kept confidential.
3. Information collected from you will be stored in a file that will not have your name on it, but a number assigned to it instead.
4. The questionnaire containing the interview will be stored for the duration of the study (2 years) after which it would be destroyed.
5. There will be no name associated with the number assigned to each file will be kept under lock and key and will not be disclosed to anyone except staff working on this study.
6. You may talk to the leader of the research team in case you have any concern or questions.

Alternative to Participation/ Voluntariness:

You do not have to take part in this research if you do not wish to do so, and refusing to participate will not affect you future at this school. Even if you do not wish to answer these questions you may still benefit from the study. You may stop participating in the interview at any time that you wish, and there will be no negative consequences for you in any way.

Who to contact:

If you have any questions you may ask them now or later. If you wish to ask questions later, you may contact any of the following:
Dr. Oyedunni S. Arulogun
Address: Department of Health Promotion and Education, College of Medicine, University of Ibadan
Telephone: 0803-579-4630 or 08057906536
Email: oyedunniarulogun@gmail.com

Certificate of Consent for Quantitative Study

If you agree to participate in this survey interview you can tell us that you agree by repeating these words and then putting your name and signature in the space below.

"I have read the foregoing information, or it has been read to me. I have had the opportunity to ask questions about it and any questions I have asked have been answered to my satisfaction. I voluntarily consent to participate as a subject in this study and understand that I have the right to withdraw from the study at any time without in any way affecting my work position."

______________________	__________	________________	________
Individual Participant's name/ signature	Date	Participant preferred verbal consent confirmed by RA	Date

O. Oladepo
B.Sc (Ib); MPH (Ib.); PhD (Ib.); FRSPH (UK)
Professor
oladepod@yahoo.com
ooladepo@comui.edu.ng

A. J. Ajuwon
B.Sc (Lagos); MPH (Ib.); PhD (Ib.)
Professor
ajajuwon@yahoo.com

J. D. Adeniyi
B.A. (Ife) MPH (Chapel Hill); ARSH (Lond.); Dr. PH (John Hopkins)
Professor/ Adjunct Lecturer
jdadeniyi@yahoo.com

Oyedunin S. Arulogun
B.Ed. (Ib); M.Ed (Ib); MPH (Ib.); PhD (Ib.), FRSPH (UK); CCST (Nig)
Reader
oyedunniarulogun@gmail.com

F. O. Oshiname
B.Sc (Benin); MPH (Ib.); M.A (Cleveland); PhD (Ib.)
Senior Lecturer
foshiname@yahoo.com

O. E. Oyewole
B.Sc (Ib); M.Sc (Ib); MPH (Ib.); PhD (Ib.)
Senior Lecturer
oyewole2002@yahoo.com

M. A. Titiloye
B.Sc (UNAAB); MPH (Ib.)
Lecturer I
msbnsmtiti@yahoo.com

I. O. Dipeolu
B.Ed.; M.Ed. (Ib); MPH (Ib.)
Lecturer II
oludipeolu@yahoo.com
io.dipeolu@ui.edu.ng

Adeyimika T. Desmennu
B.Sc. (Ib); MPH (Ib.)
Lecturer II
adeyimikades@hotmail.com

Mojisola M. Oluwasanu
B.Sc. (Ib); MPH (Ib.)
Lecturer II
ope3m@yahoo.com

Yetunde O. John-Akinola
B.Sc (Ib.); MPH (Ib.)
Assistant Lecturer
zfisayo@yahoo.com

Appendix 2b. Informed Consent Form

Draft Consent form for Survey Daughters Respondents

Name of Principal Investigator: Dr. Oyedunni S. Arulogun
Name of Co-Principal Investigator: Dr. Rene Lion
Name of Organization: Unilever R & D Vlaardingen
Name of Sponsor: Unilever
Reference Number: SPB01_2015
IRB Research approval number: ####
Approval will elapse by: dd/mm/yyyy

Title of project: Pilot Follow in my Food Steps Behaviour Change Impact Study

Greetings; My name is ____________________________ and I work for the Department of Health Promotion and Education, College of Medicine, University of Ibadan. I am part of a team doing a research study.
The purpose of this research is to understanding what meals are prepared and consumed in homes in Nigeria. Furthermore, we would like to understand the role of knowledge about iron, beliefs about specific ingredients and the social interaction between mother and daughter in determining what meals are consumed and prepared in the home.

Purpose of the research:
We are planning to investigate the effectiveness of a behaviour change intervention aimed at increasing the intake of green leafy vegetables and iron-fortified Knorr Bouillon cubes in Nigerian participants.

Duration of the research:

We expect you to be involved in this research for about 2 hours. One hour today and another hour in about 12 week’s time.

Procedures:

To find answers to some of these questions, we invite you to take part in this research project and participate in an interview. If you accept, you will be asked to participate in an interview with myself. If you do not wish to answer any of the questions posed during the interview, you may say so and the interviewer will move on to the next question. The interview will take place in your house at a time that is convenient for you. No one else but the interviewer will be present. The information recorded is considered confidential, and no one else except Dr. Arulogun and her colleagues will have access to the information documented during your interview.

We will record your answers to these questions on this questionnaire. This is done so that we remember everything that you have told us. Although it is important for the research that you answer all the questions, if you do not wish to answer any of the questions included in the survey, you may ask to move on to the next question.
The information recorded is considered confidential, and no one else except the study coordinators will have access to your answers.

The interview will last for approximately 45 minutes.

Risks and Discomforts:

There is a slight risk that you may feel uncomfortable talking about some of the topics. However, we do not wish this to happen, and you may refuse to answer any question or not take part in a portion of the discussion if you feel the question(s) makes you uncomfortable.

Benefits:

There will be no direct benefit to you but the information obtained from this study will help to provide suggestions that will enable the researchers develop an appropriate intervention program to enable mothers to create healthier meals.

Incentives:

We will provide you with a present of napkin or Unilever product as a token of our appreciation of your time and effort for participating in this interview.

Confidentiality:
We have taken the following steps to ensure that you are safe and that the information you provide is confidential.

1. The interview will take place in a private place as convenient for the respondent, where no one else hears what you discuss with the interviewer.
2. The information that we collect from this research project will be kept confidential.
3. Information collected from you will be stored in a file that will not have your name on it, but a number assigned to it instead.
4. The questionnaire containing the interview will be stored for the duration of the study (2 years) after which it would be destroyed.
5. There will be no name associated with the number assigned to each file will be kept under lock and key and will not be disclosed to anyone except staff working on this study.
6. You may talk to the leader of the research team in case you have any concern or questions.

<u>Alternative to Participation/ Voluntariness:</u>

You do not have to take part in this research if you do not wish to do so, and refusing to participate will not affect you future at this school. Even if you do not wish to answer these questions you may still benefit from the study. You may stop participating in the interview at any time that you wish, and there will be no negative consequences for you in any way.

<u>Who to contact:</u>

If you have any questions you may ask them now or later. If you wish to ask questions later, you may contact any of the following:
Dr. Oyedunni S. Arulogun
Address: Department of Health Promotion and Education, College of Medicine, University of Ibadan
Telephone: 0803-579-4630 or 08057906536
Email: oyedunniarulogun@gmail.com

Certificate of Consent for Quantitative Study

If you agree to participate in this survey interview you can tell us that you agree by repeating these words and then putting your name and signature in the space below.

"I have read the foregoing information, or it has been read to me. I have had the opportunity to ask questions about it and any questions I have asked have been answered to my satisfaction. I voluntarily consent to participate as a subject in this study and understand that I have the right to withdraw from the study at any time without in any way affecting my work position."

______________________	______________________	______________________	______________________
Daughter Participant's name/ signature	Mother Participant's name/ signature	Participant preferred verbal consent confirmed by RA	Date

______________________ Date

______________________ Date

Appendix 3. Draft Intervention Programme Radio drama, SMS and IVR programme

Week 1

1. **Radio Drama - EPISODE 1**

Objective: "A flash bulb moment"
Bringing out the fact that iron is important for adolescent girls. The adolescent girls require 50% more iron than normal. The changes which mother might see in their girls and get worried about them (feeling tired, sleeping more) could actually be because of lack of iron in their diets. And they can easily get the additional iron from their diets with minimal changes to existing way of cooking.

2. **IVR 1**

Enroll and ask profiling questions after the initiation event. End with toss-stir-crumble song and reminder on Omotola Stew/New Nigerian Stew. Press 1 to get the song as the ringtone.

Week 2

1. **Radio – Consumer engagement**
2. **SMS 1**

Welcome to the program. Link it to the radio drama-episode 1 (e.g. adolescent girls need 50% more iron than normal). Remind the time of the radio drama, end with toss-stir-crumble.

3. **IVR 2**

Get feedback from the initiation events. What is the one message they remember. What they like and one thing they would like to improve. One thing Omotola learnt (Omotola discussing the flash bulb moment, 50% more iron required/ tiredness of her daughter is due to iron deficiency). Repeat if they don't remember about the iron importance. End with toss-stir-crumble song and reminder on Omotola Stew/New Nigerian Stew.

Week 3

1. **Radio Drama - EPISODE 2**

Objective: Shopping (Planning to cook)
Whenever moms think about buying raw material to prepare stew, they need to think about Knorr and Greens along with it. How to buy the right greens.
What are the 'must have' stops on the shopping trip on a "Stew day". How to make knorr and greens memorable on a stew shopping trip.

Week 4

1. **Radio – consumer engagement - Shopping**
2. **IVR 3**

Personalized greeting (if possible). Ask if they saw the radio drama 2 (if not, ask if they want to listen to the key message/ snippets? Play if they say yes.) Ask if they liked the episode 2 and what was the key message. Ask few things about the radio drama and depending on their response either play the key message again or move to next question. End with toss-stir-crumble song and reminder on Omotola Stew/New Nigerian Stew. Remind that Omotola would be live on radio.

3. **SMS 2**

Link it to the radio drama-episode 2. What is your stew day? Why? Remind the time of the radio drama, end with toss-stir-crumble.

Week 5

1. **Radio Drama - EPISODE 3**

Objective: Mother Daughter bonding. Remind that Omotola would be live next week
Talk about the special bond mom and daughters share. Talk about passing over the good nutritious cooking habits to their daughters-one of the many good habits a mother is supposed to pass over to the daughters. The things which mom learnt about daughter through these bonding session which otherwise they wouldn't know.
How the stew cooking passed on from Omotola's mother to Omotola and now to her daughter. Omotola improvising the age old home recipe to include greens and Knorr to make it even more nutritious without compromising on the taste. Omotola talking about the Stew time being the family time and bonding time and that's the thing which her husband missed the most on his frequent business trip and made him come back sooner from his business trips.

Week 6

1. **Radio – Consumer engagement-Cooking –Omotola Live**
2. **IVR 4**

Personalized greeting (if possible). Ask if they saw the radio drama 3 (if not, ask if they want to listen to it now? Play if they say yes.) Ask if they liked the episode 3 and what was the key message. Ask if they liked Omotola Live show on radio.
Ask few things about the radio drama and depending on their response either play the key message again or move to next question. End with toss-stir-crumble song and reminder on Omotola Stew/New Nigerian Stew.

3. **SMS 3**

Link it to the radio drama-episode 3. Ask one thing which their daughter told them about the school event? Remind the time of the radio drama, end with toss-stir-crumble.

Week 7

1. **Radio Drama - EPISODE 4**

Objective: Cooking

The radio should bring out the key elements of cooking the remix dish. Toss – Stir –Crumble. For a family of 6 we need about 4-5 Knorr cubes. When to add greens, how to cook the greens with stew, when to add Knorr. Bring about the fact that mom and daughter are cooking together and helping out each other making the bond between them even stronger.

Week 8

1. **Radio Drama – Consumer Engagement**
2. **IVR 5**

Personalized greeting (if possible). Ask if they saw the radio drama 4 (if not, ask if they want to listen to it now? Play if they say yes.) Ask if they liked the episode 4 and what was the key message. Ask few things about the radio drama and depending on their response either play the key message again or move to next question. End with toss-stir-crumble song and reminder on Omotola Stew/New Nigerian Stew.

3. **SMS 4**

Link it to the radio drama-episode 4. Ask one question about cooking the Omotola Stew. Remind the time of the radio drama, end with toss-stir-crumble.

Week 9

1. **Radio Drama -EPISODE 5**

Objective: Appreciation and acceptance of the new dish
This episode should bring about the appreciation which moms get for the remixed dish. It should talk about how moms can address any taste queries which the family might raise. Talk about the family time together, mother's role about prioritizing the health of family above all without compromising the taste. Include the idea of "The new Africa/Omotoal Stew". The traditional home stew which is also healthy.

Week 10

1. **Radio – Consumer Engagement**
2. **IVR 6**

Personalized greeting (if possible). Ask if they saw the radio drama 5 (if not, ask if they want to listen to it now? Play if they say yes.) Ask if they liked the episode 5 and what was the key message. Ask few things about the radio drama and depending on their response either play the key message again or move to next question. End with toss-stir-crumble song and reminder on Omotola Stew/New Nigerian Stew.

3. **SMS 5**

Link it to the radio drama-episode 5. Ask one question about how they succeeded in convincing family about Omotola Stew. Remind the time of the radio drama, end with toss-stir-crumble.

Week 11

1. **Radio Drama - EPISODE 6**

Objective: The finale
This is how the new modern Nigeria cooks. The 'Omotola stew'. It is healthier, tastes better. How everyone is already doing it and liking the taste.

Week 12

1. **Radio Drama – Consumer engagement**
2. **IVR 7**

Personalized greeting (if possible). Ask if they saw the radio drama 6 (if not, ask if they want to listen to it now? Play if they say yes.) Ask if they liked the episode 6 and what was the key message. Ask few things about the radio drama and depending on their response either play the key message again or move to next question. End with toss-stir-crumble song and reminder on Omotola Stew/New Nigerian Stew.

3. **SMS 6**

Link it to the radio drama-episode 6. Remind the to listen to the final radio program as the winners would be announced and they can get a chance to meet Omotola, end with toss-stir-crumble

References:

Ajzen, I. (1991). The theory of planned behaviour. *Organizational Behavior and Human Decision Processes, 50,* 179-211.

Ajzen, I. (2001). Nature and operation of attitudes. *Annual Review of Psychology*, 52, 27-58.

Ajzen, I. (2011). The theory of planned behaviour: reactions and reflections. *Psychology &Health, 26*(9), 1113-1127. doi: 10.1080/08870446.2011.613995

Ajzen, I. (2012). The theory of planned behavior. In P. A. M. Lange, A. W. Kruglanski, & E. T. Higgins (Eds.), *Handbook of Theories of Social Psychology* (pp. 438-459). London: Sage.

Ajzen, I. (2014). The theory of planned behaviour is alive and well, and not ready to retire: a commentary on Sniehotta, Presseau, and Araújo-Soares. *Health Psychology Review, 1-7*. doi: 10.1080/17437199.2014.883474

Ajzen, I., & Gilbert-Cote, N. (2008). Attitudes and the prediction of behavior. In W. D. Crano& R. Prislin (Eds.), *Attitudes and Attitude Change* (pp. 289-311). New York: Psychology Press.

Anyika, J. U., Uwaegbute, A. C., Olojede, A. O., &Nwamarah, J. U. (2009). Nutrient intakes for adolescent girls in secondary schools and universities in Abia State of Nigeria, *Pakistan Journal of Nutrition, 8*, 1576-1602.

Bogers, R. P. (2004). Reproducibility, Validity, and Responsiveness to Change of a Short Questionnaire for Measuring Fruit and Vegetable Intake. *American Journal of Epidemiology, 159*, 900-909. doi: 10.1093/aje/kwh123

Danner, U. N., Aarts, H., & de Vries, N. K. (2008). Habit vs. intention in the prediction of future behaviour: the role of frequency, context stability and mental accessibility of past behaviour. *British Journal of Social Psychology, 47*(Pt 2), 245-265. doi: 10.1348/014466607X230876

de Bruijn, G. J. (2010). Understanding college students' fruit consumption. Integrating habit strength in the theory of planned behaviour. *Appetite, 54*, 16-22. doi: 10.1016/j.appet.2009.08.007

de Bruijn, G. J., Kremers, S. P. J., de Vet, E., de Nooijer, J., van Mechelen, W., & Brug, J. (2007). Does habit strength moderate the intention–behaviour relationship in the Theory of Planned Behaviour? The case of fruit consumption. *Psychology &Health, 22*, 899-916. doi: 10.1080/14768320601176113

Ekpo, A. J.,& Jimmy, E. O. (2006). Dietary and haematological evaluation of adolescent females in Nigeria. *Pakistan Journal of Nutrition, 5* (3), 278-281.

Gardner, B., Abraham, C., Lally, P., & de Bruijn, G. J. (2012). Towards parsimony in habit measurement: Testing the convergent and predictive validity of an automaticity subscale of the Self-Report Habit Index. *International Journal of Behavioral Nutrition and Physical Activity, 9*, 102-114. doi: http://www.ijbnpa.org/content/9/1/102

Gardner, B., de Bruijn, G. J., &Lally, P. (2011). A systematic review and meta-analysis of applications of the Self-Report Habit Index to nutrition and physical activity behaviours. *Annals of BehavioralMedicine, 42*(2), 174-187. doi: 10.1007/s12160-011-9282-0

Glew, R. H., Williams, M., Conn, C. A., Cadena, S. M., Crossey, M., Okolo, S. N., &VanderJagt, D. J. (2001). Cardiovascula disease risk factors and diet of Fulani pastoralists of Northern Nigera. *American Journal of Clinical Nutrition, 74*, 730-736.

Greaves, C., Reddy, P., & Sheppard, K. E. (2010). Supporting behaviour change for diabetes prevention. In P. Schwarz, P. Reddy, C. Greaves, J. Dunbar, & J. Schwarz (Eds.), *Diabetes Prevention in Practice* (pp. 19-29). Dresden: Tumaini Institute for Prevention Management.

Hardeman, W., Johnston, M., Johnston, D., Bonetti, D., Wareham, N. J., &Kinmonth, A. L. (2002). Application of the theory of planned behaviour in behaviour change interventions: a systematic review. *Psychology and Health, 17*(2), 123-158. doi: 10.1080/08870440290013644

Lynch, S. R. (2005). The impact of iron fortification on nutritional anaemia. *Lancet*, *18* (2), 333-346.

McEachan, R. R. C., Conner, M., Taylor, N. J., & Lawton, R. J. (2011). Prospective prediction of health-related behaviours with the Theory of Planned Behaviour: a meta-analysis. *Health Psychology Review, 5*(2), 97-144. doi: 10.1080/17437199.2010.521684

Michie, S., Abraham, C., Whittington, C., &McAteer, J. (2009). Effective Techniques in Healthy Eating and Physical Activity interventions: A Meta-Regression. *Health Psychology, 28*, 690-701. doi: 10.1037/a0016136

Nicholson, J. A., Naeeni, M., Hoptroff, M., Matheson, J. R., Roberts, A. J., Taylor, D., . . . Wright, R. L. (2014). An investigation of the effects of a hand washing intervention on health outcomes and school absence using a randomised trial in Indian urban communities. *Tropical Medicine and International Health, 19*(3), 284-292. doi: 10.1111/tmi.12254

Olander, E. K., Fletcher, H., Williams, S., Atkinson, L., Turner, A., & French, D. P. (2013). What are the most effective techniques in changing obese individuals' physical activity self-efficacy and behaviour: a systematic review and meta-analysis.*International Journal of Behavioral Nutrition and Physical Activity, 10*.

Otemyiwa, I. O., &Adewusi, S. R. (2012). Food choice and meal consupmption pattern among undergraduate students in two univesrities in Southwestern Nigeria. *Nutrition & Health, 21* (4), 233-245

Prestwich, A., Sniehotta, F. F., Whittington, C., Dombrowski, S. U., Rogers, L., &Michie, S. (2014). Does theory influence the effectiveness of health behavior interventions? Meta-analysis. *Health Psychology, 33*(5), 465-474. doi: 10.1037/a0032853

Sainsbury, K., Mullan, B. A., & Sharpe, L. (2015). Predicting intention and behaviour following participation in a theory-based intervention to improve gluten free diet adherence in coeliac disease. *Psychology &Health*. doi: 10.1080/08870446.2015.1022548

Sanusi, R. A. (2010). An assessment of dietary diversity in six Nigerian states. *African Journal of Biomedical Research, 13* (3), 161-167.

Stevens, G. A., Finucane, M. M., De-Regil, L. M., Paciorek, C. J., Flaxman, S. R., Branca, F., Pena-Rosas, J. P., Bhutta, Z. A., &Ezzati, M. (2013). Global, regional, and national trends in haemoglobin concentration and prevalence of total and severe anaemia in children and pregnant and non-pregnant women for 1995-2011: a systematic analysis of population-representative data. *Lancet Global Health, 1* (1), e16-e25.

Sniehotta, F. F., Presseau, J., & Araujo-Soares, V. (2014). Time to retire the theory of planned behaviour. Health *Psychological Review, 8*(1), 1-7. doi: 10.1080/17437199.2013.869710

Verplanken, B. (2006). Beyond frequency: habit as mental construct. *British Journal of Social Psychology, 45*(Pt 3), 639-656. doi: 10.1348/014466605X49122

Verplanken, B., & Wood, W. (2006). Interventions to break and create consumer habits. *Journal of Public Policy & Marketing, 25*(1), 90-103.

Webb, T. L., Joseph, J., Yardley, L., &Michie, S. (2010). Using the internet to promote health behavior change: a systematic review and meta-analysis of the impact of theoretical basis, use of behavior change techniques, and mode of delivery on efficacy. *J Med Internet Res, 12(*1), e4. doi: 10.2196/jmir.1376

Webb, T. L., &Sheeran, P. (2006). Does changing intentions engender behaviour change? A meta-analysis of experimental evidence. *Psychological Bulletin*, *132*, 249-268.
