# Supplementary material for: The effect of the “Follow in my Green Food Steps” programme on cooking behaviours for improved iron intake: a quasi-experimental randomized community study
Source: Int J Behav Nutr Phys Act. 2018 Aug 16;15:79. doi: 10.1186/s12966-018-0710-4 (PMC6097342; doi:10.1186/s12966-018-0710-4)
Supplement: Supplementary file 6 — Measurement Models used for Structural Equation Modelling. (PPTX 66 kb) [file 12966_2018_710_MOESM6_ESM.pptx]

## Slide 1
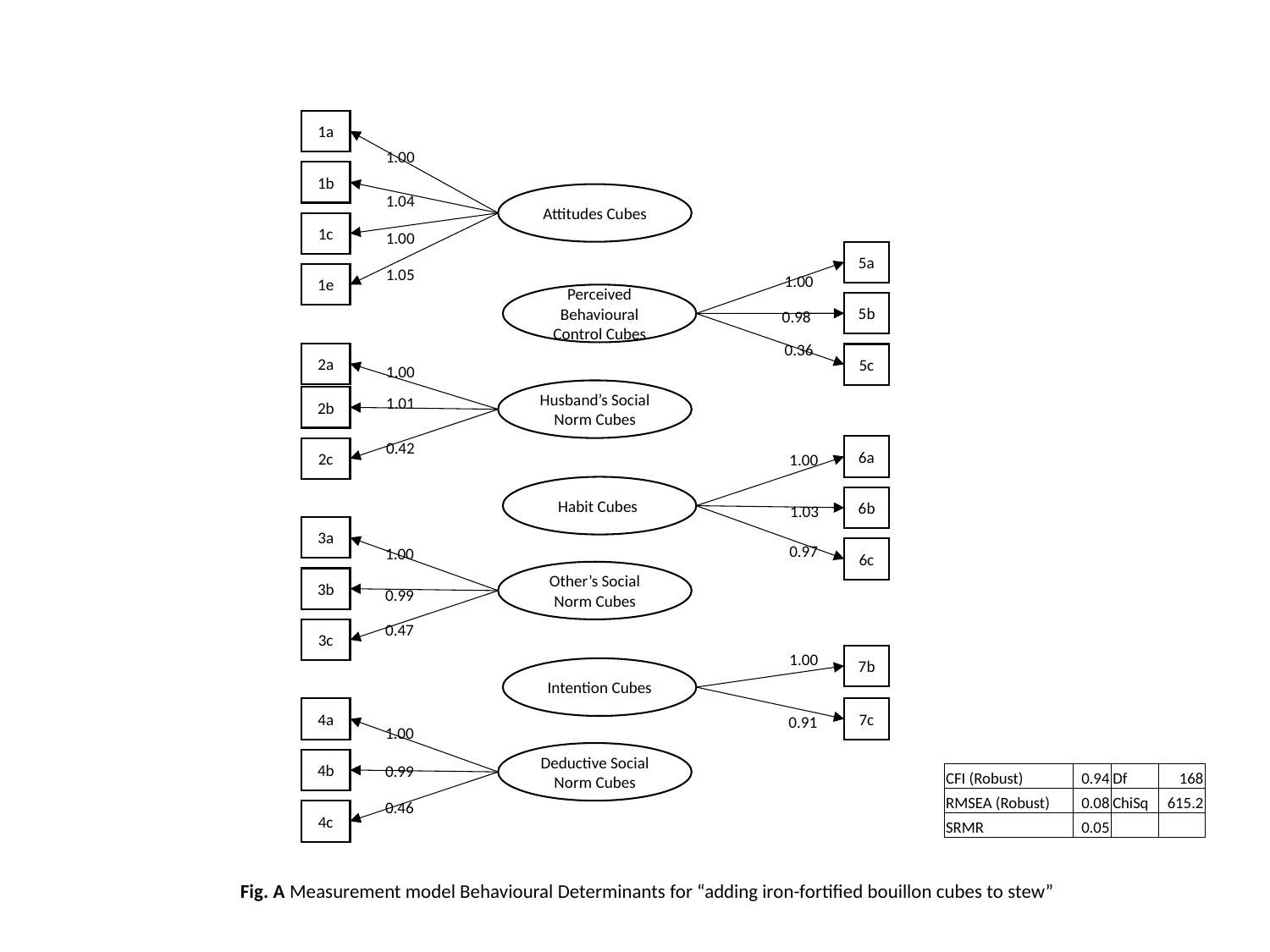

1a
1.00
1b
Attitudes Cubes
1.04
1c
1.00
5a
1.05
1e
1.00
Perceived Behavioural Control Cubes
5b
0.98
0.36
2a
5c
1.00
Husband’s Social Norm Cubes
1.01
2b
0.42
6a
2c
1.00
Habit Cubes
6b
1.03
3a
0.97
1.00
6c
Other’s Social Norm Cubes
3b
0.99
0.47
3c
1.00
7b
Intention Cubes
4a
7c
0.91
1.00
Deductive Social Norm Cubes
4b
0.99
| CFI (Robust) | 0.94 | Df | 168 |
| --- | --- | --- | --- |
| RMSEA (Robust) | 0.08 | ChiSq | 615.2 |
| SRMR | 0.05 | | |
0.46
4c
Fig. A Measurement model Behavioural Determinants for “adding iron-fortified bouillon cubes to stew”

## Slide 2
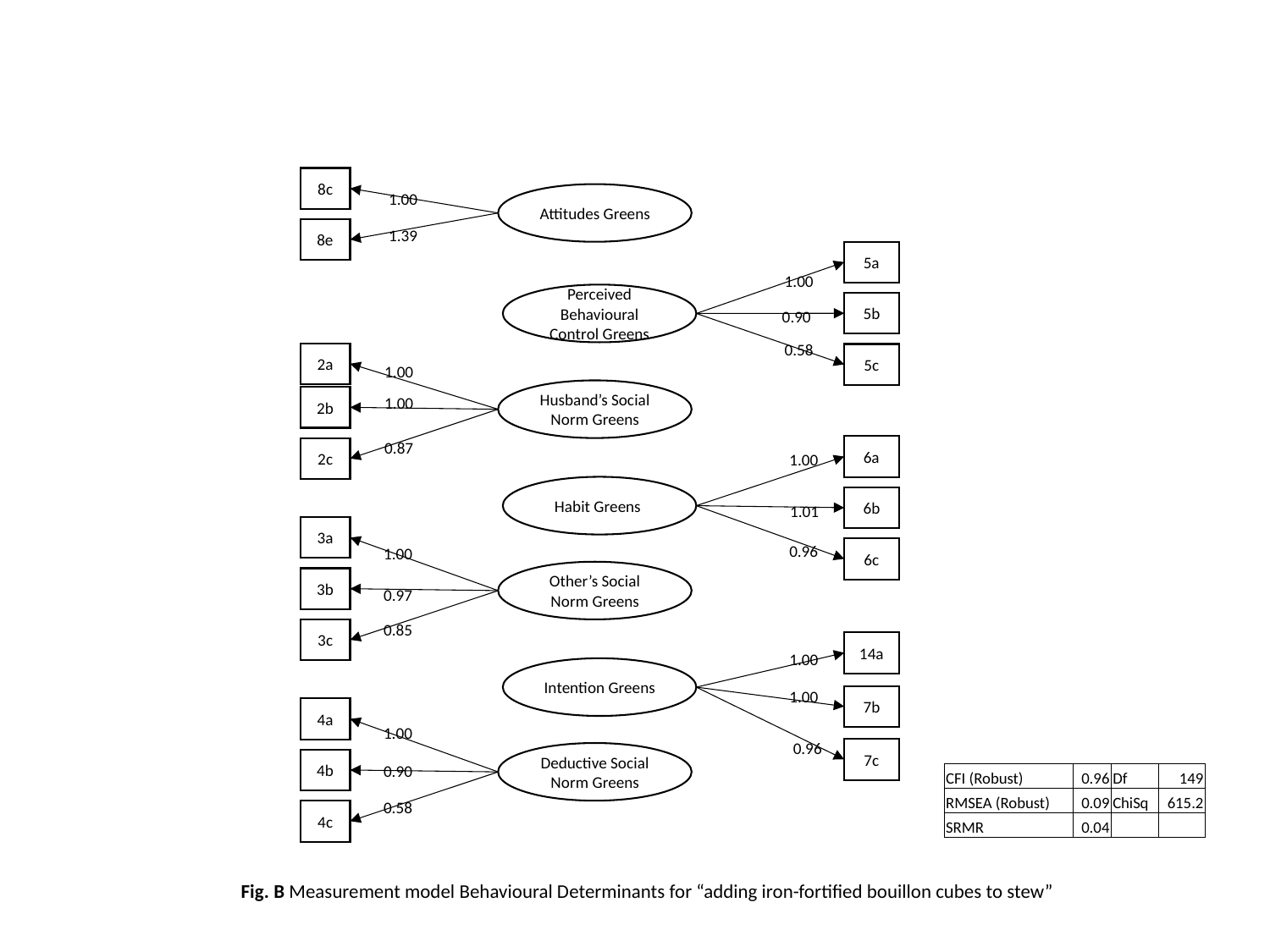

8c
1.00
Attitudes Greens
8e
1.39
5a
1.00
Perceived Behavioural Control Greens
5b
0.90
0.58
2a
5c
1.00
Husband’s Social Norm Greens
1.00
2b
0.87
6a
2c
1.00
Habit Greens
6b
1.01
3a
0.96
1.00
6c
Other’s Social Norm Greens
3b
0.97
0.85
3c
14a
1.00
Intention Greens
1.00
7b
4a
1.00
0.96
7c
Deductive Social Norm Greens
4b
0.90
| CFI (Robust) | 0.96 | Df | 149 |
| --- | --- | --- | --- |
| RMSEA (Robust) | 0.09 | ChiSq | 615.2 |
| SRMR | 0.04 | | |
0.58
4c
Fig. B Measurement model Behavioural Determinants for “adding iron-fortified bouillon cubes to stew”
